# Human ZBP1 is a potent inducer of cell death through mechanisms divergent from mouse ZBP1

**DOI:** 10.1038/s44319-026-00866-6

**Published:** 2026-07-11

**Authors:** Fei Lu, Linghuan Tang, Zichao Tian, Jialiang Tian, Yinghao Fu, Anmin Gao, Heng Dong, Jianxiang Chen, Huasong Lu, Chun Kim, Dengming Lai, Chun Zhou, Jianfeng He, Jinfa Tou, Juan Lin, Huipeng Jiao

**Affiliations:** 1 Department of Neonatal Surgery, Children’s Hospital, Zhejiang University School of Medicine, National Clinical Research Center for Child Health; Life Sciences Institute, Zhejiang University, Hangzhou, 310058 China; 2 Zhejiang Key Laboratory of Molecular Cancer Biology, Life Sciences Institute, Zhejiang University, Hangzhou, 310058 China; 3 State Key Laboratory of Cellular Stress Biology, Cancer Research Center, School of Medicine, Faculty of Medicine and Life Sciences, Xiamen University, Xiamen, 361102 Fujian China; 4 School of Public Health, Zhejiang University School of Medicine, Hangzhou, 310058 China; 5 School of Pharmacy and Department of Hepatology, the Affiliated Hospital of Hangzhou Normal University, Hangzhou Normal University, Hangzhou, Zhejiang 311121 P. R. China; 6 Department of Bio-Pharmaceutical Convergence, Division of Molecular Medicine, Hanyang University [ERICA Campus], Ansan, 15588 Republic of Korea; 7 Department of Neonatal Surgery, Children’s Hospital, Zhejiang University School of Medicine, National Clinical Research Center for Children and Adolescents’ Health and Diseases, Hangzhou, China; 8 Zhejiang Key Laboratory of Neonatal Diseases, Hangzhou, China

**Keywords:** Autophagy & Cell Death, Evolution & Ecology, Immunology

## Abstract

Z-DNA-binding protein 1 (ZBP1) senses Z-form nucleic acids to trigger cell death and inflammation via RHIM domain-mediated interactions with RIPK1 and RIPK3. Here we show that compared to mouse ZBP1 (mZBP1), human ZBP1 (hZBP1) possesses a heightened sensitivity for inducing cell death in cells across different species and potent tumor-killing effects in vivo. In contrast to mZBP1, which signals primarily through RIPK3, hZBP1-induced cell death depends on RIPK1 in a RIPK3-independent manner. Specifically, while the scaffold function of RIPK1 is required for hZBP1-mediated apoptosis, its kinase activity is indispensable for the execution of necroptosis. Unlike mZBP1, which primarily uses only its RHIM1 domain, hZBP1 requires all three RHIM domains (RHIM1, RHIM2, and RHIM3) to trigger cell death. We further identify the C-terminal RHIM2 and RHIM3 regions as the key determinant that confers hZBP1 high sensitivity and confirm that endogenous hZBP1 promotes RIPK1-dependent cell death under pathological conditions. Together, our findings reveal an intrinsic mechanistic divergence between human and murine ZBP1 signaling and highlight the limitations of translating preclinical findings in animal models to human therapeutic strategies targeting this pathway.

## Synopsis

Human ZBP1 is a potent inducer of programmed cell death and inflammation, acting through mechanisms that differ substantially from mouse ZBP1. This study reveals that human ZBP1 signals primarily through a RIPK1-dependent pathway and requires coordinated activity of all three RHIM domains, highlighting important species-specific differences that may affect therapeutic strategies targeting ZBP1 in cancer and inflammatory disease.

- Overexpression of human ZBP1, but not mouse ZBP1, triggers robust spontaneous apoptosis and necroptosis across multiple cell types and shows potent tumor-killing activity in vivo.
- Human ZBP1-induced cell death depends on RIPK1, rather than RIPK3, with RIPK1 scaffold function supporting apoptosis and RIPK1 kinase activity contributing to necroptosis.
- The heightened activity of human ZBP1 is controlled by its C-terminal RHIM2/RHIM3 region, and all three RHIM domains are required for cytoplasmic assembly, RIPK1 recruitment, and downstream cell death signaling.

## Abstract (web summary)

Human ZBP1 is a potent inducer of programmed cell death and inflammation, acting through mechanisms that differ substantially from mouse ZBP1. This study reveals that human ZBP1 signals primarily through a RIPK1-dependent pathway and requires coordinated activity of all three RHIM domains, highlighting important species-specific differences that may affect therapeutic strategies targeting ZBP1 in cancer and inflammatory disease.

## Graphical abstract

**Figure:**
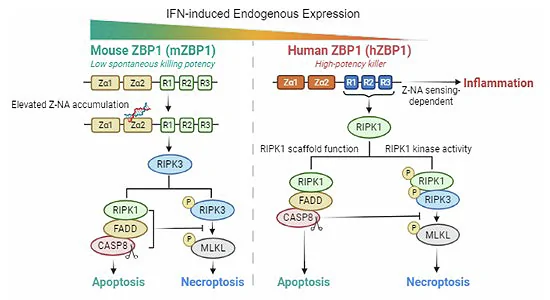

**Figure:**
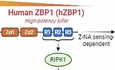

## Introduction

Programmed cell death (PCD) is a cornerstone of host defense and immune regulation, responsible for clearing infected or damaged cells and shaping the inflammatory environment (Galluzzi et al, 2018). Among various PCD pathways, necroptosis and apoptosis are particularly crucial for antiviral responses, tissue homeostasis, and inflammatory diseases. Mechanistically, receptor-interacting serine/threonine-protein kinase 1 (RIPK1) plays a central role. It can either scaffold FADD-caspase-8-dependent apoptosis or, when caspase activity is compromised, partner with RIPK3 to drive mixed-lineage kinase domain-like (MLKL)-mediated necroptosis (Pasparakis and Vandenabeele, 2015). Genetic disruption of RIPK1 regulation precipitates severe skin or intestinal inflammation in mice and causes early-onset immunopathology in humans, underscoring the significance of RIPK1 in tissue homeostasis (Cuchet-Lourenco et al, 2018; Dannappel et al, 2014; Li et al, 2019; Uchiyama et al, 2019).

As one of the central sensors of these cell death pathways, Z-DNA-binding protein 1 (ZBP1) was primarily recognized as a frontline alarm for replicating viruses, such as murine cytomegalovirus (MCMV), influenza A (IAV), vaccinia virus (VACV), and herpes simplex virus 1 (HSV-1) (Guo et al, 2018; Koehler et al, 2017; Koehler et al, 2021; Maelfait et al, 2017; Thapa et al, 2016; Upton et al, 2012; Zhang et al, 2020). During viral infection, ZBP1 senses viral Z-nucleic acids (Z-NAs) via its Zα domains, enabling its RHIM1 to recruit RIPK3 and RIPK1, triggering lytic programmed cell death as an effective antiviral strategy (Guo et al, 2018; Koehler et al, 2017; Thapa et al, 2016; Upton et al, 2012). Paradoxically, however, genetic ablation of *Zbp1* protects mice from SARS-CoV-2-induced tissue injury, highlighting the dual nature of ZBP1 signaling during viral infection (Karki et al, 2022; Li et al, 2023).

Beyond its role in viral defense, ZBP1 is a crucial driver of sterile inflammation by aberrantly sensing self Z-NAs. For example, SETDB1 deficiency causes accumulation of dsRNA derived from endogenous retroviral elements (EREs), which activates ZBP1-mediated necroptosis, accelerating inflammatory bowel disease (Wang et al, 2020). Similarly, mutations in the dsRNA-editing enzyme ADAR1 lead to accumulation of unedited dsRNA that chronically activates ZBP1, causing severe systemic autoinflammation in mice (de Reuver et al, 2022; Hubbard et al, 2022; Jiao et al, 2022). Interestingly, ZBP1’s ability to promote pathogenic IFN responses occurs independently of the cell death machinery(de Reuver et al, 2022; Hubbard et al, 2022; Jiao et al, 2022). Furthermore, during heat stress, ZBP1 activation depends on its RHIM domains-mediated aggregation, but not its Z-NAs sensing activity, suggesting a distinct mechanism for ZBP1’s roles in inflammation and cell death (Yuan et al, 2022).

Despite these detailed insights from mouse studies, the mechanistic role of human ZBP1 (hZBP1) remains insufficiently understood. Emerging evidence suggests that while mZBP1 primarily engages RIPK3 and MLKL to execute necroptosis in the absence of RIPK1, RIPK1 is essential for forming a stable and functional ZBP1-RIPK3 complex in human cells (Amusan et al, 2025). Furthermore, hZBP1 seems to have a lower activation threshold than its mouse counterpart, driving inflammatory signaling without cell death even in the absence of strong external stimuli (Peng et al, 2022). Clinical evidence demonstrated that ADAR1 mutations, which increase Z-RNA, cause Aicardi-Goutières syndrome (AGS) in humans, manifested with intracranial calcification and systemic inflammation (Rice et al, 2012). While these findings echo murine models, they raise concerns about how well preclinical ZBP1 studies translate to human biology.

In this study, we highlight hZBP1 as a potent inducer of inflammation and programmed cell death that functions distinctly from its mouse counterpart. We show that hZBP1 can directly recruit RIPK1 for cell death signaling, bypassing the typical RIPK3 dependency seen in mice. This redefines how ZBP1 induces necroinflammation. Mechanistically, we found that while mZBP1 dominantly uses its RHIM1 domain to initiate necroptosis, hZBP1 requires a broader set of interactions involving its RHIM1, RHIM2, and RHIM3 domains. This tripartite dependence facilitates the formation of stable cytoplasmic aggregates and RIPK1 recruitment even without RIPK3. Through domain-swap experiments, we pinpointed the C-terminal region containing RHIM2 and RHIM3 as the key determinant of hZBP1’s unique sensitivity and aggregation behavior. These findings not only question the direct applicability of mouse models to human inflammatory diseases but also establish hZBP1 as a critical therapeutic target for severe inflammatory and autoimmune disorders.

## Results

### Overexpression of human ZBP1 triggers potent cell death via RIPK1

**Figure 1:**
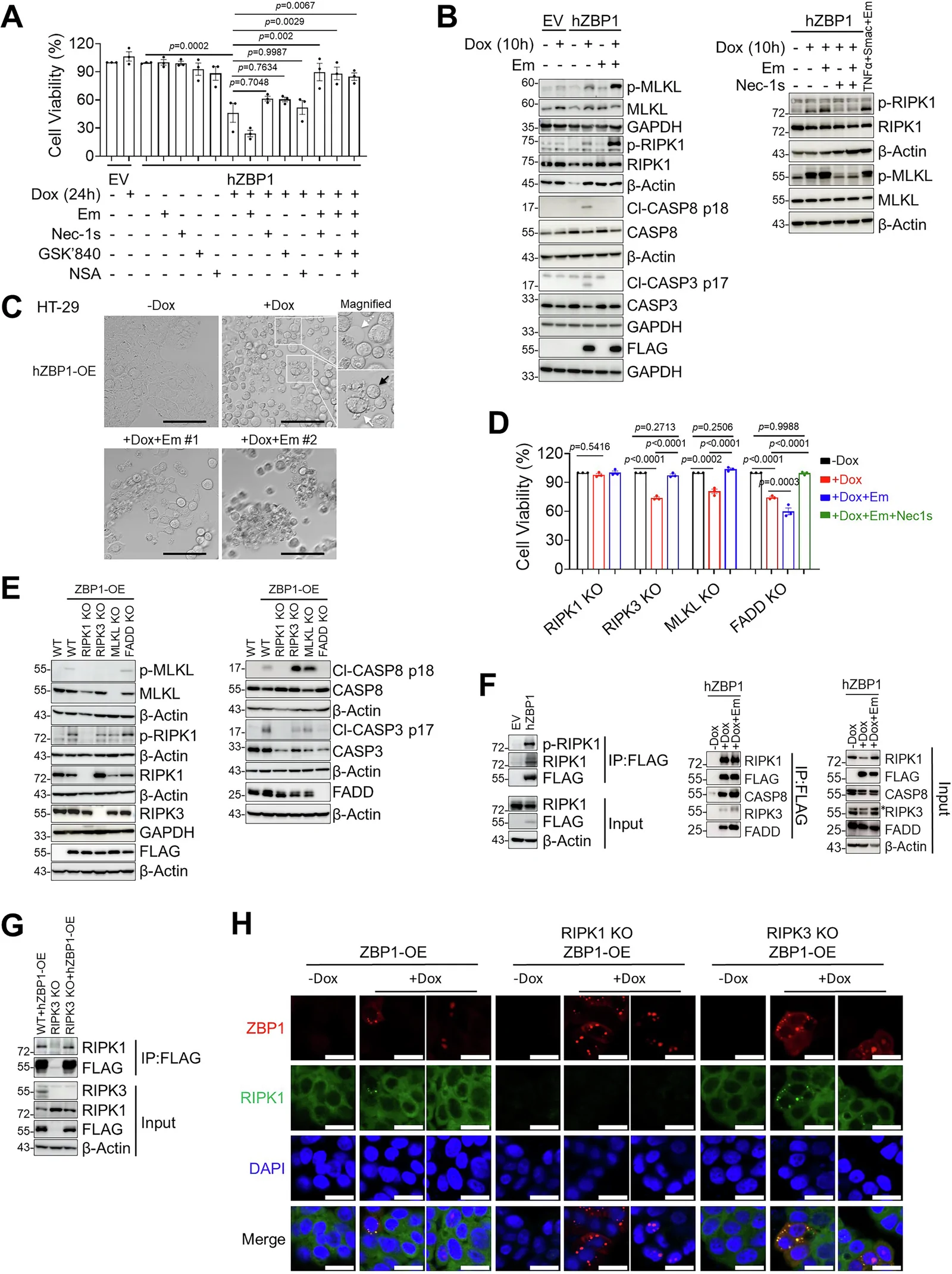
Human ZBP1 triggers spontaneous cell death via RIPK1. (**A**) Cell viability measured by neutral red uptake in HT-29 expressing Dox-inducible FLAG-EV, FLAG-hZBP1 stimulated with or without Dox (1 μg/mL) or combinations of Dox and emricasan (5 μM), Nec-1s (10 μM), GSK’840 (3 μM), and NSA (5 μM) for 24 h. (**B**) Immunoblot analysis of total lysates in HT-29 expressing Dox-inducible FLAG-EV, FLAG-hZBP1 stimulated with Dox (1 μg/mL), a combination of Dox (1 μg/mL) and emricasan (5 μM), or Nec-1s (10 μM) for 10 h. Lysates of HT-29 cells treated with TNFα+Smac+Em for 4 h were loaded as a positive control for necroptosis. (**C**) Representative cell images of HT-29 expressing Dox-inducible FLAG-hZBP1 stimulated with or without Dox (1 μg/mL) or combinations of Dox (1 μg/mL) and emricasan (5 μM) for 10 h. Representative cells in white square boxes were magnified; the white solid arrow indicates the apoptotic cell, the black solid arrow indicates the necroptotic cell, and the white dotted arrow indicates the pyroptotic-like cell. Representative views of adherent cells and suspension cells in Dox+Em group were displayed and were annotated as “#1” and “#2” respectively. Scale bars, 100 μm. (**D**) Cell viability measured by neutral red uptake in RIPK1 KO, RIPK3 KO, MLKL KO, and FADD KO HT-29 expressing Dox-inducible FLAG-hZBP1 stimulated with or without Dox (1 μg/mL) or combinations of Dox and emricasan (5 μM) or Nec-1s (10 μM) for 24 h. (**E**) Immunoblot analysis of total lysates in WT, RIPK1 KO, RIPK3 KO, MLKL KO, and FADD KO HT-29 expressing Dox-inducible FLAG-hZBP1 stimulated with Dox (1 μg/mL) for 10 h. (**F**) Immunoblot analysis of FLAG immunoprecipitates and total lysates (Input) from HT-29 expressing Dox-inducible FLAG-EV and FLAG-hZBP1 stimulated with Dox (1 μg/mL) or combinations of Dox (1 μg/mL) and emricasan (5 μM) for 10 h. * nonspecific band. (**G**) Immunoblot analysis of FLAG immunoprecipitates and total lysates (Input) from WT, RIPK3 KO HT-29 expressing Dox-inducible FLAG-hZBP1 stimulated with or without Dox (1 μg/mL) for 12 h. (**H**) Representative confocal images of WT, RIPK1 KO, and RIPK3 KO HT-29 expressing Dox-inducible FLAG-hZBP1 stimulated with or without Dox (1 μg/mL) for 10 h. Scale bars, 20 μm. All experiments were replicated at least three times. Data with error bars are mean ± SEM. Data in panels (**A**, **D**) are pooled and analyzed from at least three independent biological experiments. *P* values were calculated by one-way ANOVA with Tukey’s post hoc test for multiple comparisons (**A**, **D**). Source data are available online for this figure.

**Figure EV1:**
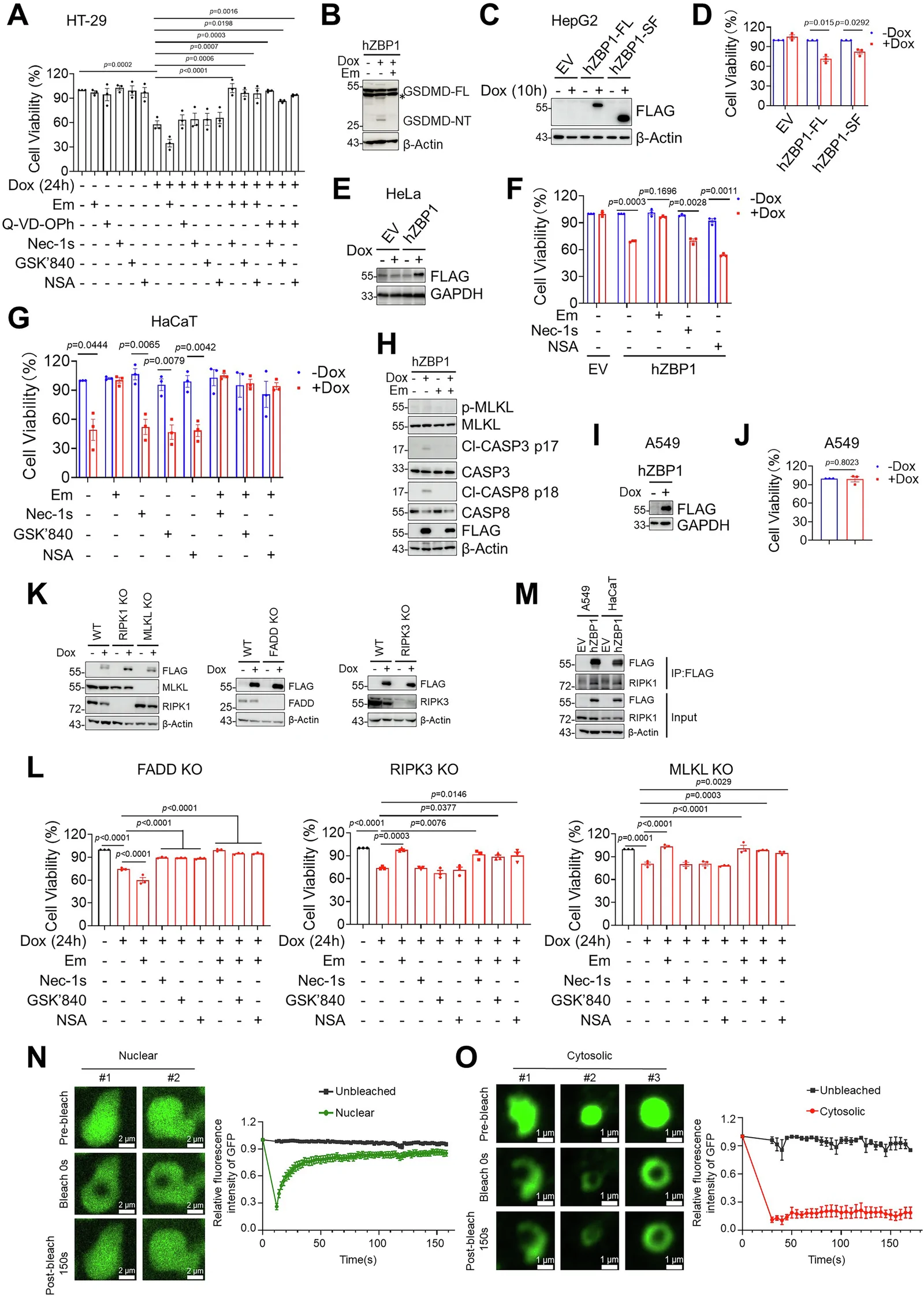
Human ZBP1 triggers spontaneous cell death via RIPK1. (**A**) Cell viability measured by neutral red uptake in HT-29 expressing Dox-inducible FLAG-EV, FLAG-hZBP1 stimulated with or without Dox (1 μg/mL) or combinations of Dox and emricasan (5 μM), Q-VD-OPh (10 μM), Nec-1s (10 μM), GSK’840 (3 μM), and NSA (5 μM) for 24 h. (**B**) Immunoblot analysis of total lysates in HT-29 expressing Dox-inducible FLAG-hZBP1 stimulated with or without Dox (1 μg/mL) or combinations of Dox (1 μg/mL) and emricasan (5 μM) for 10 h. * nonspecific band. (**C**) Immunoblot analysis of total lysates in HepG2 expressing Dox-inducible FLAG-EV, hZBP1-FL, and hZBP1-SF stimulated with or without Dox (1 μg/mL) for 10 h. (**D**) Cell viability measured by neutral red uptake in HepG2 expressing Dox-inducible FLAG-EV, hZBP1-FL, and hZBP1-SF stimulated with or without Dox (1 μg/mL) for 24 h. (**E**) Immunoblot analysis of total lysates in HeLa expressing Dox-inducible FLAG-EV and hZBP1 stimulated with or without Dox (1 μg/mL) for 10 h. (**F**) Cell viability measured by neutral red uptake in HeLa expressing Dox-inducible FLAG-EV and FLAG-hZBP1 stimulated with or without Dox (1 μg/mL) or combinations of Dox and emricasan (5 μM), Nec-1s (10 μM), and NSA (2 μM) for 48 h. (**G**) Cell viability measured by neutral red uptake in HaCaT expressing Dox-inducible FLAG-hZBP1 stimulated with or without Dox (1 μg/mL) or combinations of Dox and emricasan (5 μM), Nec-1s (10 μM), GSK’840 (3 μM), and NSA (5 μM) for 24 h. (**H**) Immunoblot analysis of total lysates in HaCaT expressing Dox-inducible FLAG-EV and hZBP1 stimulated with Dox (1 μg/mL) or a combination of Dox (1 μg/mL) and emricasan (5 μM) for 10 h. (**I**) Immunoblot analysis of total lysates in A549 expressing Dox-inducible FLAG-hZBP1 stimulated with or without Dox (1 μg/mL) for 10 h. (**J**) Cell viability measured by neutral red uptake in A549 expressing Dox-inducible FLAG-hZBP1 stimulated with or without Dox (1 μg/mL) for 24 h. (**K**) Immunoblot analysis of total lysates in WT, RIPK1 KO, RIPK3 KO, MLKL KO, and FADD KO HT-29 expressing Dox-inducible FLAG-hZBP1 stimulated with or without Dox (1 μg/mL) for 10 h. (**L**) Cell viability measured by neutral red uptake in FADD KO, RIPK3 KO, and MLKL KO HT-29 expressing Dox-inducible FLAG-hZBP1 stimulated with or without Dox (1 μg/mL), or combinations of Dox and emricasan (5 μM), Nec-1s (10 μM), GSK’840 (3 μM), and NSA (5 μM) for 24 h. (**M**) Immunoblot analysis of FLAG immunoprecipitates and total lysates (Input) from A549 and HaCaT expressing Dox-inducible FLAG-EV and FLAG-hZBP1 stimulated with Dox (1 μg/mL) for 12 h. (**N**,** O**) FRAP analysis of GFP-hZBP1 assemblies formed in the nucleus (**N**) and cytosol (**O**) in HT-29 expressing Dox-inducible GFP-hZBP1 stimulated with Dox (1 μg/mL) for 10 h. Left, representative confocal images of assemblies before and after bleaching. Right, quantification of the GFP fluorescence recovery curves. Scale bars, 2 μm in (**N**) and 1 μm in (**O**). All experiments were replicated at least three times. Data with error bars are mean ± SEM. Data in panels (**A**, **D**, **F**, **G**, **J**, **L**) are pooled and analyzed from at least three independent biological experiments. *P* values were calculated by unpaired parametric *t*-test with Welch’s correction (**D**, **F**, **G**, **J**) or one-way ANOVA with Tukey’s post hoc test for multiple comparisons (**A**, **L**). Source data are available online for this figure.

To investigate the mechanism of hZBP1-induced cell death, we generated HT-29 cells that overexpress FLAG-tagged hZBP1 via a doxycycline (Dox)-inducible system. Surprisingly, unlike a previous study that showed mZBP1 overexpression did not cause spontaneous cell death in immortalized murine embryonic fibroblasts (MEFs) (Jiao et al, 2020), we observed significant cell death within 24 h of Dox treatment in HT-29 cells, HepG2 cells, HeLa cells, and HaCaT cells (Figs. 1A–C and EV1A–H). The cell death in HT-29 cells was not prevented by individual treatment with RIPK1 inhibitor Nec-1s, RIPK3 inhibitor GSK'840, MLKL inhibitor Necrosulfonamide (NSA), or caspase inhibitors Emricasan (Em) and Q-VD-OPh (Figs. 1A and EV1A), indicating hZBP1-induced cell death is independent of RIPK1 kinase activity. However, combined treatment with caspase inhibitors (Em or Q-VD-OPh) with Nec-1s, GSK'840, or NSA almost completely prevented hZBP1-induced cell death (Figs. 1A and EV1A), suggesting hZBP1 triggers programmed cell death involving both apoptosis and necroptosis, where RIPK1 kinase activity is indispensable for necroptosis induction when caspase activities are blocked. Consistently, hZBP1 overexpression activated programmed cell death pathways, evidenced by the phosphorylation of RIPK1 and MLKL and the cleavage of caspase-8 and caspase-3 (Fig. 1B). Notably, HT-29 cells overexpressing hZBP1 exhibited morphological hallmarks of apoptosis, including membrane blebbing and the formation of apoptotic bodies—features notably absent in Em-treated cells (Fig. 1C). Concurrently, hZBP1 overexpression induced typical necroptotic features, such as cell swelling and plasma membrane rupture, which were further exacerbated by caspase inhibition (Fig. 1C). Furthermore, a subset of hZBP1-expressing cells displayed characteristics of pyroptosis, identified by the development of translucent, vesicular membrane blebs (Fig. 1C). This observation aligns with our finding that hZBP1 also induces GSDMD cleavage, which has been reported to be cleavable by caspase-8(Orning et al, 2018)—a process effectively inhibited by Em treatment (Fig. EV1B). However, Em treatment alone, but not Nec-1s, GSK'840, or NSA, prevented hZBP1-induced cell death in HaCaT cells and HeLa cells (Fig. EV1F,G). This suggests that hZBP1 overexpression predominantly induces apoptosis in HaCaT cells and HeLa cells. Accordingly, hZBP1 overexpression led to the cleavage of caspase-8 and caspase-3, which were blocked by Em pretreatment, but not the phosphorylation of MLKL in HaCaT cells (Fig. EV1H). This discrepancy with HT-29 cells may be due to the different expression levels of RIPK3 in these cell lines, which is essential for necroptosis induction. These results strongly suggested that hZBP1 overexpression triggers spontaneous regulated apoptosis and necroptosis.

Previous reports showed that mZBP1 primarily activates both FADD-caspase-8-mediated apoptosis and MLKL-mediated necroptosis via RIPK3(Thapa et al, 2016). To further elucidate the downstream events activated by hZBP1, we generated stable HT-29 cells specifically knocked out (KO) of RIPK1, RIPK3, MLKL, and FADD, followed by overexpression with FLAG-hZBP1 (Figs. 1D,E and EV1K). Unexpectedly, we found that RIPK1 deficiency, but not RIPK3, MLKL, or FADD deficiency, eliminated hZBP1-induced cell death and the associated activation of cell death markers (Fig. 1D,E), suggesting the critical role of RIPK1 in modulating hZBP1-induced downstream signaling. Moreover, hZBP1-induced cell death in RIPK3 KO and MLKL KO cells was prevented by inhibiting caspase-8 activity (Figs. 1D and EV1L). Similar to results obtained with caspase inhibition (Figs. 1A and EV1A), hZBP1-induced cell death in FADD KO cells were inhibited by RIPK1 kinase inhibitor Nec-1s or necroptosis inhibitors GSK'840 or NSA (Figs. 1D,E and EV1L). A minor fraction of residual cell death in FADD KO cells was further attenuated by the addition of Em (Fig. EV1L), suggesting that the limited caspase-8 activity facilitates cell death independently of FADD. Interestingly, we observed that Em treatment mildly exacerbated cell death in FADD KO HT-29 cells (Figs. 1D and EV1L), suggesting that, even in the absence of the FADD scaffold, basal caspase-8 proteolytic activity persists—potentially at levels below the detection limit of immunoblotting (Fig. 1E). This residual activity may exert a pro-survival role by mediating the proteolytic cleavage and inactivation of RIPK1 and RIPK3. However, the underlying regulatory mechanisms governing this FADD-independent caspase-8 activation remain to be fully elucidated. Nevertheless, these findings suggest that, in contrast to mZBP1, hZBP1 primarily uses RIPK1 for downstream signaling activation.

To characterize the molecular complexes formed upon hZBP1 overexpression in HT-29 cells, we performed immunoprecipitation (IP) assays. The results demonstrated that hZBP1 robustly recruits a multifaceted signaling complex comprising RIPK1, RIPK3, FADD, and caspase-8 with or without Em pretreatment (Fig. 1F). Importantly, hZBP1 interacts with RIPK1 independently of RIPK3 (Fig. 1F,G). Consistent with these results, hZBP1 formed cytoplasmic puncta that co-localized with RIPK1 in the absence of RIPK3 (Fig. 1H). Interestingly, hZBP1 puncta were also observed in the nucleus, but these nuclear structures did not co-localize with RIPK1, suggesting distinct dynamic and functional properties of ZBP1 signaling complexes in different cellular compartments (Fig. 1H). hZBP1 also interacts with RIPK1 in other human cell lines, including HaCaT cells and A549 cells, although its overexpression did not trigger cell death in A549 cells (Figs. EV1I,J,M). To further characterize the nuclear and cytoplasmic ZBP1 assemblies, we performed fluorescence recovery after photobleaching (FRAP) assays. The nuclear assemblies displayed droplet-like properties, consistent with liquid-phase condensates, while the cytoplasmic puncta formed with RIPK1 exhibited a solid-like nature (Fig. EV1N,O). Although the biological significance of the solid-like cytoplasmic assemblies remains unclear, these observations suggest that RHIM-dependent interactions between hZBP1 and RIPK1 may drive the formation of a functional amyloidal signaling complex, contributing to the stabilization of solid-state signaling structures. Interestingly, while RIPK1 kinase activity was not required for hZBP1-induced cell death, RIPK1 bound to hZBP1 was found to be phosphorylated (Figs. 1A,B,F and EV1A,F,G). Indeed, MLKL phosphorylation was eliminated upon Nec-1s treatment in HT-29 overexpressing hZBP1 (Fig. 1B), demonstrating that RIPK1 kinase activity is a prerequisite for hZBP1-mediated necroptosis but redundant for hZBP1-induced apoptosis. Thus, this RIPK1 phosphorylation may facilitate the recruitment of RIPK3 to the signaling complex, thereby promoting ensuing necroptosis.

### Overexpression of human ZBP1 exerts potent anti-tumor effects in vivo

**Figure EV2:**
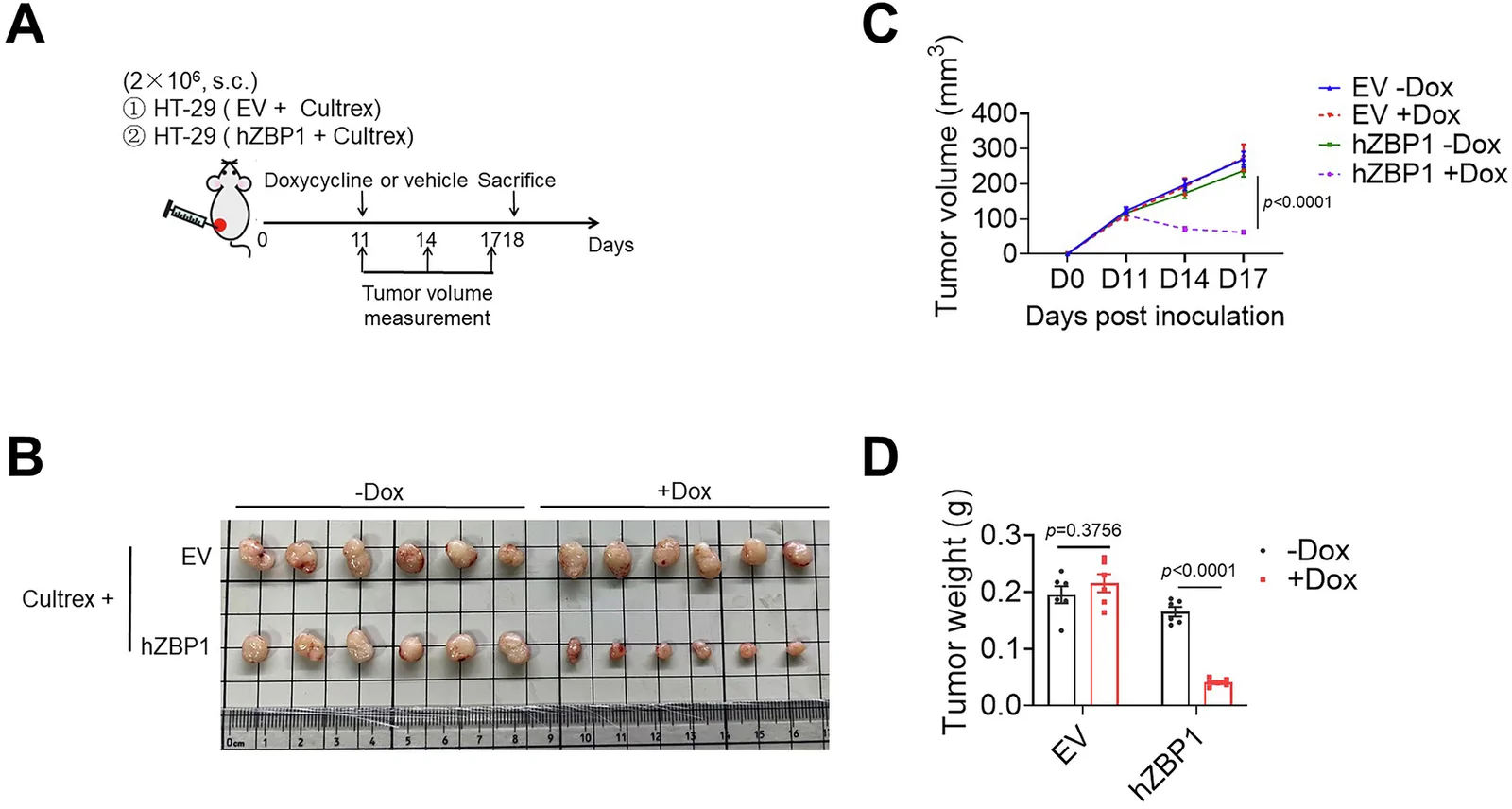
Human ZBP1 exerts potent anti-tumor effects in vivo. (**A**) Treatment schedule of nude mice bearing subcutaneous (s.c.) HT-29. Treatments were initiated at day 11 post inoculation when tumors were palpable (~100 mm^3^). (**B**) Picture representation of tumors at day 18 post inoculation. (**C**) Individual tumor growth curves of Dox-treated and untreated HT-29 tumors at the indicated time points. (**D**) The tumor weight of nude mice bearing HT-29 treated with or without Dox. *n* = 6 mice for each group. Data with error bars are mean ± SEM. *P* values were calculated by unpaired parametric *t*-test with Welch’s correction (**C**, **D**). Source data are available online for this figure.

To investigate the cell death-promoting effects of hZBP1 expression in vivo, we injected inducible EV- or hZBP1-overexpressing HT-29 cells into BALB/c nude mice (Fig. EV2A). The tumors showed similar growth levels before doxycycline treatment (Fig. EV2B,C). As anticipated, EV-expressing tumors showed sustained growth after doxycycline treatment (Fig. EV2B,C). In stark contrast, however, the sizes of hZBP1-overexpressing tumors were significantly reduced, with markedly smaller volumes than the untreated controls at all examined time points (Fig. EV2B,C). On day 18 post inoculation, we harvested the tumors for measurement. The tumors from the hZBP1-overexpressing group had significantly reduced sizes and consistently lower weights compared to both untreated controls and the EV-expressing tumor groups (Fig. EV2B–D). These results extend our in vitro findings, supporting that hZBP1 overexpression triggers potent cell death. This effect manifested as a striking ability to kill established tumors in a cell-derived xenograft (CDX) mouse model in vivo, providing strong evidence for hZBP1 as a promising target for novel anti-cancer therapies.

### C-terminal RHIM domains enable hZBP1 to potently induce cell death

**Figure EV3:**
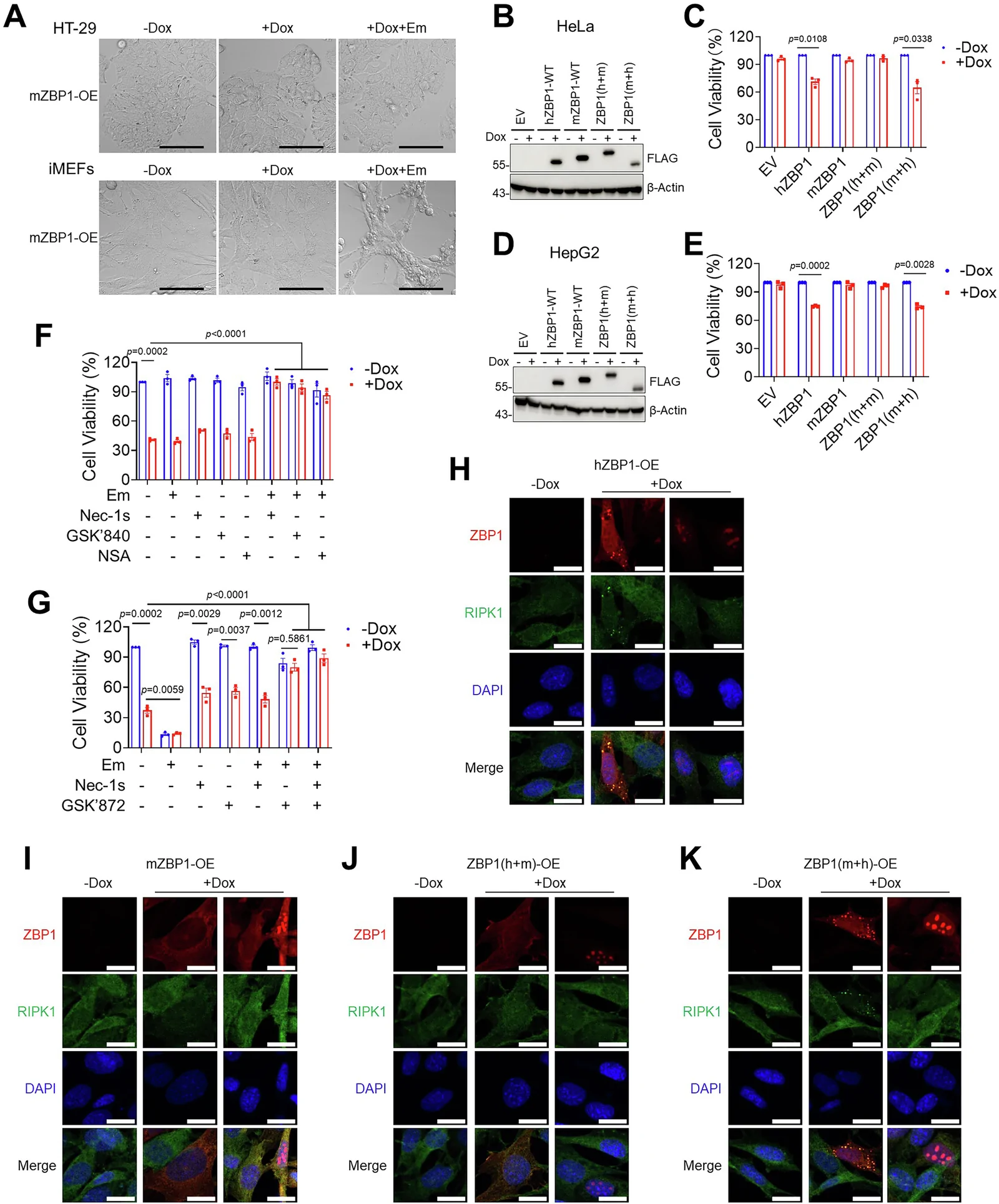
C-terminal RHIM domains enable hZBP1 to induce spontaneous cell death. (**A**) Representative cell images of HT-29 cells expressing Dox-inducible FLAG-mZBP1 (top) and immortalized MEFs expressing Dox-inducible FLAG-mZBP1 (bottom) stimulated with or without Dox (1 μg/mL) or combinations of Dox (1 μg/mL) and emricasan (5 μM) for 10 h. Scale bars, 100 μm. (**B**, **D**) Immunoblot analysis of total lysates in HeLa (**B**) and HepG2 (**D**) expressing Dox-inducible FLAG-EV, FLAG-hZBP1, FLAG-mZBP1, FLAG-ZBP1(h + m), and FLAG-ZBP1(m + h) stimulated with or without Dox (1 μg/mL) for 10 h. (**C**, **E**) Cell viability measured by neutral red uptake in HeLa (**C**) and HepG2 (**E**) expressing Dox-inducible FLAG-EV, FLAG-hZBP1, FLAG-mZBP1, FLAG-ZBP1(h + m), and FLAG-ZBP1(m + h) stimulated with or without Dox (1 μg/mL) for 24 h. (**F**) Cell viability measured by neutral red uptake in HT-29 expressing Dox-inducible FLAG-ZBP1(m + h) stimulated with or without Dox (1 μg/mL) or combinations of Dox and emricasan (5 μM), Nec-1s (10 μM), GSK’840 (3 μM), and NSA (5 μM) for 24 h. (**G**) Cell viability measured by neutral red uptake in immortalized MEFs expressing Dox-inducible FLAG-ZBP1(m + h) stimulated with or without Dox (1 μg/mL) or combinations of Dox and emricasan (5 μM), Nec-1s (10 μM), and GSK’872 (3 μM) for 24 h. (**H**–**K**) Representative confocal images of immortalized MEFs expressing Dox-inducible FLAG-hZBP1 (**H**), FLAG-mZBP1 (**I**), FLAG-ZBP1(h + m) (**J**), and FLAG-ZBP1(m + h) (**K**) stimulated with or without Dox (1 μg/mL) for 10 h. Scale bars, 20 μm. All experiments were replicated at least three times. Data with error bars are mean ± SEM. Data in panels (**C**, **E**, **F**, **G**) are pooled and analyzed from at least three independent biological experiments. *P* values were calculated by unpaired parametric *t*-test with Welch’s correction (**C**, **E**) or one-way ANOVA with Tukey’s post hoc test for multiple comparisons (**F**, **G**). Source data are available online for this figure.

**Figure 2:**
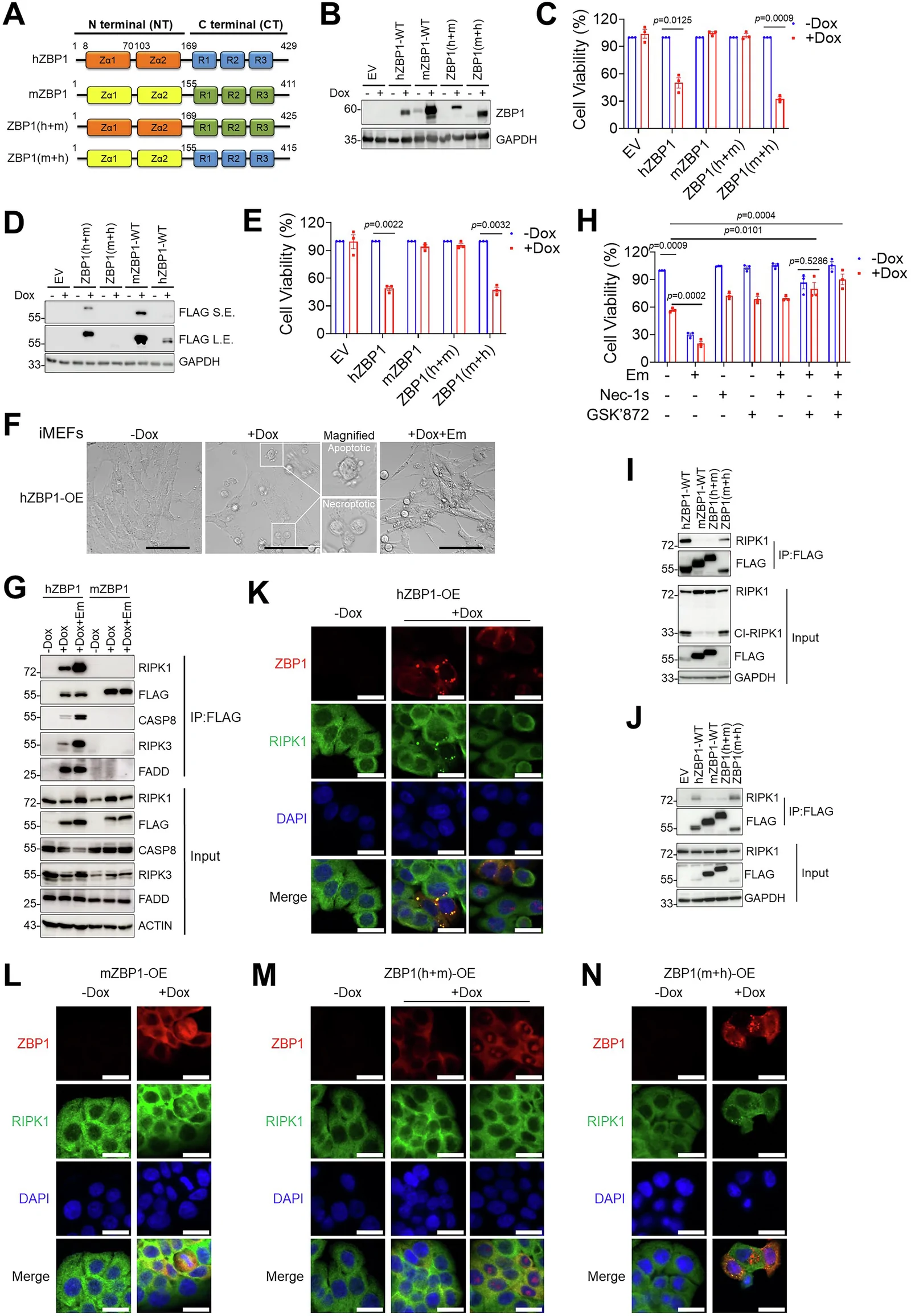
C-terminal RHIM domains enable hZBP1 to induce spontaneous cell death. (**A**) Schematic illustration of FLAG-tagged hZBP1, mZBP1, ZBP1(h + m), and ZBP1(m + h) chimeric variants. (**B**, **D**) Immunoblot analysis of total lysates in HT-29 (**B**) and immortalized MEFs (**D**) expressing Dox-inducible FLAG-EV, FLAG-hZBP1, FLAG-mZBP1, FLAG-ZBP1(h + m), and FLAG-ZBP1(m + h) stimulated with or without Dox (1 μg/mL) for 10 h. (**C**, **E**) Cell viability measured by neutral red uptake in HT-29 (**C**) and immortalized MEFs (**E**) expressing Dox-inducible FLAG-EV, FLAG-hZBP1, FLAG-mZBP1, FLAG-ZBP1(h + m), and FLAG-ZBP1(m + h) stimulated with or without Dox (1 μg/mL) for 24 h. (**F**) Representative cell images of immortalized MEFs expressing Dox-inducible FLAG-hZBP1 stimulated with or without Dox (1 μg/mL) or combinations of Dox (1 μg/mL) and emricasan (5 μM) for 10 h. A representative apoptotic cell and necroptotic cell in white square boxes were magnified. Scale bars, 100 μm. (**G**) Immunoblot analysis of FLAG immunoprecipitates and total lysates (Input) from HT-29 expressing Dox-inducible FLAG-tagged hZBP1 or mZBP1 stimulated with or without Dox (1 μg/mL) or combinations of Dox (1 μg/mL) and emricasan (5 μM) for 10 h. (**H**) Cell viability measured by neutral red uptake in immortalized MEFs expressing Dox-inducible FLAG-hZBP1 stimulated with or without Dox (1 μg/mL) or combinations of Dox and emricasan (5 μM), Nec-1s (10 μM), or GSK’872 (3 μM) for 24 h. (**I**, **J**) Immunoblot analysis of FLAG immunoprecipitates and total lysates (Input) from HT-29 (**I**) and immortalized MEFs (**J**) expressing Dox-inducible FLAG-tagged ZBP1 chimeric variants stimulated with Dox (1 μg/mL) for 12 h. (**K**–**N**) Representative confocal images of HT-29 expressing Dox-inducible FLAG-hZBP1 (**K**), FLAG-mZBP1 (**L**), FLAG-ZBP1(h + m) (**M**), and FLAG-ZBP1(m + h) (**N**) stimulated with or without Dox (1 μg/mL) for 10 h. Scale bars, 20 μm. All experiments were replicated at least three times. Data with error bars are mean ± SEM. Data in panels (**C**, **E**, **H**) are pooled and analyzed from at least three independent biological experiments. *P* values were calculated by unpaired parametric *t*-test with Welch’s correction (**C**, **E**, **H**). Source data are available online for this figure.

Our previous work showed that mZBP1 overexpression in immortalized MEFs alone did not trigger prominent cell death without additional stimuli (Jiao et al, 2020) (Fig. EV3A). To directly compare the capacity of mZBP1 and hZBP1 in inducing cell death, we used the Dox-inducible system to overexpress FLAG-tagged hZBP1 or mZBP1 in HT-29 cells and immortalized MEFs (Fig. 2A,B,D). Consistent with prior findings, mZBP1 overexpression in both cell lines did not cause spontaneous cell death (Figs. 2C,E and EV3A). In stark contrast, hZBP1 overexpression consistently triggered robust spontaneous cell death in both tested cell lines, including that of mouse origin (Fig. 2C,E,F). Similar to hZBP1-overexpressing HT-29 cells, hZBP1 overexpression in immortalized MEFs showed apoptotic and necroptotic cell death morphological features, whereas it performed exclusively overt necroptotic phenotypes when pretreated with Em (Figs. 1C and 2F). This suggests that hZBP1 possesses an intrinsically heightened capacity to induce cell death, even across species barriers.

Given these functional distinctions, we performed co-immunoprecipitation in HT-29 cells overexpressing either hZBP1 or mZBP1 to further elucidate the differences in downstream signaling pathways nucleated by each ortholog (Fig. 2G). As expected, unlike hZBP1, mZBP1 failed to recruit key pro-death mediators—including RIPK1, RIPK3, FADD, and caspase-8—regardless of Em treatment (Fig. 2G). Collectively, these findings underscore the pivotal role of hZBP1 in nucleating a multifaceted complex that orchestrates potent cell death.

Both mZBP1 and hZBP1 share ~47% of sequence identity, each containing two conserved N-terminal Zα domains, which bind Z-NAs, and three C-terminal RIP homotypic interaction motifs (RHIMs), which mediate interactions with other RHIM-containing proteins involved in regulating ZBP1-dependent cell death and inflammatory responses. To further pinpoint the key region responsible for the species-specific function of ZBP1, we engineered chimeric ZBP1 variants by swapping the N-terminal Zα and C-terminal RHIM domains between species (termed ZBP1(m + h) and ZBP1(h + m)) and introduced them to HT-29 cells, HeLa cells, HepG2 cells, or immortalized MEFs (Figs. 2A,B,D and EV3B,D). Remarkably, ZBP1(m + h) overexpression, similar to hZBP1, induced robust cell death in all these cell lines, whereas ZBP1(h + m) overexpression failed to do so (Figs. 2C,E and EV3C,E). This strongly indicates the C-terminus of hZBP1 is the critical region that determines the spontaneous activation of overexpressed hZBP1. Furthermore, ZBP1(m + h)-induced cell death was inhibited by combined treatment with Em and necroptosis inhibitors in HT-29 cells, mirroring the mechanism observed with hZBP1 overexpression in HT-29 cells (Fig. EV3F). In contrast to the response in HT-29, when hZBP1 was expressed in immortalized MEFs, the cell death was blocked by combined treatment with Em and the RIPK3 inhibitor GSK’872, but not with Em and the RIPK1 inhibitor Nec-1s, similarly to the response observed in ZBP1(m + h) overexpression in immortalized MEFs (Figs. 2H and EV3G). This suggests that, unlike in human cells, RIPK1 kinase activity is not required for hZBP1-induced cell death in the mouse cells even when caspase activities are blocked, aligning with a previous report that mouse RIPK3 binds ZBP1 more strongly than its human counterpart (Amusan et al, 2025).

In line with these cell death findings, co-immunoprecipitation assays demonstrated that both hZBP1 and ZBP1(m + h) formed strong interactions with endogenous RIPK1 in HT-29 cells and immortalized MEFs (Fig. 2I,J). Immunofluorescence microscopy confirmed these results, revealing that both hZBP1 and ZBP1(m + h), but not mZBP1 or ZBP1(h + m), co-localized with endogenous RIPK1 in cytosolic puncta across both cell types (Figs. 2K–N and EV3H–K). These findings indicate that the C-terminal RHIM domains of hZBP1 are the primary determinants of its ability to assemble in the cytosol, interact with RIPK1, and subsequently induce apoptosis and necroptosis across different cell types and species.

### All three RHIM domains are essential for hZBP1 assembly and the ensuing cell death

**Figure 3:**
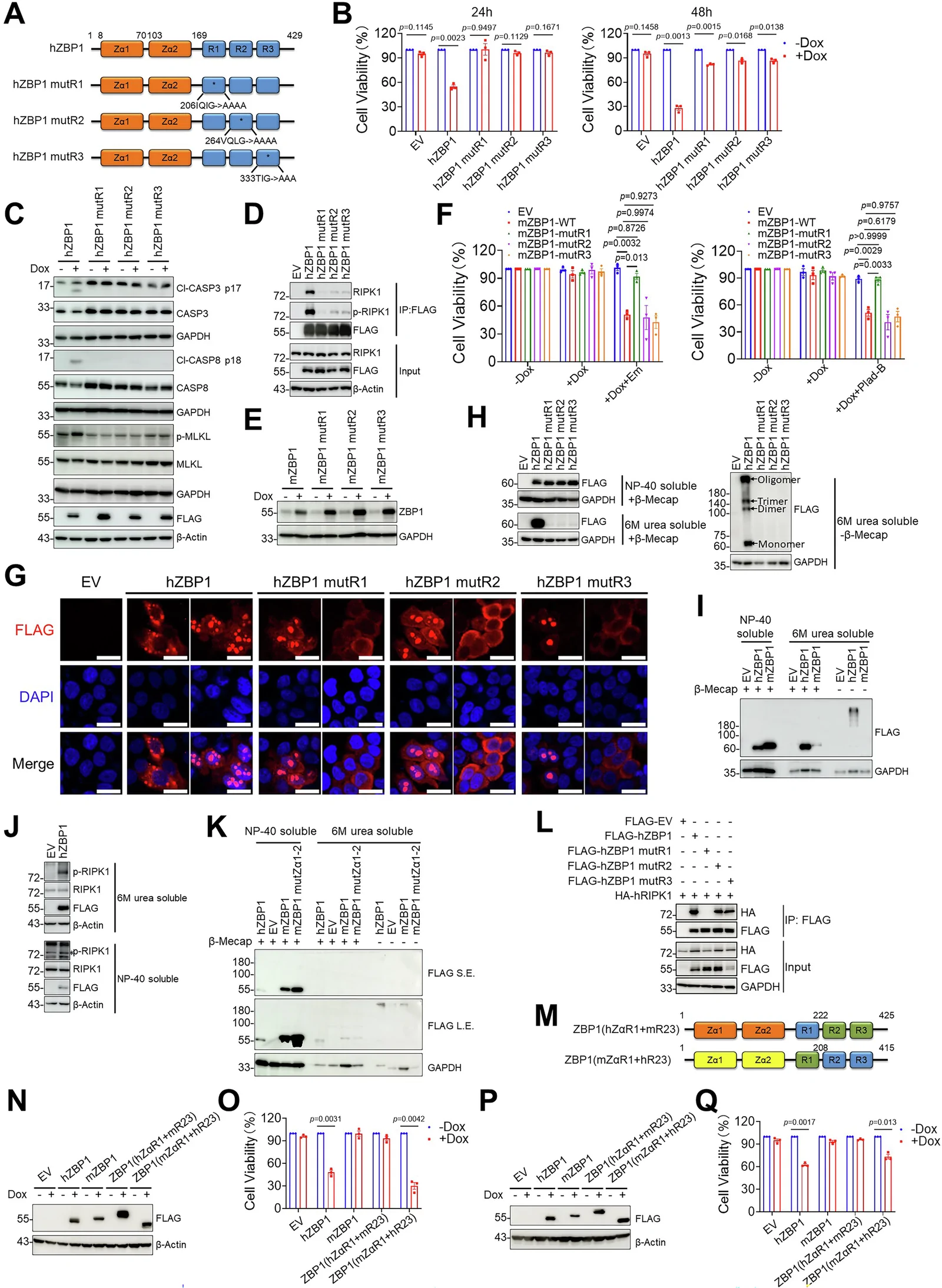
All three RHIM domains are essential for hZBP1 aggregation and the ensuing cell death. (**A**) Schematic illustration of FLAG-hZBP1 WT and FLAG-hZBP1 RHIM mutants. (**B**) Cell viability measured by neutral red uptake in HT-29 expressing Dox-inducible FLAG-EV, FLAG-hZBP1, and hZBP1 RHIM mutants stimulated with or without Dox (1 μg/mL) for 24 h (left) and 48 h (right). (**C**) Immunoblot analysis of total lysates in HT-29 expressing Dox-inducible FLAG-hZBP1 and hZBP1 RHIM mutants stimulated with or without Dox (1 μg/mL) for 10 h. (**D**) Immunoblot analysis of FLAG immunoprecipitates and total lysates (Input) from HT-29 expressing Dox-inducible FLAG-EV, FLAG-hZBP1, FLAG-hZBP1 mutR1, FLAG-hZBP1 mutR2, and FLAG-hZBP1 mutR3 stimulated with Dox (1 μg/mL) for 12 h. (**E**) Immunoblot analysis of total lysates in immortalized MEFs expressing Dox-inducible mZBP1 and mZBP1 RHIM mutants stimulated with or without Dox (1 μg/mL) for 10 h. (**F**) Cell viability measured by neutral red uptake in immortalized MEFs expressing Dox-inducible EV, mZBP1, and mZBP1 RHIM mutants stimulated with emricasan (10 μM, top) or Plad-B (10 nM, bottom) for 15 h following Dox (1 μg/mL) pretreatment for 24 h. (**G**) Representative confocal images of HT-29 expressing Dox-inducible FLAG-hZBP1, FLAG-hZBP1 mutR1, FLAG-hZBP1 mutR2, and FLAG-hZBP1 mutR3 stimulated with Dox (1 μg/mL) for 10 h. Scale bars, 20 μm. (**H**) Immunoblot analysis of NP40-soluble and 6 M urea-soluble lysates from HT-29 expressing Dox-inducible FLAG-EV, FLAG-hZBP1, FLAG-hZBP1 mutR1, FLAG-hZBP1 mutR2, and FLAG-hZBP1 mutR3 stimulated with Dox (1 μg/mL) for 10 h treated with or without β-Mecap. β-Mecap, β-mercaptoethanol. (**I**) Immunoblot analysis of NP40-soluble and 6 M urea-soluble lysates from HT-29 expressing Dox-inducible FLAG-EV, FLAG-hZBP1, and FLAG-mZBP1 stimulated with Dox (1 μg/mL) for 10 h treated with or without β-Mecap. β-Mecap, β-mercaptoethanol. (**J**) Immunoblot analysis of NP40-soluble and 6 M urea-soluble lysates from HT-29 expressing Dox-inducible FLAG-EV and FLAG-hZBP1 stimulated with Dox (1 μg/mL) for 10 h. * nonspecific band. (**K**) Immunoblot analysis of NP40-soluble and 6 M urea-soluble lysates from immortalized MEFs expressing Dox-inducible FLAG-EV, FLAG-mZBP1, and FLAG-mZBP1 mutZα1-2 stimulated with Plad-B (10 nM) for 15 h following Dox (1 μg/mL) pretreatment for 24 h. (**L**) Immunoblot analysis of FLAG immunoprecipitates and total lysates (Input) from 293T expressing HA-RIPK1 and Dox-inducible FLAG-EV, FLAG-hZBP1, FLAG-hZBP1 mutR1, mutR2, and mutR3 stimulated with Dox (1 μg/mL) for 12 h. (**M**) Schematic illustration of FLAG-tagged ZBP1(hZαR1 + mR23) and FLAG-tagged ZBP1(mZαR1 + hR23). (**N**, **P**) Immunoblot analysis of total lysates in HT-29 (**N**) and immortalized MEFs (**P**) expressing Dox-inducible FLAG-EV, hZBP1, mZBP1, ZBP1(hZαR1 + mR23), and ZBP1(mZαR1 + hR23) stimulated with or without Dox (1 μg/mL) for 10 h. (**O**, **Q**) Cell viability measured by neutral red uptake in HT-29 (**O**) and immortalized MEFs (**Q**) expressing Dox-inducible FLAG-EV, hZBP1, mZBP1, ZBP1(hZαR1 + mR23), and ZBP1(mZαR1 + hR23) stimulated with or without Dox (1 μg/mL) for 24 h. All experiments were replicated at least three times. Data with error bars are mean ± SEM. Data in panels (**B**, **F**, **O**, **Q**) are pooled and analyzed from at least three independent biological experiments. *P* values were calculated by unpaired parametric t-test with Welch’s correction (**B**, **O**, **Q**) or one-way ANOVA with Tukey’s post hoc test for multiple comparisons (**F**). Source data are available online for this figure.

RHIM domains are well-known for regulating protein-protein interactions among RHIM-containing proteins, including ZBP1, RIPK1, RIPK3, and TRIF, all of which are crucial inducers of cell death and inflammation. ZBP1 possesses three C-terminal RHIM domains. Extensive in vitro studies reveal that RHIM1, but not RHIM2 or RHIM3, is crucial for modulating mZBP1-dependent cell death induced by viral infections (Guo et al, 2018; Sridharan et al, 2017; Thapa et al, 2016). Consistently, our prior in vivo genetic model revealed that mutations in RHIM1 alone are sufficient to abolish mZBP1-mediated inflammation (Jiao et al, 2020). To further investigate whether three RHIMs of hZBP1 differentially coordinate to regulate cell death and determine its species-specific C-terminal sensitivity, we generated HT-29 cells that overexpress FLAG-tagged hZBP1 variants with individual RHIM mutations (mutR1, mutR2, or mutR3) under the Dox-inducible system (Fig. 3A). hZBP1-induced cell death was fully inhibited by mutations in any single RHIM after 24 h of Dox treatment, with substantial inhibition maintained even after 48 h (Fig. 3B). Consistent with these findings in cell death, mutations in any of the three RHIMs significantly prevented MLKL phosphorylation and blocked caspase-8 and caspase-3 cleavage, indicating inhibition of both apoptosis and necroptosis (Fig. 3C). Accordingly, RIPK1 recruitment to hZBP1 was significantly attenuated by mutations in any single RHIM (Fig. 3D). In contrast, while mZBP1 was not able to induce cell death when RHIM1 was mutated, it remained capable of inducing cell death when RHIM2 or RHIM3 was mutated in the presence of Em or Pladienolide B (Plad-B)—compounds known to trigger mZBP1-dependent cell death (He et al, 2025; Jiao et al, 2020) (Fig. 3E,F). Collectively, these results demonstrate that hZBP1 and mZBP1 RHIMs function through distinct regulatory mechanisms.

Notably, mutations in any of the three RHIM domains completely abolished hZBP1 cytoplasmic puncta while preserving the larger nuclear condensates (Fig. 3G). This suggests that RHIMs are essential for hZBP1’s formation of solid-state assemblies. To differentiate between soluble and condensed proteins, we used NP40 lysis buffer and 6 M urea buffer to extract soluble and insoluble fractions, respectively. While wild-type hZBP1 and its RHIM mutants showed similar protein levels in NP40-soluble fractions, only wild-type hZBP1 exhibited a strong signal in the NP40-insoluble fraction when treated with β-mercaptoethanol (Fig. 3H). Importantly, under non-reducing conditions, 6 M urea solutions allowed detection of wild-type hZBP1 as monomers, dimers, trimers, and higher-order oligomers (Fig. 3H). These multimeric forms were absent in all three hZBP1 RHIM mutants and in mZBP1-overexpressing HT-29 cells (Fig. 3H,I), confirming that hZBP1 overexpression in HT-29 cells leads to RHIM-dependent self-oligomerization. Additionally, activated RIPK1 was engaged with these hZBP1 oligomers (Fig. 3J), consistent with the observed co-localization of RIPK1 with hZBP1 cytoplasmic puncta (Fig. 1H). Collectively, these results indicate that all three hZBP1 RHIMs are involved in its cytoplasmic assemblies. These differential RHIM requirements for overexpression-dependent spontaneous cell death may underlie the distinct cell death-inducing capabilities of hZBP1 and mZBP1. Indeed, treating mZBP1-overexpressing immortalized MEFs with Plad-B induced the oligomerization of wild-type mZBP1, but not Zα domains-mutated mZBP1 (Fig. 3K), supporting the notion that unlike hZBP1, mZBP1 requires an additional Z-NAs trigger to form higher-order assemblies.

Surprisingly, when we overexpressed both hZBP1 and RIPK1 in 293T cells, we found that mutations in RHIM1, but not RHIM2 or RHIM3, completely disrupted the interaction between hZBP1 and RIPK1 (Fig. 3L). This finding aligns with previous reports and suggests that RHIM1 of hZBP1 is the primary mediator of its interaction with RIPK1 (Jiao et al, 2020; Thapa et al, 2016). Therefore, we hypothesized that the C-terminal region of hZBP1, which contains RHIM2 and RHIM3, determines cell death-inducing capability in overexpressing cells. To test this, we engineered chimeric ZBP1 variants by swapping the N-terminal Zα plus RHIM1 and the C-terminal regions (containing RHIM2 and RHIM3) between human and mouse ZBP1 (Fig. 3M). We named these variants (termed ZBP1(mZαR1 + hR23) and ZBP1(hZαR1 + mR23)) and introduced them into HT-29 cells or immortalized MEFs (Fig. 3N,P). As expected, overexpression of ZBP1(mZαR1 + hR23) led to robust cell death in both cell lines, while ZBP1(hZαR1 + mR23) overexpression did not (Fig. 3O,Q). This strongly suggests the C-terminus of hZBP1, specifically the region containing RHIM2 and RHIM3, determines cell death by promoting the interaction of RHIM1 with downstream RIPK1.

### Zα domains contribute to hZBP1-mediated cell death and inflammation

**Figure 4:**
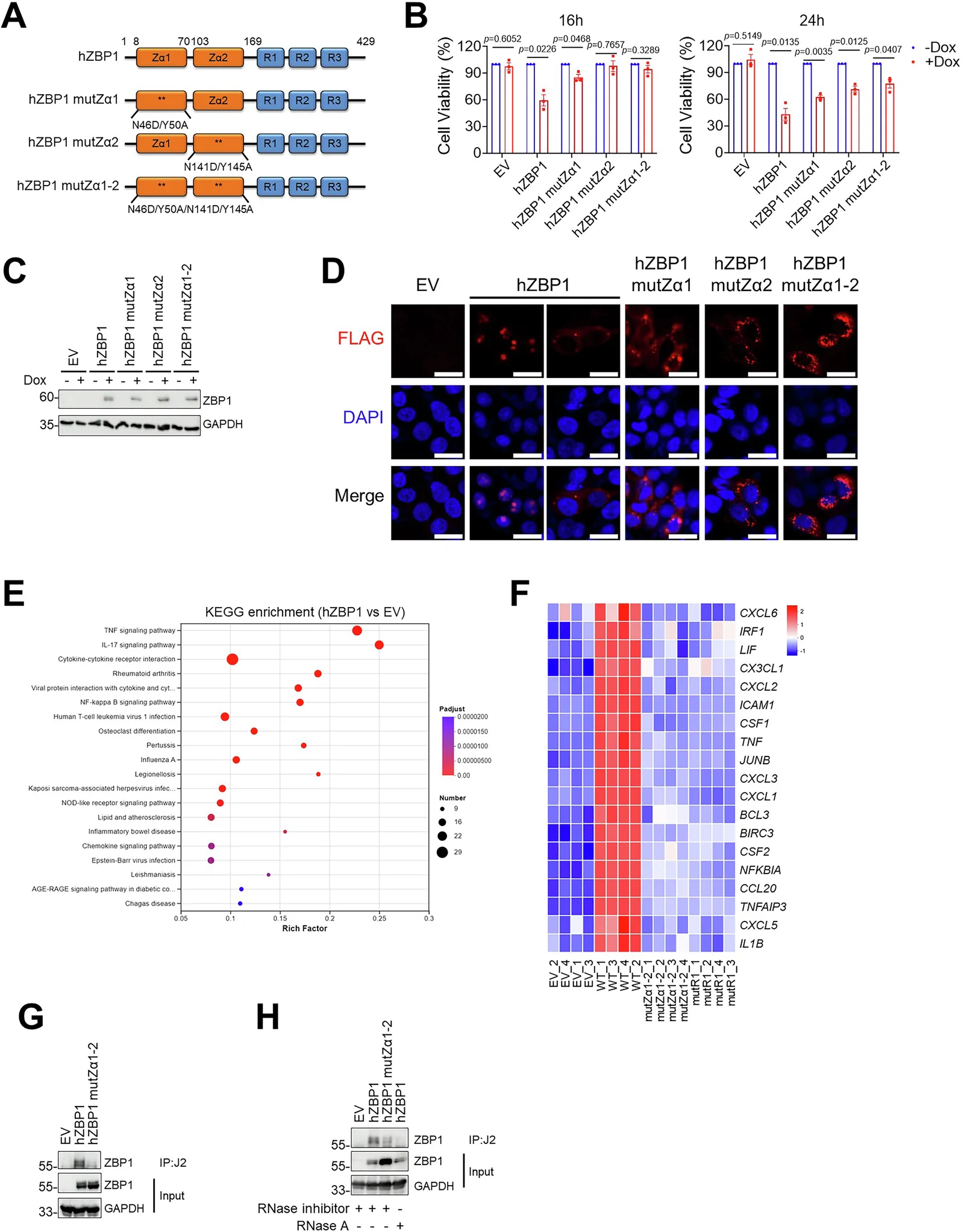
Zα domains contribute to hZBP1-mediated cell death and inflammation. (**A**) Schematic illustration of FLAG-hZBP1 WT and FLAG-hZBP1 Zα mutants. (**B**) Cell viability measured by neutral red uptake in HT-29 expressing Dox-inducible FLAG-EV, FLAG-hZBP1, and hZBP1 Zα mutants stimulated with or without Dox (1 μg/mL) for 16 h (left) and 24 h (right). (**C**) Immunoblot analysis of total lysates in HT-29 expressing Dox-inducible FLAG-EV, FLAG-hZBP1, and hZBP1 Zα mutants stimulated with or without Dox (1 μg/mL) for 10 h. (**D**) Representative confocal images of HT-29 expressing Dox-inducible FLAG-hZBP1, FLAG-hZBP1 mutZα1, FLAG-hZBP1 mutZα2, and FLAG-hZBP1 mutZα1-2 stimulated with Dox (1 μg/mL) for 10 h. Scale bars, 20 μm. (**E**) Dot plot of the KEGG enrichment analysis for differentially expressed genes (DEGs) compared between HT-29 expressing Dox-inducible FLAG-EV and FLAG-hZBP1. (**F**) Heatmap analysis of DEGs enriched in the TNF signaling pathway compared between HT-29 expressing Dox-inducible FLAG-EV, FLAG-hZBP1 WT, FLAG-hZBP1 mutZα1-2, and FLAG-hZBP1 mutR1. (**G**) Immunoblot analysis of J2 immunoprecipitates and total lysates (Input) from HT-29 expressing Dox-inducible FLAG-EV, FLAG-hZBP1, and FLAG-hZBP1 mutZα1-2 stimulated with Dox (1 μg/mL) for 12 h. (**H**) Immunoblot analysis of J2 immunoprecipitates and total lysates (Input) from HT-29 expressing Dox-inducible FLAG-EV, FLAG-hZBP1, and FLAG-hZBP1 mutZα1-2 stimulated with Dox (1 μg/mL) for 12 h treated with either RNase inhibitor or RNase A. All experiments were replicated at least three times. Data with error bars are mean ± SEM. Data in panel (**B**) are pooled and analyzed from at least three independent biological experiments. *P* values were calculated by unpaired parametric *t*-test with Welch’s correction (**B**). Source data are available online for this figure.

Studies have shown that mZBP1 senses Z-RNA, Z-DNA, and Z-RNA:DNA via its Zα domains to regulate cell death, inflammation, antiviral responses, and anti-tumor responses (He et al, 2025; Zhang et al, 2020; Zhang et al, 2022a). To investigate the contributions of Zα domains to hZBP1’s functions in inducing cell death and inflammation, we introduced various mutations into the Zα domains of hZBP1, targeting key nucleic acid recognition sites in Zα1, Zα2, or both (Fig. 4A). By overexpressing these hZBP1 Zα mutants in HT-29 cells, we discovered that both Zα domains were involved in driving cell death, as either single or double Zα domain mutations inhibited hZBP1-mediated cell death at 16 h post-Dox treatment (Fig. 4B,C). However, despite being less potent than wild-type hZBP1, these single or double Zα mutations still resulted in robust cell death 24 h post-Dox induction (Fig. 4B,C), indicating that sensing of endogenous Z-NAs by Zα domains is not absolutely required for hZBP1 overexpression-driven cell death. Interestingly, while RHIM-mutated hZBP1 formed puncta only in the nucleus (Fig. 3G), single or double Zα mutations resulted in exclusively cytoplasmic puncta of hZBP1 in HT-29 cells (Fig. 4D). This is in line with the dispensable role of hZBP1 Zα domains in triggering cell death (Fig. 4B).

**Figure EV4:**
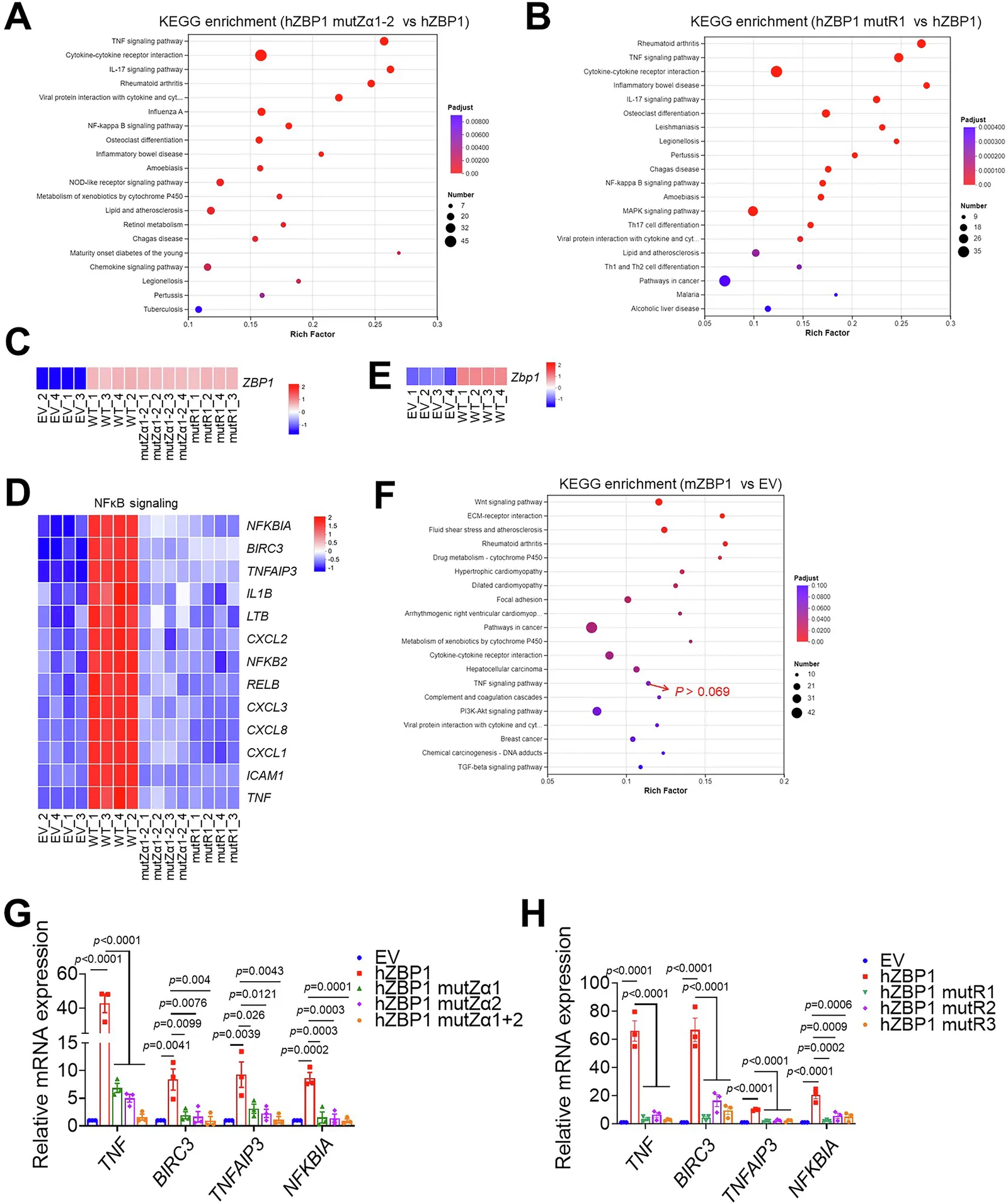
Zα and RHIM domains contribute to hZBP1-mediated inflammation. (**A**, **B**) Dot plot of the KEGG enrichment analysis for DEGs compared between HT-29 expressing Dox-inducible FLAG-hZBP1 mutZα1-2 (**A**) and FLAG-hZBP1 mutR1 (**B**) with FLAG-hZBP1 WT. (**C**) Heatmap analysis of ZBP1 expression compared between HT-29 expressing Dox-inducible FLAG-EV, FLAG-hZBP1, FLAG-hZBP1 mutZα1-2, and FLAG-hZBP1 mutR1. (**D**) Heatmap analysis of DEGs enriched in NFκB signaling compared between HT-29 expressing Dox-inducible FLAG-EV, FLAG-hZBP1 WT, FLAG-hZBP1 mutZα1-2, and FLAG-hZBP1 mutR1. (**E**) Heatmap analysis of *Zbp1* expression compared between immortalized MEFs expressing Dox-inducible FLAG-EV and FLAG-mZBP1. (**F**) Dot plot of the KEGG enrichment analysis for DEGs compared between immortalized MEFs expressing Dox-inducible FLAG-EV and FLAG-mZBP1. (**G**, **H**) qRT-PCR analysis of mRNA expression of the indicated genes in HT-29 expressing Dox-inducible FLAG-EV, FLAG-hZBP1 WT, FLAG-hZBP1 Zα mutants (**G**), and FLAG-hZBP1 RHIM mutants (**H**) stimulated with or without Dox (1 μg/mL) for 10 h. All experiments were replicated at least three times. Data with error bars are mean ± SEM. Data in panels (**G**, **H**) are pooled and analyzed from at least three independent biological experiments. *P* values were calculated by one-way ANOVA with Tukey’s post hoc test for multiple comparisons (**G**, **H**). Source data are available online for this figure.

Next, we performed RNA-sequencing analysis to examine cellular processes involved in hZBP1-triggered cell death. The KEGG annotation and enrichment analysis revealed that wild-type hZBP1 overexpression, unlike mZBP1, triggered the upregulation of numerous genes associated with NF-κB signaling, TNF signaling, and inflammatory bowel disease pathways (Figs. 4E and EV4A–F). Further analysis indicated that Zα mutations significantly attenuated the levels of many pro-inflammatory genes regulated by the NF-κB pathway (Figs. 4F and EV4A,D). We selected several representative genes to confirm the RNA-Seq data by performing qRT-PCR. The results demonstrated that mutating Zα1 or Zα2 alone significantly suppressed pro-inflammatory gene expression, while mutations in both Zα domains almost completely blocked it (Fig. EV4G). This suggests that sensing endogenous Z-NAs is crucial for hZBP1’s role in regulating pro-inflammatory gene expression. To assess whether hZBP1 binds cellular double-stranded (ds)RNA via its Zα domains similarly to mZBP1, we immunoprecipitated dsRNA from HT-29 cells expressing wild-type hZBP1 or hZBP1 mutZα1-2 using the dsRNA-specific J2 antibody, followed by immunoblotting with anti-ZBP1 antibody (Fig. 4G,H). We found that wild-type hZBP1, but not hZBP1 mutZα1-2, was co-immunoprecipitated with dsRNA, indicating hZBP1 binds to dsRNA in a Zα-dependent manner. Furthermore, RNase A treatment eliminated the enrichment of wild-type hZBP1 in the J2-enriched immunoprecipitates (Fig. 4H), confirming that dsRNA is indeed an endogenous ligand of hZBP1 to promote NF-kB-dependent pro-inflammatory genes. Interestingly, further analysis showed that RHIM1 mutation also significantly attenuated the levels of numerous pro-inflammatory genes involved in the NF-κB pathway (Figs. 4F and EV4B,D,H). These results are consistent with a prior finding that hZBP1 regulates pro-inflammatory gene expression via both Z-NAs sensing and its RHIM1 function (Peng et al, 2022). Taken together, our findings indicate that endogenous Z-RNA sensing contributes to hZBP1-induced cell death and is required for hZBP1-induced pro-inflammatory gene expression.

### Short isoform of hZBP1 triggers cell death and inflammation

**Figure EV5:**
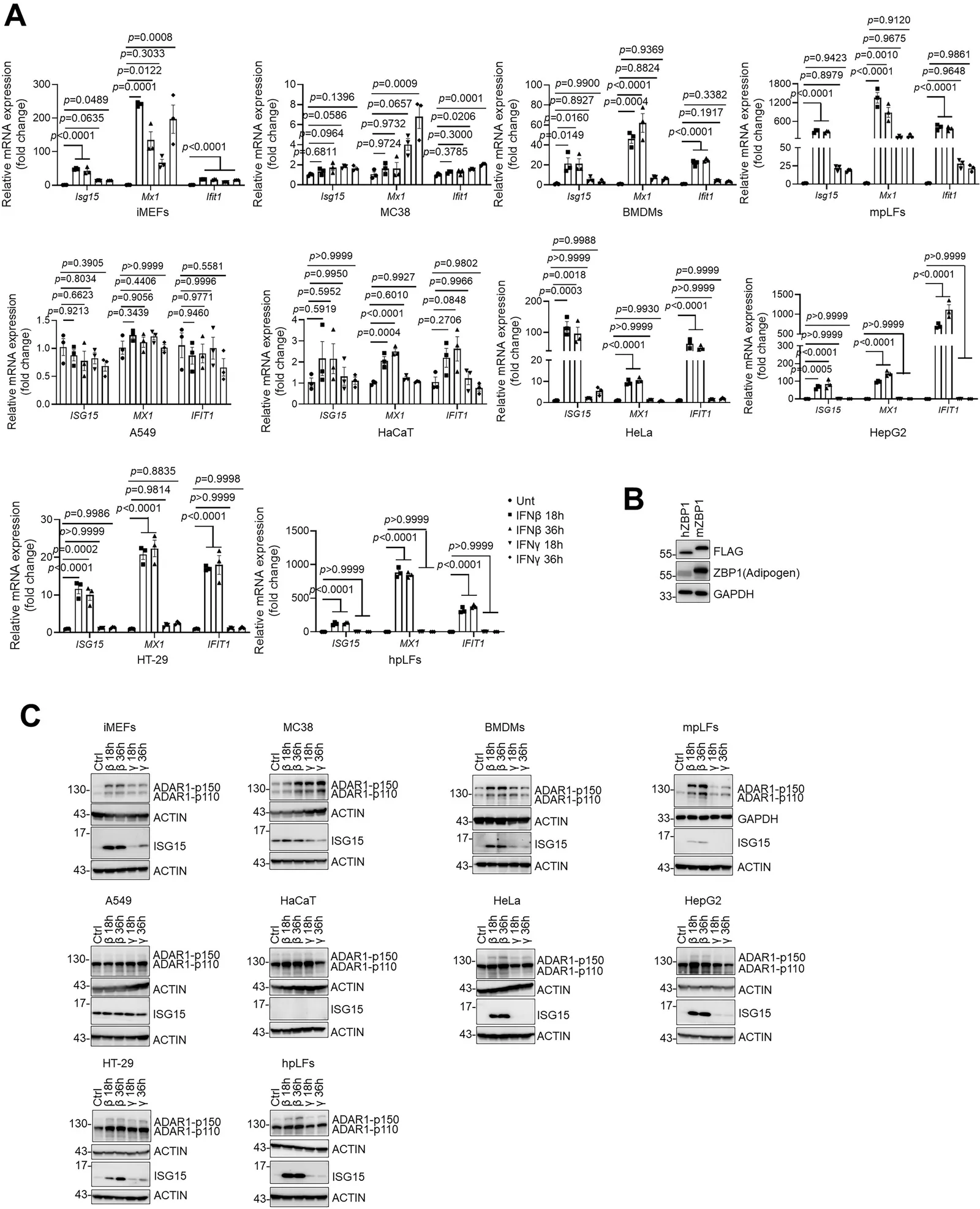
ISG expression in human and mouse cells upon IFN treatment. (**A**) qRT-PCR analysis of mRNA expression of the indicated ISGs in different human and murine cells stimulated with or without IFNβ (20 ng/mL) or IFNγ (20 ng/mL) for 18 or 36 h. (**B**) Immunoblot analysis of total lysates in ZBP1 KO HeLa cells transfected with FLAG-hZBP1 and FLAG-mZBP1, and then blotted for FLAG and ZBP1 (Adipogen). (**C**) Immunoblot analysis of total lysates in different human and murine cells stimulated with or without IFNβ (20 ng/mL) or IFNγ (20 ng/mL) for 18 or 36 h, and then blotted for ADAR1-p150 and ISG15. All experiments were replicated at least three times. Data with error bars are mean ± SEM. Data from panel (**A**) are pooled and analyzed from at least three independent biological experiments. *P* values were calculated by one-way ANOVA with Tukey’s post hoc test for multiple comparisons (**A**). Source data are available online for this figure.

**Figure 5:**
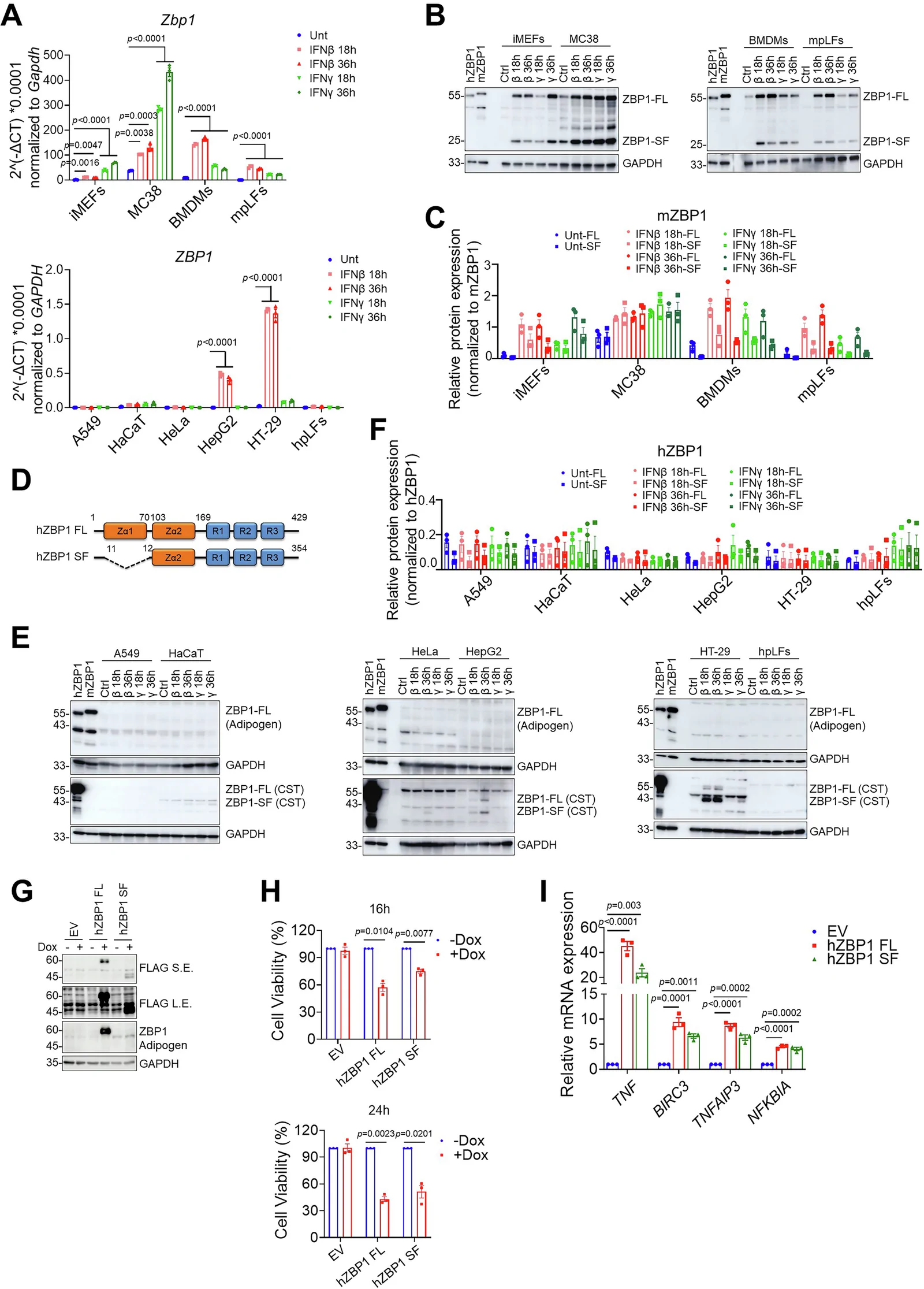
Short isoform of hZBP1 triggers cell death and inflammation. (**A**) qRT-PCR analysis of relative mRNA expression of *Zbp1* in murine cells (top) and *ZBP1* in human cells (bottom) normalized to *GAPDH*. Murine cells were stimulated with or without mIFNβ (20 ng/mL) or mIFNγ (20 ng/mL) for 18 or 36 h. Human cells were stimulated with or without hIFNβ (20 ng/mL) or hIFNγ (20 ng/mL) for 18 or 36 h. (**B**) Immunoblot analysis of total lysates in murine cells stimulated with or without mIFNβ (20 ng/mL) or mIFNγ (20 ng/mL) for 18 or 36 h. Total lysates in ZBP1 KO HeLa cells overexpressing FLAG-tagged hZBP1 or mZBP1 were loaded as positive controls. (**C**) Relative quantification of mZBP1 expression by gray value in murine cells stimulated with or without mIFNβ (20 ng/mL) or mIFNγ (20 ng/mL) for 18 or 36 h normalized to ZBP1 KO HeLa cells overexpressing FLAG-tagged mZBP1. (**D**) Schematic illustration of FLAG-tagged hZBP1-FL and FLAG-tagged hZBP1-SF. (**E**) Immunoblot analysis of total lysates in human cells stimulated with or without hIFNβ (20 ng/mL) or hIFNγ (20 ng/mL) for 18 or 36 h. Total lysates in ZBP1 KO HeLa cells overexpressing FLAG-tagged hZBP1 or mZBP1 were loaded as positive controls. (**F**) Relative quantification of hZBP1 expression by gray value in human cells stimulated with or without hIFNβ (20 ng/mL) or hIFNγ (20 ng/mL) for 18 or 36 h, normalized to ZBP1 KO HeLa cells overexpressing FLAG-tagged hZBP1. (**G**) Immunoblot analysis of total lysates in HT-29 expressing Dox-inducible FLAG-EV, FLAG-hZBP1-FL, and hZBP1-SF stimulated with or without Dox (1 μg/mL) for 10 h. (**H**) Cell viability measured by neutral red uptake in HT-29 expressing Dox-inducible FLAG-EV, FLAG-hZBP1-FL, and hZBP1-SF stimulated with or without Dox (1 μg/mL) for 16 h (top) and 24 h (bottom). (**I**) qRT-PCR analysis of mRNA expression of the indicated genes in HT-29 expressing Dox-inducible FLAG-EV, FLAG-hZBP1-FL, and FLAG-hZBP1-SF stimulated with or without Dox (1 μg/mL) for 10 h. All experiments were replicated at least three times. Data with error bars are mean ± SEM. Data in panels (**A**, **H**, **I**) are pooled and analyzed from at least three independent biological experiments. *P* values were calculated by unpaired parametric *t*-test with Welch’s correction (**H**) or one-way ANOVA with Tukey’s post hoc test for multiple comparisons (**A**, **I**). Source data are available online for this figure.

Our research indicates that hZBP1 is a more potent inducer of cell death than its mouse counterpart, mZBP1, under overexpression conditions. This suggests that in human cells, hZBP1 expression must be tightly regulated to prevent unintended cell death. To systematically compare endogenous ZBP1 expression across species, we analyzed a panel of human-derived cell lines (HT-29, A549, HeLa, HepG2, and HaCaT) and primary lung fibroblasts (pLFs), as well as murine-derived MC38, immortalized MEFs, bone marrow-derived macrophages (BMDMs), and pLFs. Consistent with our hypothesis, although IFNβ/IFNγ treatment induced the expression of representative interferon-stimulated genes (ISGs) to varying degrees across all tested cell lines (Fig. EV5A), both basal and IFNβ/IFNγ-stimulated *hZBP1* mRNA levels (normalized to *GAPDH*) were substantially lower in human cells than their *mZbp1* counterparts in mouse cells (Fig. EV5A). Notably, *hZBP1* transcripts were hardly detectable in A549, HaCaT, HeLa, and human pLFs (Fig. 5A). At the protein level, a validated anti-mouse ZBP1 antibody (Adipogen) exhibited cross-reactivity with both exogenous FLAG-hZBP1 and FLAG-mZBP1 (Fig. 5B,E). Notably, when the loading amounts were normalized based on FLAG immunoblotting (Fig. EV5B), this ZBP1 antibody possessed higher affinity for mZBP1 than for hZBP1 (Figs. 5B,E and EV5B). Leveraging this cross-reactive property, we utilized the antibody to evaluate the relative ZBP1 protein induction across human and murine cell lines in response to IFNβ/IFNγ stimulation. Although mZBP1 was readily detectable in all tested murine cells after IFNβ/IFNγ treatment, with the full length mZBP1 being a more prominent isoform (Fig. 5B), hZBP1 was undetectable in all tested human cells (Fig. 5E). While A549 and HaCaT cells remained largely unresponsive to the indicated IFNβ/IFNγ stimulation (Fig. EV5A,C), other human cell lines exhibited varying degrees of IFN pathway activation, as evidenced by the induction of classical ISGs at both the mRNA (*ISG15*, *MX1*, and *IFIT1*) and protein (ISG15 and ADAR1-p150) levels (Fig. EV5A,C). Importantly, quantification of immunoblotting bands confirmed that hZBP1 was expressed at markedly lower levels than its murine counterpart following IFNβ/IFNγ treatment (Fig. 5C,F). Furthermore, a comparative analysis of species-matched cell types—including colon cancer lines (murine MC38 vs. human HT-29) and primary lung fibroblasts (pLFs)—consistently demonstrated that hZBP1 is expressed at significantly diminished levels relative to the mZBP1 ortholog (Fig. 5B,E). Such striking differences underscore the possibility that hZBP1 expression is precisely orchestrated via yet-to-be-identified mechanisms, presumably to safeguard against accidental cell death.

A short hZBP1 isoform lacking the Zα1 domain (hZBP1-SF) has been annotated (Rothenburg et al, 2002) (Fig. 5D). Utilizing an hZBP1-specific antibody from CST, we successfully detected both the full-length (hZBP1-FL) and short (hZBP1-SF) isoforms in HT-29 cells following IFNβ priming (Fig. 5D,E). While these isoforms exhibited detectable but weaker expression in HepG2 and HeLa cells, they remained entirely absent in A549, HaCaT, and human pLFs (Fig. 5E). Notably, the hZBP1-FL isoform was present at a substantially lower level than hZBP1-SF (Fig. 5E). To characterize the function of hZBP1-SF in inducing cell death and inflammation, we generated doxycycline (Dox)-inducible hZBP1-SF-overexpressing HT-29 cells and HepG2 cells (Figs. 5G and EV1C). We observed that hZBP1-SF was able to induce robust cell death in HT-29 cells and HepG2 cells post-Dox treatment, albeit weaker than that induced by hZBP1-FL overexpression (Figs. 5H and EV1D). Consistently, hZBP1-SF overexpression induced pro-inflammatory gene expression to a lesser extent compared to hZBP1-FL (Fig. 5I). These data indicate both isoforms of hZBP1 expressed in human cells are potent inducers of cell death and inflammation.

### RIPK1 is required for endogenous ZBP1-mediated apoptosis in human cells

**Figure 6:**
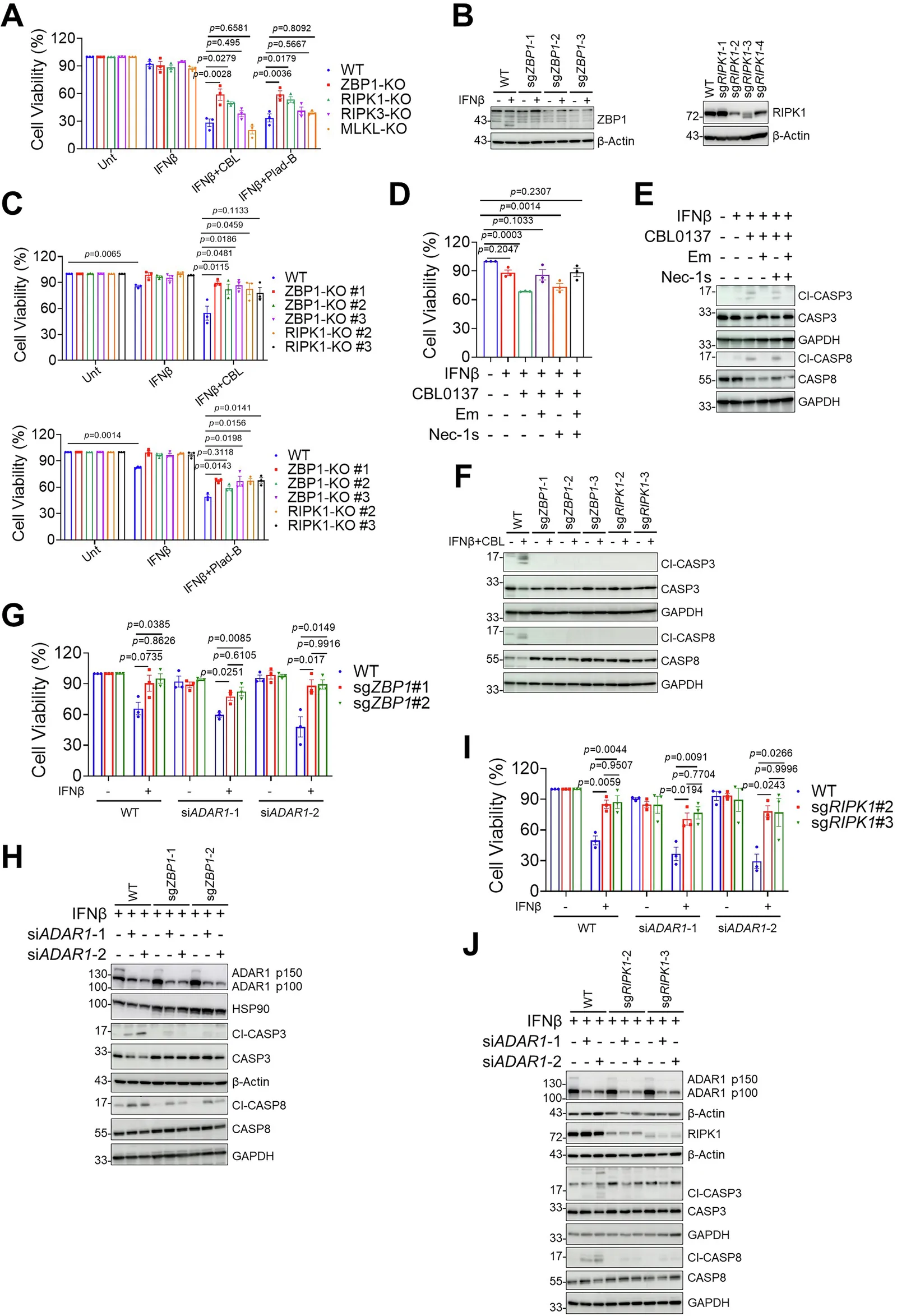
RIPK1 is required for endogenous ZBP1-mediated apoptosis in human cells. (**A**) Cell viability measured by neutral red uptake in HT-29 WT, ZBP1 KO, RIPK1 KO, RIPK3 KO, and MLKL KO stimulated with combinations of hIFNβ (100 ng/mL) (24 h pretreatment) and CBL (2 μM) or Plad-B (15 nM) for 15 h. (**B**) Left, immunoblot analysis of total lysates in HeLa WT and three independent ZBP1 KO cells stimulated with or without hIFNβ (100 ng/mL) for 48 h. Right, immunoblot analysis of total lysates in HeLa WT and four independent RIPK1 KO cells. (**C**) Top, cell viability measured by neutral red uptake in WT, ZBP1 KO, and RIPK1 KO HeLa stimulated with combinations of hIFNβ (100 ng/mL) (24 h pretreatment) and CBL (1 μM) for 15 h. Bottom, cell viability measured by neutral red uptake in WT, ZBP1 KO, and RIPK1 KO HeLa stimulated with combinations of hIFNβ (100 ng/mL) (24 h pretreatment) and Plad-B (15 nM) for 21 h. (**D**) Cell viability measured by neutral red uptake in HeLa stimulated with combinations of hIFNβ (100 ng/mL) (24 h pretreatment), CBL (1 μM), emricasan (5 μM), and Nec-1s (10 μM) for 15 h. (**E**) Immunoblot analysis of total lysates from HeLa stimulated with combinations of hIFNβ (100 ng/mL) (24 h pretreatment), CBL (1 μM), emricasan (5 μM), and Nec-1s (10 μM) for 15 h. (**F**) Immunoblot analysis of total lysates from WT, ZBP1 KO, and RIPK1 KO HeLa stimulated with combinations of hIFNβ (100 ng/mL) (24 h pretreatment) and CBL (1 μM) for 15 h. (**G**) Cell viability measured by neutral red uptake in WT and ZBP1 KO HeLa pretreated with hIFNβ (100 ng/mL) for 24 h and transfected with si*ADAR1* for another 36 h. (**H**) Immunoblot analysis of total lysates from WT and ZBP1 KO HeLa pretreated with hIFNβ (100 ng/mL) for 24 h and transfected with si*ADAR1* for another 36 h. (**I**) Cell viability measured by neutral red uptake in WT and RIPK1 KO HeLa pretreated with hIFNβ (100 ng/mL) for 24 h and transfected with si*ADAR1* for another 36 h. (**J**) Immunoblot analysis of total lysates from WT and RIPK1 KO HeLa pretreated with hIFNβ (100 ng/mL) for 24 h and transfected with si*ADAR1* for another 36 h. All experiments were replicated at least three times. Data with error bars are mean ± SEM. Data in panels (**A**, **C**, **D**, **G**, **I**) are pooled and analyzed from at least three independent biological experiments. *P* values were calculated by one-way ANOVA with Tukey’s post hoc test for multiple comparisons (**A**, **C**, **D**, **G**, **I**). Source data are available online for this figure.

To determine whether upregulation of endogenous hZBP1 expression induces cell death in human cells, we stimulated HT-29 cells with human IFNβ. Although IFNβ upregulated endogenous hZBP1 expression, this induction was insufficient to trigger significant cell death, as by hZBP1 overexpression (Fig. 6A). Previous studies showed that small molecules such as CBL0137 (CBL) and Plad-B can trigger mZBP1-mediated cell death by accumulation of specific ligands for ZBP1 (He et al, 2025; Zhang et al, 2022b). To examine whether this applied to human cells, IFNβ-primed HT-29 cells expressing endogenous hZBP1 were stimulated with CBL or Plad-B. Both CBL and Plad-B induced potent, ZBP1-dependent cell death, and the death was largely declined by RIPK1 deficiency (Fig. 6A). In contrast, deficiency of RIPK3 or MLKL showed modest or no inhibitory effect, suggesting that hZBP1 activation by CBL or Plad-B can trigger RIPK1-dependent apoptosis in these necroptosis-deficient HT-29 cells (Fig. 6A).

We also assess the role of RIPK1 in transducing ZBP1 signaling in HeLa cells lacking RIPK3 expression (Koo et al, 2015), precluding RIPK3-MLKL-mediated necroptosis downstream of ZBP1. Notably, unlike the requirements observed in HT-29 cells, IFNβ treatment alone was sufficient to trigger significant ZBP1- and RIPK1-dependent cell death in HeLa cells (Fig. 6C). Furthermore, following IFNβ pretreatment, both CBL and Plad-B induced a more significant cell death along with the proteolytic cleavage of caspase-8 and caspase-3, which were abolished in ZBP1 KO and RIPK1 KO cells (Fig. 6B,C,F). Moreover, Em alone, but not Nec-1s, completely prevented CBL-induced cell death and the activation of caspase-8 and caspase-3 (Fig. 6D,E). Collectively, these data demonstrate that RIPK1 plays a critical role in triggering apoptosis downstream of ZBP1 activation in human cells, irrespective of its kinase activity.

ADAR1 deficiency was also reported to trigger ZBP1-mediated cell death by inducing aberrant Z-RNA accumulation (Zhang et al, 2022a). To further validate this, we transfected siRNA targeting *ADAR1* in HeLa cells. As expected, IFNβ pretreatment followed by ADAR1 knockdown induced significant cell death, and the death was rescued in ZBP1 KO cells (Fig. 6G,H). Consistently, apoptosis activation marked by caspase-8 and caspase-3 cleavage was detected upon ADAR1 knockdown, and these were substantially attenuated in ZBP1 KO cells (Fig. 6H). Notably, si*ADAR1*-induced apoptosis was similarly abolished in RIPK1 KO cells (Fig. 6I,J). Collectively, these results demonstrate a fundamental role for the ZBP1-RIPK1 axis in mediating apoptosis upon nuclear Z-NA accumulation across human cell lines, aligning with findings from overexpression systems.

## Discussion

ZBP1 is increasingly recognized for its crucial role in the initiation of inflammatory cell death as a viral defense strategy. Beyond viral sensing, its Zα domains also detect endogenous Z-nucleic acids, thereby modulating pathological inflammation. A variety of in vivo mouse studies have confirmed the importance of ZBP1 in driving skin inflammation in RIPK1^E-KO^ mice, intestinal inflammation in FADD^IEC-KO^ mice, and autoinflammatory diseases in *Adar1*^mutZα/−^ mice (Devos et al, 2020; de Reuver et al, 2022; Hubbard et al, 2022; Jiao et al, 2020; Jiao et al, 2022). Despite these murine insights, very few systematic studies have explored the mechanisms of hZBP1 activation. Nevertheless, human genetic evidence points to a potential connection of hZBP1 in human disease: *RIPK1* mutant patients have apoptotic epidermal cells and activated Caspase-3 in their colonic tissue; *CASPASE-8* mutant patients develop perianal diseases; and *ADAR1* mutant patients suffer from autoinflammatory diseases with intracranial calcification (Cuchet-Lourenco et al, 2018; Li et al, 2019; Rice et al, 2012; Uchiyama et al, 2019).

Our research demonstrates that hZBP1, in stark contrast to its mouse counterpart, can directly recruit RIPK1 independently of RIPK3 to induce both FADD-caspase-8-dependent apoptosis and RIPK3-MLKL-dependent necroptosis while also triggering pro-inflammatory responses. This signaling activation is facilitated by both Zα domain-mediated recognition of Z-NAs and RHIM domains-mediated oligomerization and cytoplasmic solid-like assembly. Interestingly, disrupting the endogenous Z-NAs sensing only delayed, but did not completely prevent, hZBP1 overexpression-induced cell death. However, this disruption of Z-NA sensing almost entirely blocked hZBP1-induced NF-κB activation. This finding highlights that Z-NA sensing, presumably of Z-RNA, is a crucial step for hZBP1 to activate inflammation, and this process is likely independent of the cell death pathway. In contrast to mZBP1-activated signaling, RIPK1 plays a critical and universal role in transducing these downstream signaling cascades in response to hZBP1 activation across various human cell lines independently of RIPK3. This was evident both in our Dox-inducible overexpression system and in contexts of nuclear Z-NA accumulation induced by drug treatment or ADAR1 deficiency. This notion is consistent with other findings, which show that RIPK1 mediates ZBP1-driven necroptosis during HSV-1(ICP6*mut*RHIM) infection and promotes cell death-independent inflammatory signaling through the formation of a pro-inflammatory complex where ZBP1 and RIPK1 are ubiquitinated (Amusan et al, 2025; Peng et al, 2022). Overall, our work provides strong evidence that the hZBP1-RIPK1 axis is a potential therapeutic target in human inflammatory diseases. Interestingly, while we observed significant RIPK1 phosphorylation in hZBP1-overexpressing cells, RIPK1 kinase activity is not required for hZBP1-induced cell death, especially redundant for hZBP1-induced apoptosis. However, hZBP1-induced necroptosis, with or without caspase inhibition, depends on RIPK1 kinase activity, consistent with the mechanisms underlying mZBP1-induced cell death (Maelfait et al, 2017; Schwarzer et al, 2020).

Interestingly, we observed that Em sensitizes FADD KO cells to ZBP1-induced cell death, suggesting the presence of residual caspase-8 activity that restrains a small portion of necroptosis independently of FADD. While the exact mechanism governing FADD-independent caspase-8 activation remains to be elucidated, this notion is supported by genetic precedents demonstrating that mice with intestinal epithelial cell-specific FADD deletion develop ileitis in a caspase-8-dependent manner when MLKL is knocked out (Schwarzer et al, 2020).

Our research shows that, unlike mZBP1, hZBP1 overexpression is sufficient to trigger cell death in both mouse and human cells without needing additional stimuli. At the same time, we found that hZBP1, but not mZBP1, promotes the expression of NF-κB-targeted genes, which is consistent with a previous study (Peng et al, 2022). These results demonstrate that hZBP1 has a more potent capacity to induce both cell death and pro-inflammatory responses than mZBP1. By creating chimeric ZBP1 constructs, we mapped this enhanced capacity of hZBP1 to its C-terminal region, specifically the region containing RHIM2 and RHIM3. We propose that the C-terminal region acts as a regulatory element working with RHIM1 to recruit RIPK1 and initiate downstream signaling. Consistent with this model, mutation of any single RHIM in hZBP1 abolished cell death induction, which is in stark contrast to mZBP1, where only RHIM1 mutation blocks cell death (Guo et al, 2018; Sridharan et al, 2017; Thapa et al, 2016).

It is worth mentioning that wild-type hZBP1 forms heterogeneous condensates in both nuclear and cytoplasmic compartments. In contrast, Zα-mutant hZBP1 exclusively assembles into solid-like aggregates in the cytoplasm, and RHIM-mutant hZBP1 (any single RHIM mutated) generates liquid-like condensates specifically in the nucleus. These findings suggest that Zα domains may initiate Z-NA-driven liquid-liquid phase separation (LLPS), likely enabling hZBP1 activation, whereas RHIM domains could orchestrate solid-phase condensation to facilitate RIPK1 recruitment and subsequent cell death execution. Therefore, Zα-mutant hZBP1 could form a solid-state assembly in the cytoplasm by its RHIMs, bypassing Z-NA sensing to initiate downstream signaling.

We demonstrated that the expression threshold for hZBP1 to trigger significant cell death is much lower than for mZBP1. In this regard, hZBP1 expression must be tightly regulated to prevent excessive, unwanted cell death. Supporting this, we observed that hZBP1 levels in human cells remain markedly lower than those of mZBP1 in murine cells, even following IFN priming. The mechanisms maintaining low hZBP1 levels remain unknown, but one plausible explanation is the existence of a specific transcriptional regulatory mechanism in human cells, such as a less accessible promoter region compared to mZBP1, given that *hZBP1* mRNA levels are consistently diminished relative to *mZbp1* regardless of IFN stimulation.

Collectively, our study demonstrates that, on the one hand, hZBP1 acts as a highly potent cell death inducer, capable of causing significant tumor shrinkage in vivo, as demonstrated by our CDX model. This strong anti-tumor effect suggests that inducing hZBP1 expression is a promising new approach for treating solid cancers. On the other hand, we found that hZBP1’s capacity to trigger cell death and inflammatory responses is significantly enhanced compared to its mouse counterpart. These intrinsic species-specific differences in ZBP1 signaling underscore the challenges of translating findings from animal models to human therapies targeting this pathway.

## Methods

**Table:** Reagents and tools table

Reagent/resource	Reference or source	Identifier or catalog number
**Experimental models**		
C57BL/6N	Vital River (Beijing)	213
BALB/cNj-*Foxn1*^*nu*^/Gpt	GemPharmatech Co., Ltd.	Strain NO. D000521
A549	ATCC	CRM-CCL-185
HaCaT	ATCC	CRL-2404
HT-29	ATCC	HTB-38
HeLa	ATCC	CRM-CCL-2
HepG2	ATCC	HB-8065
HEK293T	ATCC	CRL-11268
Human primary lung fibroblasts	CTCC	CTCC-007-HUM
Immortalized MEFs	This paper	N/A
MC38	Procell	CL-0972
Murine primary lung fibroblasts	This paper	N/A
Bone marrow-derived macrophages	This paper	N/A
**Recombinant DNA**		
pCW-FLAG-EV	This paper	N/A
pCW-FLAG-hZBP1	This paper	N/A
pCW-FLAG-hZBP1 mutZα1	This paper	N/A
pCW-FLAG-hZBP1 mutZα2	This paper	N/A
pCW-FLAG-hZBP1 mutZα1-2	This paper	N/A
pCW-FLAG-mZBP1	This paper	N/A
pCW-FLAG-mZBP1 mutZα1-2	This paper	N/A
pCW-FLAG-ZBP1(h + m)	This paper	N/A
pCW-FLAG-ZBP1(m + h)	This paper	N/A
pCW-FLAG-hZBP1-SF	This paper	N/A
pCW-FLAG-hZBP1 mutR1	This paper	N/A
pCW-FLAG-hZBP1 mutR2	This paper	N/A
pCW-FLAG-hZBP1 mutR3	This paper	N/A
pCW-mZBP1 mutR1	This paper	N/A
pCW-mZBP1 mutR2	This paper	N/A
pCW-mZBP1 mutR3	This paper	N/A
pCW-FLAG-ZBP1(hZαR1 + mR23)	This paper	N/A
pCW-FLAG-ZBP1(mZαR1 + hR23)	This paper	N/A
pWPT-SV40 large T antigen	This paper	N/A
pcDNA3.1-HA-RIPK1	This paper	N/A
pMD2.G	addgene	https://www.addgene.org/12259/
psPAX2	addgene	https://www.addgene.org/12260/
lentiCRISPRv2-hygro	addgene	https://www.addgene.org/98291/
**Antibodies**		
Mouse Anti-ZBP1	AdipoGen	Cat#AG-20B-0010; RRID: AB_2490191
Rabbit anti-ZBP1	Cell Signaling Technology	Cat#60968S; RRID: AB_2799599
Rabbit anti-cleaved-caspase-8	Cell Signaling Technology	Cat#9496S; RRID: AB_561381
Rabbit anti-cleaved-caspase-3	Cell Signaling Technology	Cat#9661; RRID: AB_2341188
Rabbit anti-caspase-8	Cell Signaling Technology	Cat#4790; RRID: AB_10545768
Rabbit anti-caspase-3	Cell Signaling Technology	Cat#14220; RRID: AB_2798429
Rabbit anti-phospho-RIPK1 S166	Cell Signaling Technology	Cat#65746S; RRID: AB_2799693
Rabbit anti-RIPK1	Cell Signaling Technology	Cat#3493S; RRID: AB_2305314
Mouse anti-RIPK1	BD	Cat#610458; RRID: AB_397831
Rabbit anti-phospho-MLKL S358	Abcam	Cat#ab187091; RRID: AB_2619685
Rabbit anti-MLKL	Cell Signaling Technology	Cat#14993S; RRID: AB_2721822
Mouse anti-RIPK3	Santa Cruz	Cat#sc-374639; RRID: AB_10992232
Mouse anti-FADD	Santa Cruz	Cat#sc-271748; RRID: AB_10708849
Mouse anti-GAPDH	Proteintech	Cat#60004-1-Ig; RRID: AB_2107436
Rabbit anti-β-ACTIN	ABclonal	Cat#AC026; RRID: AB_2768234
Rabbit anti-GSDMD	HUABIO	Cat#ER1901-37; RRID: AB_3069329
Rabbit anti-HSP90	Proteintech	Cat#13171-1-AP; RRID: AB_2120924
Rabbit anti-ISG15	Cell Signaling Technology	Cat#2743S; RRID: AB_2126201
Rabbit anti-DYKDDDDK Tag (FLAG)	Cell Signaling Technology	Cat#14793; RRID: AB_2572291
Rabbit anti-HA Tag	Cell Signaling Technology	Cat#3724; RRID: AB_1549585
Rabbit anti-GFP Tag	Proteintech	Cat#50430-2-AP; RRID: AB_2572291
Mouse anti-ADAR1	Santa Cruz	Cat#sc-73408; RRID: AB_10992232
Mouse anti-dsRNA antibody(J2)	SCICONS	Cat#10010200; RRID: AB_2651015
Goat anti-mouse IgG (H + L) Cross-Adsorbed Secondary Antibody, Alexa Fluor™ 488	Thermo Fisher Scientific	Cat#A11001; RRID: AB_2534069
Goat anti-Rabbit IgG (H + L) Cross-Adsorbed Secondary Antibody, Alexa Fluor™ 594	Thermo Fisher Scientific	Cat#A11012; RRID: AB_2534079
**Oligonucleotides and other sequence-based reagents**		
sg*ZBP1*-1	Tsingke	AAGCCATCCAGATTGGACAC
sg*ZBP1*-2	Tsingke	GGACGATTTACCGCCCAGGT
sg*ZBP1*-3	Tsingke	TGGGACACAGCAATGAGATG
sg*RIPK1*-1	Tsingke	CCATGCGGCTGCCATAAAGA
sg*RIPK1*-2	Tsingke	GGCACCGCTAAGAAGAATGG
sg*RIPK1*-3	Tsingke	GGGAAGCGAATCCGGAAGCT
sg*RIPK1*-4	Tsingke	TGGAAAAGGCGTGATACACA
sg*RIPK3*	Tsingke	GTTTGTTAACGTAAACCGGA
sg*MLKL*	Tsingke	TTCCCTTAGCAGAATCCACG
sg*FADD*	Tsingke	CACGCTCTGTCAGGTTGCGG
si*ADAR1*-1 sense	SunYa	GCUGUUAGAAUAUGCCCAGUUTT
si*ADAR1*-1 antisense	SunYa	AACUGGGCAUAUUCUAACAGCTT
si*ADAR1*-2 sense	SunYa	GAGGAUGCAAAUCAAGAGAAATT
si*ADAR1*-2 antisense	SunYa	UUUCUCUUGAUUUGCAUCCUCTT
Forward primer for *ZBP1*	Tsingke	CTGGTGAAGGAATGCCAAGC
Reverse primer for *ZBP1*	Tsingke	TGTAGATGTCTTCCTCCCGTTG
Forward primer for *TNF*	Tsingke	AGCCCATGTTGTAGCAAACCC
Reverse primer for *TNF*	Tsingke	TCCCAAAGTAGACCTGCCCA
Forward primer for *NFKBIA*	Tsingke	TTTTGGTGTCCTTGGGTGCT
Reverse primer for *NFKBIA*	Tsingke	GTCATCATAGGGCAGCTCGT
Forward primer for *TNFAIP3*	Tsingke	GCCAAGAGAGATCACACCCC
Reverse primer for *TNFAIP3*	Tsingke	GTGCTCTCCAACACCTCTCC
Forward primer for *BIRC3*	Tsingke	TGGTGTTTTACTATCCAAAGAATGC
Reverse primer for *BIRC3*	Tsingke	TCAGCGGTAACAGCCCATTT
Forward primer for *ACTB*	Tsingke	CGCCGCCAGCTCACC
Reverse primer for *ACTB*	Tsingke	CACGATGGAGGGGAAGACG
Forward primer for *IFIT1*	Tsingke	ATTTACAGCAACCATGAGTACAAA
Reverse primer for *IFIT1*	Tsingke	TCCTCCACACTTCAGCAAGG
Forward primer for *ISG15*	Tsingke	TGGACAAATGCGACGAACCT
Reverse primer for *ISG15*	Tsingke	CCCTTGATCCTGCTCGGATG
Forward primer for *MX1*	Tsingke	GGCATAACCAGAGTGGCTGT
Reverse primer for *MX1*	Tsingke	TTCCTCCAGCAGATCCCTGA
Forward primer for *GAPDH*	Tsingke	GTCTCCTCTGACTTCAACAGCG
Reverse primer for *GAPDH*	Tsingke	ACCACCCTGTTGCTGTAGCCAA
Forward primer for *Zbp1*	Tsingke	GTCAGCACAATCCGGTCAAC
Reverse primer for *Zbp1*	Tsingke	GGGTAGAGGGTGAGAGGTCC
Forward primer for *Gapdh*	Tsingke	AGGTCGGTGTGAACGGATTT
Reverse primer for *Gapdh*	Tsingke	ACTGTGCCGTTGAATTTGCC
Forward primer for *Actb*	Tsingke	GGACTCCTATGTGGGTGACG
Reverse primer for *Actb*	Tsingke	ATGGCTACGTACATGGCTGG
Forward primer for *Ifit1*	Tsingke	GCAAGAGAGCAGAGAGTCAAGGC
Reverse primer for *Ifit1*	Tsingke	TGAAGCAGATTCTCCATGACCTG
Forward primer for *Isg15*	Tsingke	GAGCTAGAGCCTGCAGCAATG
Reverse primer for *Isg15*	Tsingke	TTCGTTCCTCACCAGGATGC
Forward primer for *Mx1*	Tsingke	TCAGGAGGTGGACCCTGAAG
Reverse primer for *Mx1*	Tsingke	ACCTGGTCCTGGCAGTAGAC
**Chemicals, enzymes and other reagents**		
Emricasan	Selleck	Cat#S7775
Q-VD-Oph	Selleck	Cat#S7311
Nec-1s	Selleck	Cat#S8641
GSK'872	Selleck	Cat#S8465
GSK'840	MCE	Cat#HY-104021
NSA	Selleck	Cat#S8251
Recombinant Human IFN-β	ABclonal	Cat#RP02774LQ
Recombinant Human IFN-γ	ABclonal	Cat#RP01038
Recombinant Mouse IFN-β	ABclonal	Cat#RP01076
Recombinant Mouse IFN-γ	ABclonal	Cat#RP01070
Pladienolide B	GLPBIO	Cat#GC18780
CBL0137	Med Chem Express	Cat#HY-18935A
Neutral Red	Macklin	Cat#553-24-2
HEPES	Sigma	Cat#7365-45-9
Doxycycline	Beyotime	Cat#ST039A
RNase A	Thermo Fisher Scientific	Cat#EN0531
ProLongTM Gold Antifade Mountant with DAPI	Thermo Fisher Scientific	Cat#P36931
Lipofectamine 2000 Reagent	Thermo Fisher Scientific	Cat#11668019
Anti-DYKDDDDK G1 Affinity Resin	GenScript	Cat#L00432
ChIP-grade Protein A/G magnetic beads	ABclonal	Cat#RM02915
PhosSTOP	Roche	4906837001
cOmplete™, Mini Protease Inhibitor Cocktail	Roche	4693124001
M-CSF	ABclonal	Cat#RP01216
**Software**		
Amersham ImageQuant 800	Cytiva	N/A
LSM880 confocal microscope	Zeiss	N/A
GraphPad Prism	Prism 10	N/A
Image J	N/A	N/A
SnapGene	N/A	N/A
ZEN	ZEN 2 (blue edition)	N/A
**Other**		
ECL SuperSignal West PicoPlus chemiluminescent substrate	Thermo Scientific	Cat#34578
Pierce™ BCA Protein Assay Kit	Thermo Fisher Scientific	Cat#23227

### Mice

Mice are all in the C57BL/6N background and were housed in the barrier facility at Zhejiang University under a 12 h–12 h light-dark cycle in ventilated cages with bedding at 22 °C and 30–60% humidity. Food (1010082, XIETONG SHENGWU) and water were provided according to standard conditions for mice. All mouse studies were conducted in accordance with protocol (ZJU20240766) approved by the Institutional Animal Care and Use Committee of Zhejiang University.

### Cell culture

Murine embryonic fibroblasts (MEFs) were isolated from pups at embryonic day 13.5 and immortalized via lentiviral transduction of the SV40 large T antigen. Murine primary lung fibroblasts were isolated from wild-type C57BL/6N mice of randomized sex and immortalized via lentiviral transduction of the SV40 large T antigen. HT-29 cells, A549 cells, HeLa cells, HaCaT cells, HepG2 cells, immortalized MEFs, MC38, BMDMs, and mouse primary lung fibroblasts were all maintained in DMEM (Gibco, C11995500CP) supplemented with 10% FBS (Yeasen, 40130ES76) and 1% penicillin/streptomycin (Yeasen, 60162ES76) in a 37 °C humidified incubator with 5% CO_2_. Human primary lung fibroblasts were purchased from CTCC and maintained in specialized culture medium (CTCC-HUM-0026-CM) supplemented with 10% FBS and 1% penicillin/streptomycin.

### Generation of bone marrow-derived macrophages

For isolation of primary BMDMs, tibias and femurs of healthy wild-type C57BL/6N mice of randomized sex were removed using sterile methods, and the bone marrow was washed with fresh media. Macrophage precursors were cultured and differentiated for 6 days in M-CSF (ABclonal, RP01216) at a final concentration of 20 ng/mL to macrophages and harvested on day 7.

### Inducible stable cell line generation

Plasmids used in this study were listed as follows: pCW-FLAG-EV, pCW-FLAG-hZBP1, pCW-FLAG-hZBP1 mutZα1, pCW-FLAG-hZBP1 mutZα2, pCW-FLAG-hZBP1 mutZα1-2, pCW-FLAG-mZBP1, pCW-FLAG-mZBP1 mutZα1-2, pCW-FLAG-ZBP1(h + m), pCW-FLAG-ZBP1(m + h), pCW-FLAG-hZBP1-SF, pCW-FLAG-hZBP1 mutR1, pCW-FLAG-hZBP1 mutR2, pCW-FLAG-hZBP1 mutR3, pCW-mZBP1 mutR1, pCW-mZBP1 mutR2, pCW-mZBP1 mutR3, pCW-FLAG-ZBP1(hZαR1 + mR23), and pCW-FLAG-ZBP1(mZαR1 + hR23).

To generate all sorts of inducible overexpressing stable cells, lentiviral infection was used. Briefly, FLAG-tagged wild-type ZBP1 and ZBP1 mutants were subcloned into lentiviral pCW plasmid. HEK293T cells were co-transfected with pCW lentiviral vector and packaging plasmids psPAX2 (Addgene, https://www.addgene.org/12260/) and pMD2.G (Addgene, https://www.addgene.org/12259/) at a 4:3:1 ratio. Lentivirus-containing medium was collected 48 h post transfection, filtered through a 0.45-μm filter, and used to infect target cells in the presence of 10 μg/mL polybrene (Yeasen, 40804ES76) for 48 h. Infected cells were selected using 4 μg/mL puromycin (Sangon #A610593) for another 2 days.

### Cell viability assays

Cells were seeded in 96-well plates (1 × 10^4^/well for HT-29, 3 × 10^3^/well for immortalized MEFs, and 5 × 10^3^/well for A549, HaCaT, HeLa, and HepG2). After 2 days, cells were pretreated with indicated amounts of emricasan (Selleck, S7775), Nec-1s (Selleck, S8641), GSK’840 (MCE, HY-104021), GSK’872 (Selleck, S8465), or necrosulfonamide (Selleck, S8251) 40 min before indicated amounts of doxycycline (Beyotime, ST039A) were added. Cell viability was measured by neutral red (Macklin, 553-24-2) uptake as described.

### CRISPR-Cas9-mediated KO cell generation

ZBP1 KO, RIPK1 KO, RIPK3 KO, MLKL KO, and FADD KO in HT-29 and HeLa cells were generated by CRISPR-Cas9 technology. Independent sgRNAs were designed by the Zhang laboratory CRISPR Design Tool. Annealed oligonucleotides were ligated into the lentiCRISPRv2-hygro plasmid (Addgene plasmid #98291). Lentiviruses were packaged in HEK293T as described above, followed by hygromycin (1 mg/mL) selection for 4 days. The efficiency of KO was examined by Western blotting. For ZBP1 KO, cells were re-plated in 96-well plates for colony formation. To verify ZBP1 KO by DNA sequencing, fragments containing predicted Cas9 cleavage sites were PCR amplified and cloned into the pMD18-T vector using TA cloning. Six clones for each PCR were sequenced.

### Cell transfection

For HeLa cell transfection, cells were seeded on 12-well plates one day prior to transfection to allow cell attachment. The following day, cells were transfected with FLAG-tagged hZBP1 and mZBP1 using PEI at a 1:3 ratio according to the manufacturer’s protocol. The medium was replaced with Dox (1 μg/mL) addition 6–8 h post transfection. HeLa cells were harvested 12 h after Dox treatment and processed for Western blotting analysis.

### Western blotting

Western blotting was performed using standard protocol. Briefly, cells were lysed by 2% SDS, followed by denaturation in Laemmli buffer. Lysates were separated by SDS-PAGE, then transferred to PVDF membranes (Millipore, IPVH00010) and analyzed by immunoblotting with desired primary antibodies. Secondary horseradish peroxidase (HRP)-coupled antibodies were used to detect proteins using chemiluminescence with ECL SuperSignal West PicoPlus chemiluminescent substrate (Thermo Scientific, 34578), and the signal was measured with Amersham ImageQuant 800 (Cytiva).

### Immunoprecipitation

For FLAG-immunoprecipitation, cells were rinsed once with ice-cold PBS and lysed in ice-cold NP40 lysis buffer (50 mM Tris pH 7.8, 150 mM NaCl, and 1% NP40) supplemented with cOmplete™ Mini (Roche, 4693124001) and PhosSTOP (Roche, 4906837001). The soluble fractions of cell lysates were isolated by centrifugation at 14,000 × *g* for 15 min at 4 °C and quantified by the BCA kit. Equivalent amounts of protein were incubated with 20 μL FLAG resin (GenScript, #L00432S) with rotation at 4 °C overnight. Resins were collected with centrifugation and washed with NP40 lysis buffer five times, and then interacting proteins were boiled at 95 °C for 10 min with 1 × protein loading buffer before loading and subjected to electrophoresis.

For dsRNA immunoprecipitation, equivalent amounts of supernatant proteins were incubated with ChIP-grade Protein A/G magnetic beads (ABclonal, RM02915) and J2 antibody with rotation at 4 °C overnight. Beads were collected with a magnetic separation rack and washed with NP40 lysis buffer five times. Beads were boiled at 95 °C for 10 min before loading and subjected to electrophoresis.

### Oligomer analysis

For oligomer analysis, cells were lysed in ice-cold NP40 lysis buffer (50 mM Tris pH 7.8, 150 mM NaCl, 1% NP40) supplemented with cOmplete™ Mini (Roche, 4693124001) and PhosSTOP (Roche, 4906837001). Lysates were centrifuged at 12000 × *g* for 15 min at 4 °C, and the supernatant was removed for analysis as NP40-soluble fractions. The pellet was washed once with NP40 wash buffer and resuspended in 6 M urea solution at 4 °C overnight. The next day, protein samples were centrifuged at 12000 × *g* for 10 min at 4 °C. The supernatant was divided equally into two parts and prepared with 4× protein loading buffer with or without β-mercaptoethanol, respectively. All samples were boiled at 95 °C for 10 min before loading and subjected to electrophoresis.

### Immunofluorescence

Cells were seeded on coverslips in 24-well plates and allowed to adhere for 24–36 h before experiments. Following treatment, cells were washed once with 1X PBS and fixed with 4% paraformaldehyde (PFA) for 15 min at room temperature. After three washes with PBS, cells were permeabilized with 0.25% Triton X-100 in PBS for 10 min at room temperature. After another three washes with PBS, cells were blocked with 3% BSA in PBST for 30 min. The cells were then incubated overnight with the following primary antibodies at 4 °C: FLAG (1:100), RIPK1 (1:50), and ZBP1 (1:200). After three washes in PBST for 15 min, cells were incubated for 1.5 h at room temperature with a 1:500 dilution of fluorophore-conjugated secondary antibodies and then mounted in ProLong™ Gold Antifade Mountant with DAPI (Invitrogen, P36931) following three additional washes in PBST for 15 min. Images were taken by the LSM880 confocal microscope (Zeiss).

### Differential interference contrast (DIC) microscopy

HT-29 overexpressing FLAG-tagged hZBP1 or FLAG-tagged mZBP1 and immortalized MEFs overexpressing FLAG-tagged hZBP1 or FLAG-tagged mZBP1 were seeded into 35 mm glass-bottom cell culture dishes and stimulated with or without Dox (1 μg/mL) or combinations of Dox and emricasan (5 μM) for 9 h before DIC microscopy imaging. High-resolution images (2048 × 2048 pixels) were saved for subsequent morphological analysis.

### Fluorescence recovery after photobleaching (FRAP)

FRAP experiments were performed on a Zeiss LSM880 microscope with a 63 × oil immersion objective. For FRAP in living cells, cells were seeded into 35-mm glass-bottom cell culture dishes. HT-29 cells expressing GFP-tagged hZBP1 were treated with 1 μg/mL Dox for 10 h, and GFP-positive assemblies were photobleached using a laser intensity of 50% at 488 nm (for GFP), and recovery was recorded for the indicated time. The raw data were exported, the pre-bleached fluorescence intensity was normalized to 1, and the signal after bleaching was normalized to the pre-bleached level.

### SiRNA-mediated knockdown

For siRNA knockdown in HeLa, cells were plated in six-well plates or 96-well plates. After 100 ng/mL IFNβ priming for 24 h, cells at ~50% confluency were transfected with 20 nM siRNAs using Lipofectamine RNAiMAX (Thermo Fisher) following the manufacturer’s protocol. siRNAs against human *ADAR1* were synthesized by SunYa.

### RNA isolation and real-time PCR

To determine mRNA expression levels, total RNA was isolated from cultured cells using TRIzol^TM^ (Thermo, 15596018). cDNA was synthesized by reverse transcription using 4 × Super Mix (Yeasen, 11141ES60) and subjected to real-time PCR with gene-specific primers in the presence of SYBR Green (Yeasen, 11201ES08). Relative abundance of mRNA was calculated by normalization to *GAPDH* or *ACTB* mRNA.

### RNA-seq data analysis

RNA was extracted using standard procedures, and the resulting samples were sent to Shanghai Majorbio Bio-pharm Biotechnology for RNA purification, reverse transcription, library construction, and sequencing. The libraries were sequenced with the NovaSeq X Plus sequencer (2 × 150 bp read length). The raw paired-end reads were trimmed and quality controlled by fastp with default parameters. Then, clean reads were separately aligned to the reference genome with orientation mode using HISAT2 software. The mapped reads of each sample were assembled by StringTie in a reference-based approach. DESeq2 (http://bioconductor.org/packages/stats/bioc/DESeq2/) was utilized to identify differentially expressed genes (DEGs). GO functional enrichment and KEGG pathway analysis were carried out by Goatools and KOBAS, respectively.

### Xenograft mouse models

Female BALB/c nude mice at 6 weeks of age were purchased from GemPharmatech Co., Ltd. (Jiangsu, China) and were distributed in four groups of six mice each. The animals were xenografted with 2 × 10^6^ HT-29 cells (overexpressed with either FLAG-EV or FLAG-hZBP1 WT) resuspended in 50 μL PBS with 50 μL Cultrex (RD, 3433-005-01) and subcutaneously inoculated in both flanks of the animals. When the tumor volume grew to 50–100 mm^3^ at day 11 post inoculation, mice were administered with or without doxycycline (2 mg/mL) dissolved in drinking water. The sizes of xenografts were measured with a vernier caliper every 3 days. Tumor volumes were calculated with the formula: (length × width^2^)/2. All mice were sacrificed to obtain the tumors at day 18 post inoculation.

### Statistical analysis

All experiments were repeated at least three times. Data shown in graphs are the mean or mean ± SEM. Statistical significance was determined using an unpaired parametric *t*-test with Welch’s correction (for two-group comparisons) or one-way ANOVA followed by Tukey’s post hoc test (for multiple comparisons). All statistical tests listed in the figure legends were two-sided and were performed using GraphPad Prism.

## Supplementary information

Peer Review File

Source data Fig. 1

Source data Fig. 2

Source data Fig. 3

Source data Fig. 4

Source data Fig. 5

Source data Fig. 6

Figure EV1 Source Data

Figure EV2 Source Data

Figure EV3 Source Data

Figure EV4 Source Data

Figure EV5 Source Data

Expanded View Figures

## Data Availability

The datasets generated during and/or analyzed during the current study are available from the corresponding author on reasonable request. The original RNA-sequencing data are uploaded at the Gene Expression Omnibus (GEO) under accession GSE306290: https://www.ncbi.nlm.nih.gov/geo/query/acc.cgi?acc=GSE306290; GSE306289: https://www.ncbi.nlm.nih.gov/geo/query/acc.cgi?acc=GSE306289. The source data of this paper are collected in the following database record: biostudies:S-SCDT-10_1038-S44319-026-00866-6.
