# Supplementary material for: Human ZBP1 is a potent inducer of cell death through mechanisms divergent from mouse ZBP1
Source: EMBO Rep. 2026 Jul 11;27(17):5062–91. doi: 10.1038/s44319-026-00866-6 (PMC13554060; doi:10.1038/s44319-026-00866-6)
Supplement: Supplementary file 1 [file 44319_2026_866_MOESM13_ESM.pdf]

# Expanded View Figures

**Figure EV1. Human ZBP1 triggers spontaneous cell death via RIPK1.**

(**A**) Cell viability measured by neutral red uptake in HT-29 expressing Dox-inducible FLAG-EV, FLAG-hZBP1 stimulated with or without Dox (1 μg/mL) or combinations of Dox and emricasan (5 μM), Q-VD-OPh (10 μM), Nec-1s (10 μM), GSK'840 (3 μM), and NSA (5 μM) for 24 h. (**B**) Immunoblot analysis of total lysates in HT-29 expressing Dox-inducible FLAG-hZBP1 stimulated with or without Dox (1 μg/mL) or combinations of Dox (1 μg/mL) and emricasan (5 μM) for 10 h. * nonspecific band. (**C**) Immunoblot analysis of total lysates in HepG2 expressing Dox-inducible FLAG-EV, hZBP1-FL, and hZBP1-SF stimulated with or without Dox (1 μg/mL) for 10 h. (**D**) Cell viability measured by neutral red uptake in HepG2 expressing Dox-inducible FLAG-EV, hZBP1-FL, and hZBP1-SF stimulated with or without Dox (1 μg/mL) for 24 h. (**E**) Immunoblot analysis of total lysates in HeLa expressing Dox-inducible FLAG-EV and hZBP1 stimulated with or without Dox (1 μg/mL) for 10 h. (**F**) Cell viability measured by neutral red uptake in HeLa expressing Dox-inducible FLAG-EV and FLAG-hZBP1 stimulated with or without Dox (1 μg/mL) or combinations of Dox and emricasan (5 μM), Nec-1s (10 μM), and NSA (2 μM) for 48 h. (**G**) Cell viability measured by neutral red uptake in HaCaT expressing Dox-inducible FLAG-hZBP1 stimulated with or without Dox (1 μg/mL) or combinations of Dox and emricasan (5 μM), Nec-1s (10 μM), GSK'840 (3 μM), and NSA (5 μM) for 24 h. (**H**) Immunoblot analysis of total lysates in HaCaT expressing Dox-inducible FLAG-EV and hZBP1 stimulated with Dox (1 μg/mL) or a combination of Dox (1 μg/mL) and emricasan (5 μM) for 10 h. (**I**) Immunoblot analysis of total lysates in A549 expressing Dox-inducible FLAG-hZBP1 stimulated with or without Dox (1 μg/mL) for 10 h. (**J**) Cell viability measured by neutral red uptake in A549 expressing Dox-inducible FLAG-hZBP1 stimulated with or without Dox (1 μg/mL) for 24 h. (**K**) Immunoblot analysis of total lysates in WT, RIPK1 KO, RIPK3 KO, MLKL KO, and FADD KO HT-29 expressing Dox-inducible FLAG-hZBP1 stimulated with or without Dox (1 μg/mL) for 10 h. (**L**) Cell viability measured by neutral red uptake in FADD KO, RIPK3 KO, and MLKL KO HT-29 expressing Dox-inducible FLAG-hZBP1 stimulated with or without Dox (1 μg/mL), or combinations of Dox and emricasan (5 μM), Nec-1s (10 μM), GSK'840 (3 μM), and NSA (5 μM) for 24 h. (**M**) Immunoblot analysis of FLAG immunoprecipitates and total lysates (Input) from A549 and HaCaT expressing Dox-inducible FLAG-EV and FLAG-hZBP1 stimulated with Dox (1 μg/mL) for 12 h. (**N**, **O**) FRAP analysis of GFP-hZBP1 assemblies formed in the nucleus (**N**) and cytosol (**O**) in HT-29 expressing Dox-inducible GFP-hZBP1 stimulated with Dox (1 μg/mL) for 10 h. Left, representative confocal images of assemblies before and after bleaching. Right, quantification of the GFP fluorescence recovery curves. Scale bars, 2 μm in (**N**) and 1 μm in (**O**). All experiments were replicated at least three times. Data with error bars are mean ± SEM. Data in panels (**A**, **D**, **F**, **G**, **J**, **L**) are pooled and analyzed from at least three independent biological experiments. *P* values were calculated by unpaired parametric *t*-test with Welch's correction (**D**, **F**, **G**, **J**) or one-way ANOVA with Tukey's post hoc test for multiple comparisons (**A**, **L**). Source data are available online for this figure.


© The Author(s)

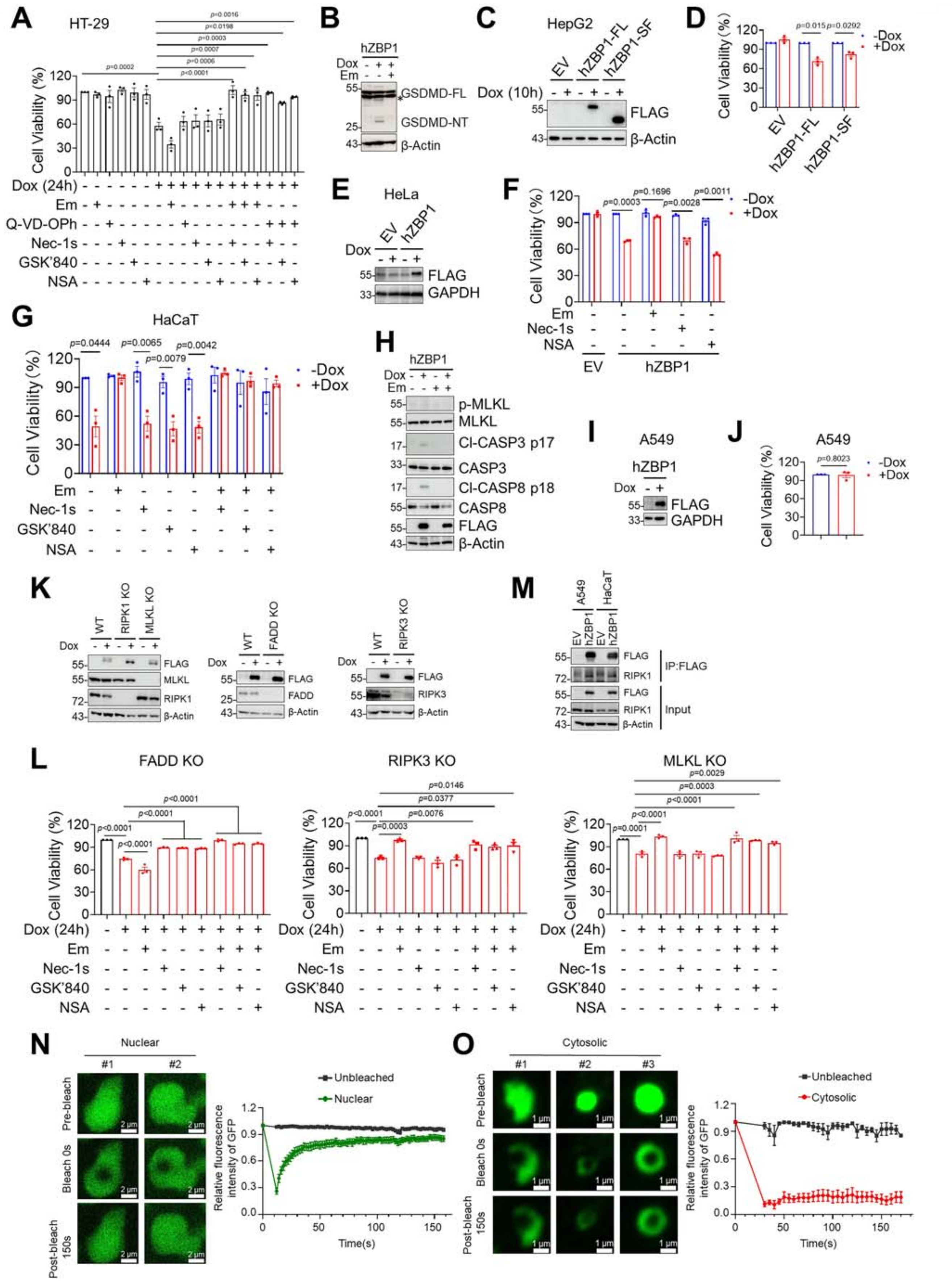
A
HT-29
Cell Viability (%)
Dox (24h)
Em
Q-VD-OPh
Nec-1s
GSK'840
NSA
B
hZBP1
GSDMD-FL
GSDMD-NT
β-Actin
C
HepG2
EV
hZBP1-FL
hZBP1-SF
Dox (10h)
FLAG
β-Actin
D
-Dox
+Dox
E
HeLa
FLAG
GAPDH
F
G
HaCaT
H
p-MLKL
MLKL
Cl-CASP3 p17
CASP3
Cl-CASP8 p18
CASP8
I
A549
J
K
WT
RIPK1 KO
MLKL KO
FADD KO
RIPK3 KO
RIPK1
FADD
RIPK3
M
IP:FLAG
Input
L
N
Nuclear
Pre-bleach
Bleach 0s
Post-bleach 150s
Unbleached
Relative fluorescence intensity of GFP
Time(s)
O
Cytosolic

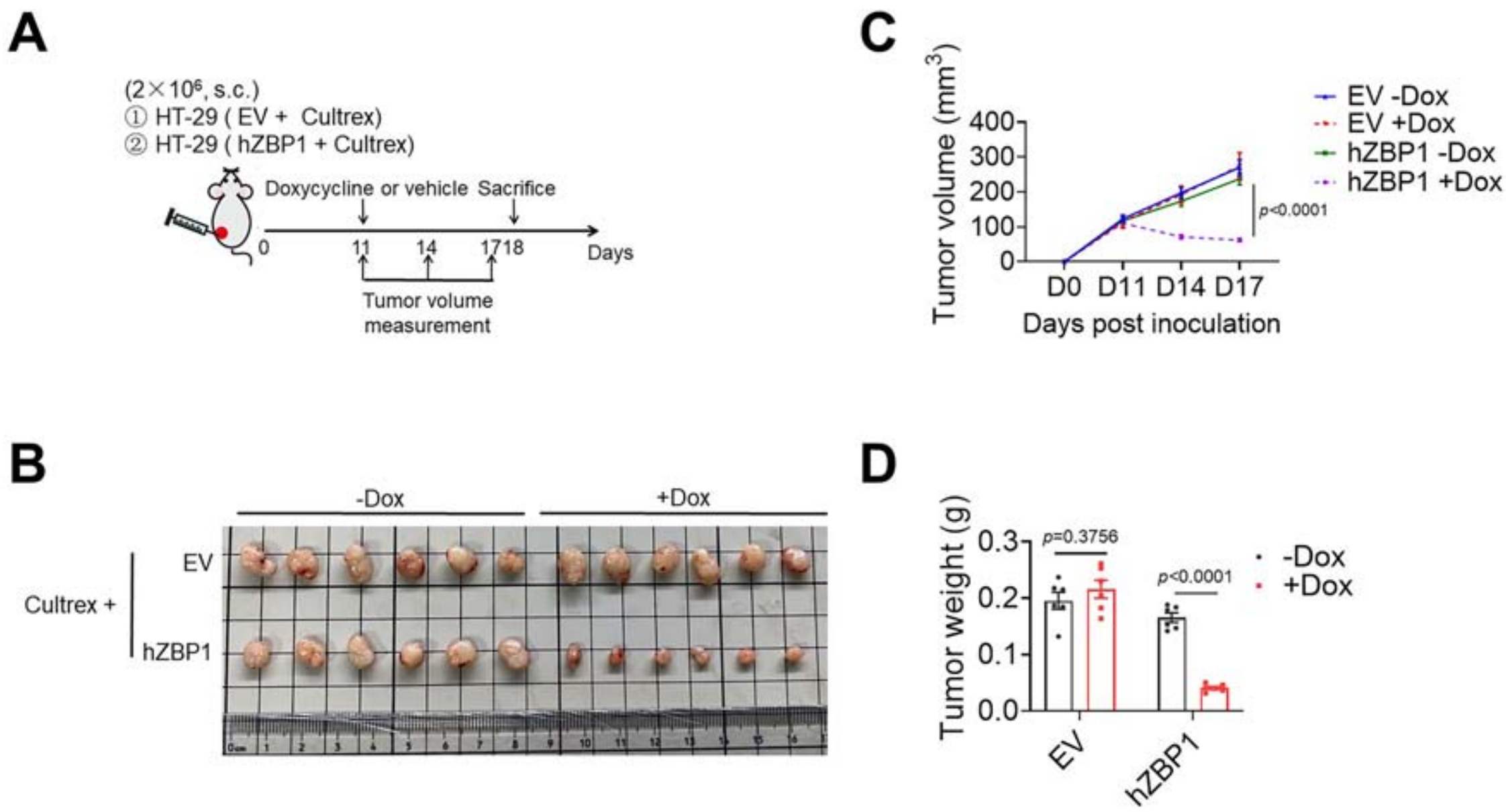


**Figure EV2. Human ZBP1 exerts potent anti-tumor effects in vivo.**

(**A**) Treatment schedule of nude mice bearing subcutaneous (s.c.) HT-29. Treatments were initiated at day 11 post inoculation when tumors were palpable (~100 $mm^3$). (**B**) Picture representation of tumors at day 18 post inoculation. (**C**) Individual tumor growth curves of Dox-treated and untreated HT-29 tumors at the indicated time points. (**D**) The tumor weight of nude mice bearing HT-29 treated with or without Dox. $n = 6$ mice for each group. Data with error bars are mean ± SEM. *P* values were calculated by unpaired parametric *t*-test with Welch's correction (**C**, **D**). Source data are available online for this figure.


© The Author(s)

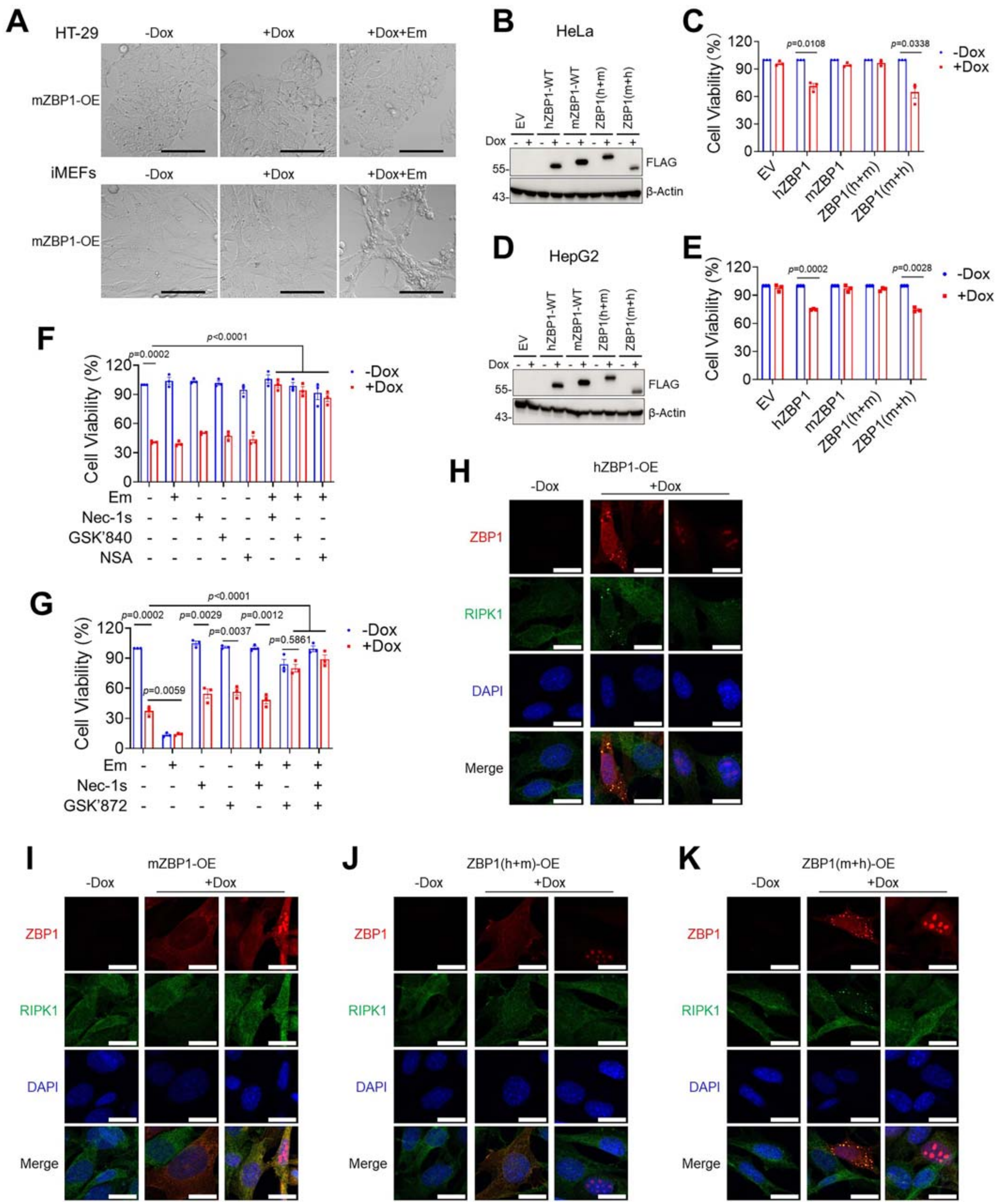
A
HT-29
-Dox
+Dox
+Dox+Em
mZBP1-OE
iMEFs
B
HeLa
EV
hZBP1-WT
mZBP1-WT
ZBP1(h+m)
ZBP1(m+h)
Dox
FLAG
β-Actin
C
Cell Viability (%)
p=0.0108
p=0.0338
D
HepG2
E
p=0.0002
p=0.0028
F
p<0.0001
Em
Nec-1s
GSK'840
NSA
G
p=0.0059
p=0.0029
p=0.0037
p=0.0012
p=0.5861
GSK'872
H
hZBP1-OE
ZBP1
RIPK1
DAPI
Merge
I
mZBP1-OE
J
ZBP1(h+m)-OE
K
ZBP1(m+h)-OE

**Figure EV3. C-terminal RHIM domains enable hZBP1 to induce spontaneous cell death.**

(**A**) Representative cell images of HT-29 cells expressing Dox-inducible FLAG-mZBP1 (top) and immortalized MEFs expressing Dox-inducible FLAG-mZBP1 (bottom) stimulated with or without Dox (1 µg/mL) or combinations of Dox (1 µg/mL) and emricasan (5 µM) for 10 h. Scale bars, 100 µm. (**B**, **D**) Immunoblot analysis of total lysates in HeLa (**B**) and HepG2 (**D**) expressing Dox-inducible FLAG-EV, FLAG-hZBP1, FLAG-mZBP1, FLAG-ZBP1(h + m), and FLAG-ZBP1(m + h) stimulated with or without Dox (1 µg/mL) for 10 h. (**C**, **E**) Cell viability measured by neutral red uptake in HeLa (**C**) and HepG2 (**E**) expressing Dox-inducible FLAG-EV, FLAG-hZBP1, FLAG-mZBP1, FLAG-ZBP1(h + m), and FLAG-ZBP1(m + h) stimulated with or without Dox (1 µg/mL) for 24 h. (**F**) Cell viability measured by neutral red uptake in HT-29 expressing Dox-inducible FLAG-ZBP1(m + h) stimulated with or without Dox (1 µg/mL) or combinations of Dox and emricasan (5 µM), Nec-1s (10 µM), GSK'840 (3 µM), and NSA (5 µM) for 24 h. (**G**) Cell viability measured by neutral red uptake in immortalized MEFs expressing Dox-inducible FLAG-ZBP1(m + h) stimulated with or without Dox (1 µg/mL) or combinations of Dox and emricasan (5 µM), Nec-1s (10 µM), and GSK'872 (3 µM) for 24 h. (**H–K**) Representative confocal images of immortalized MEFs expressing Dox-inducible FLAG-hZBP1 (**H**), FLAG-mZBP1 (**I**), FLAG-ZBP1(h + m) (**J**), and FLAG-ZBP1(m + h) (**K**) stimulated with or without Dox (1 µg/mL) for 10 h. Scale bars, 20 µm. All experiments were replicated at least three times. Data with error bars are mean ± SEM. Data in panels (**C**, **E**, **F**, **G**) are pooled and analyzed from at least three independent biological experiments. *P* values were calculated by unpaired parametric *t*-test with Welch's correction (**C**, **E**) or one-way ANOVA with Tukey's post hoc test for multiple comparisons (**F**, **G**). Source data are available online for this figure.

 © The Author(s)

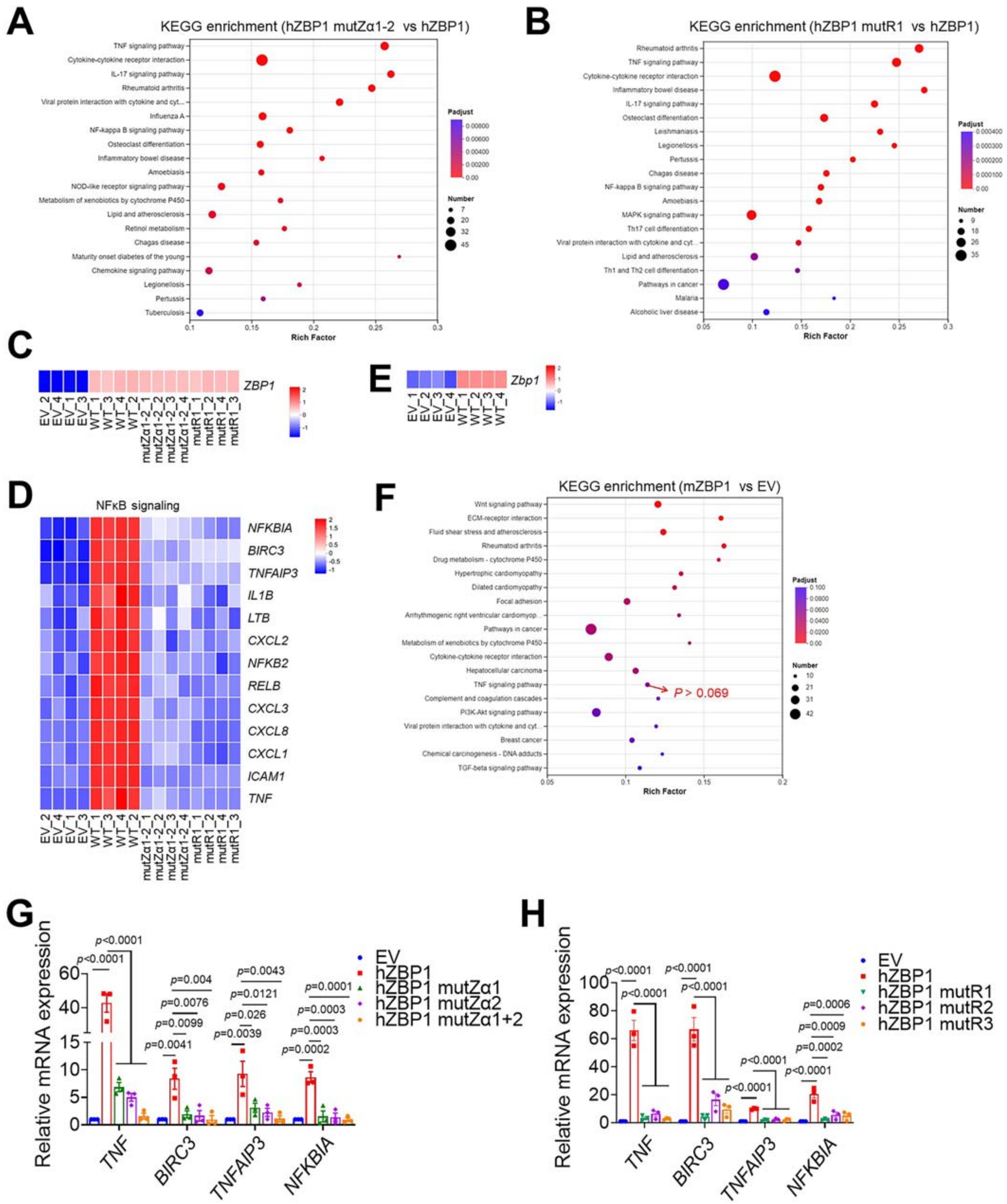
A
KEGG enrichment (hZBP1 mutZα1-2 vs hZBP1)
Rich Factor
B
KEGG enrichment (hZBP1 mutR1 vs hZBP1)
Rich Factor
C
ZBP1
D
NFκB signaling
NFKBIA
BIRC3
TNFAIP3
IL1B
LTB
CXCL2
NFKB2
RELB
CXCL3
CXCL8
CXCL1
ICAM1
TNF
E
Zbp1
F
KEGG enrichment (mZBP1 vs EV)
P > 0.069
Rich Factor
G
Relative mRNA expression
EV
hZBP1
hZBP1 mutZα1
hZBP1 mutZα2
hZBP1 mutZα1+2
TNF
BIRC3
TNFAIP3
NFKBIA
H
Relative mRNA expression
EV
hZBP1
hZBP1 mutR1
hZBP1 mutR2
hZBP1 mutR3
TNF
BIRC3
TNFAIP3
NFKBIA

**Figure EV4. Zα and RHIM domains contribute to hZBP1-mediated inflammation.**

(**A**, **B**) Dot plot of the KEGG enrichment analysis for DEGs compared between HT-29 expressing Dox-inducible FLAG-hZBP1 mutZα1-2 (**A**) and FLAG-hZBP1 mutR1 (**B**) with FLAG-hZBP1 WT. (**C**) Heatmap analysis of ZBP1 expression compared between HT-29 expressing Dox-inducible FLAG-EV, FLAG-hZBP1, FLAG-hZBP1 mutZα1-2, and FLAG-hZBP1 mutR1. (**D**) Heatmap analysis of DEGs enriched in NFκB signaling compared between HT-29 expressing Dox-inducible FLAG-EV, FLAG-hZBP1 WT, FLAG-hZBP1 mutZα1-2, and FLAG-hZBP1 mutR1. (**E**) Heatmap analysis of *Zbp1* expression compared between immortalized MEFs expressing Dox-inducible FLAG-EV and FLAG-mZBP1. (**F**) Dot plot of the KEGG enrichment analysis for DEGs compared between immortalized MEFs expressing Dox-inducible FLAG-EV and FLAG-mZBP1. (**G**, **H**) qRT-PCR analysis of mRNA expression of the indicated genes in HT-29 expressing Dox-inducible FLAG-EV, FLAG-hZBP1 WT, FLAG-hZBP1 Zα mutants (**G**), and FLAG-hZBP1 RHIM mutants (**H**) stimulated with or without Dox (1 μg/mL) for 10 h. All experiments were replicated at least three times. Data with error bars are mean ± SEM. Data in panels (**G**, **H**) are pooled and analyzed from at least three independent biological experiments. *P* values were calculated by one-way ANOVA with Tukey's post hoc test for multiple comparisons (**G**, **H**). Source data are available online for this figure.

 © The Author(s)

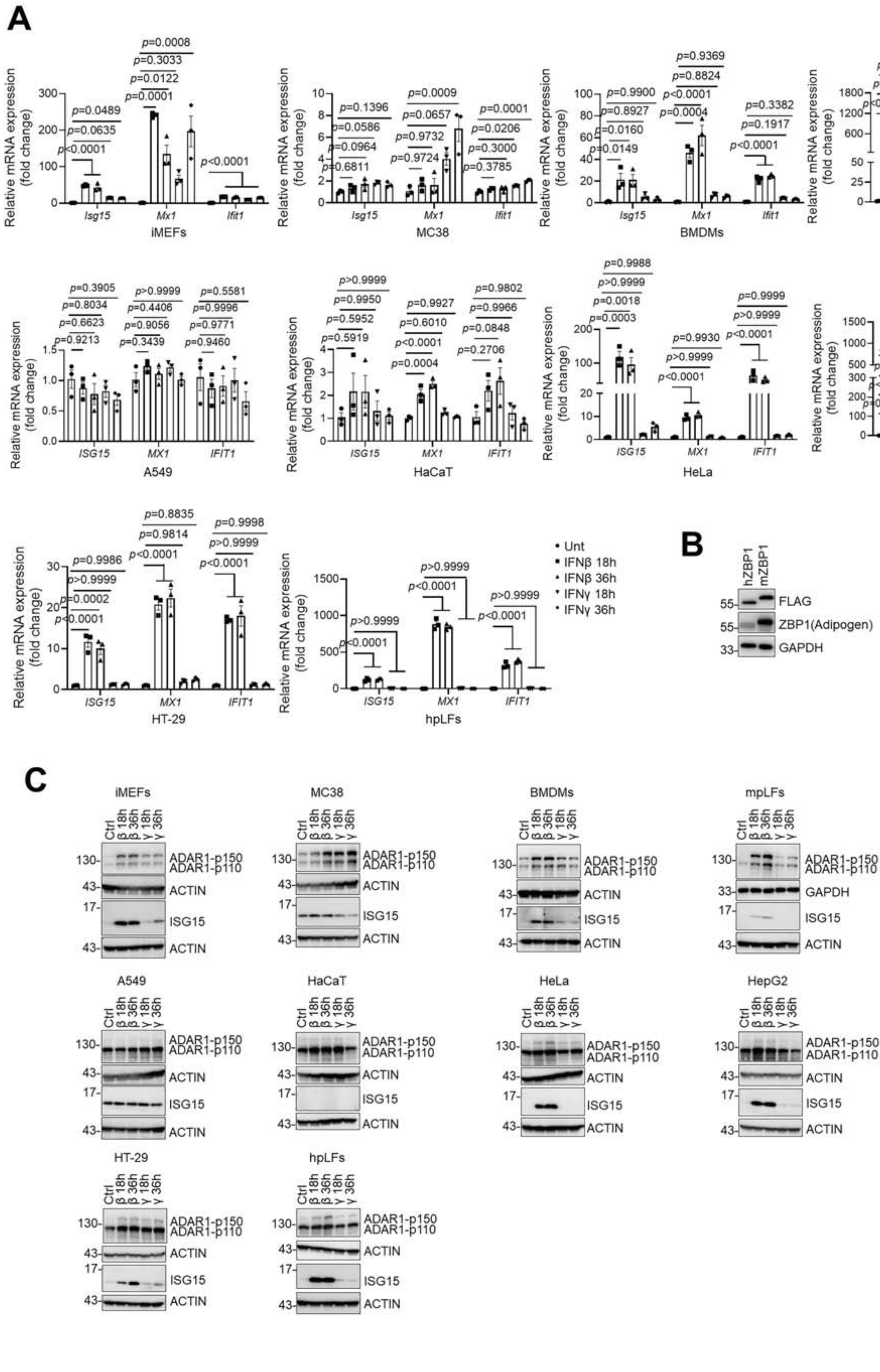

A
Relative mRNA expression (fold change)
Isg15
Mx1
Ifit1
iMEFs
MC38
BMDMs
mpLFs
ISG15
MX1
IFIT1
A549
HaCaT
HeLa
HepG2
HT-29
hpLFs
Unt
IFNβ 18h
IFNβ 36h
IFNγ 18h
IFNγ 36h
B
hZBP1
mZBP1
FLAG
ZBP1(Adipogen)
GAPDH
C
Ctrl
β 18h
β 36h
γ 18h
γ 36h
ADAR1-p150
ADAR1-p110
ACTIN
ISG15
GAPDH

**Figure EV5. ISG expression in human and mouse cells upon IFN treatment.**

(**A**) qRT-PCR analysis of mRNA expression of the indicated ISGs in different human and murine cells stimulated with or without IFNβ (20 ng/mL) or IFNγ (20 ng/mL) for 18 or 36 h. (**B**) Immunoblot analysis of total lysates in ZBP1 KO HeLa cells transfected with FLAG-hZBP1 and FLAG-mZBP1, and then blotted for FLAG and ZBP1 (Adipogen). (**C**) Immunoblot analysis of total lysates in different human and murine cells stimulated with or without IFNβ (20 ng/mL) or IFNγ (20 ng/mL) for 18 or 36 h, and then blotted for ADAR1-p150 and ISG15. All experiments were replicated at least three times. Data with error bars are mean ± SEM. Data from panel (**A**) are pooled and analyzed from at least three independent biological experiments. *P* values were calculated by one-way ANOVA with Tukey's post hoc test for multiple comparisons (**A**). Source data are available online for this figure.

 © The Author(s)
