# Supplementary material for: Human ZBP1 is a potent inducer of cell death through mechanisms divergent from mouse ZBP1
Source: EMBO Rep. 2026 Jul 11;27(17):5062–91. doi: 10.1038/s44319-026-00866-6 (PMC13554060; doi:10.1038/s44319-026-00866-6)
Supplement: Supplementary file 2 [file 44319_2026_866_MOESM1_ESM.pdf]

EMBO reports

Human ZBP1 Is a Potent Inducer of Cell Death Through Mechanisms Divergent from Mouse ZBP1

Huipeng Jiao, Fei Lu, Linghuan Tang, Zichao Tian, Jialiang Tian, Yinghao Fu, Anmin Gao, Heng Dong, Jianxiang Chen, Huasong Lu, chun Kim, Dengming Lai, Jinfa Tou, Chun Zhou, Juan Lin, and Jianfeng He

Corresponding author(s): Huipeng Jiao (hjiao@zju.edu.cn) , Juan Lin (linjuan2020@xmu.edu.cn), Jinfa Tou (toujinfa@zju.edu.cn)

Review Timeline:

Submission Date:	16th Nov 25
Editorial Decision:	21st Jan 26
Revision Received:	24th Apr 26
Editorial Decision:	22nd May 26
Revision Received:	6th Jun 26
Accepted:	12th Jun 26

Editor: Martina Rembold

Transaction Report:

(Note: With the exception of the correction of typographical or spelling errors that could be a source of ambiguity, letters and reports are not edited. Depending on transfer agreements, referee reports obtained elsewhere may or may not be included in this compilation. Referee reports are anonymous unless the Referee chooses to sign their reports.)

Dear Dr. Jiao

Thank you for the submission of your manuscript to EMBO reports. I sincerely apologize for the delay in handling it, but we have now received the full set of referee reports that are all pasted below.

As you will see, the referees acknowledge that the findings are potentially interesting and consider the data overall solid but they raise also a number of concerns and have suggestions how to strengthen the manuscript that all seem pertinent and should be addressed.

I would thus like to invite you to revise your manuscript with the understanding that the referee concerns must be fully addressed and their suggestions taken on board. Please address all referee concerns in a complete point-by-point response. Acceptance of the manuscript will depend on a positive outcome of a second round of review. It is EMBO reports policy to allow a single round of major revision only and acceptance or rejection of the manuscript will therefore depend on the completeness of your responses included in the next, final version of the manuscript.

We realize that it is difficult to revise to a specific deadline. In the interest of protecting the conceptual advance provided by the work, we recommend a revision within 3 months (23rd Apr 2026). Please discuss the revision progress ahead of this time with the editor if you require more time to complete the revisions.

You can either publish the study as a short report or as a full article. For short reports, the revised manuscript should not exceed 27,000 characters (including spaces but excluding materials & methods and references) and 5 main plus 5 expanded view figures. The results and discussion sections must further be combined, which will help to shorten the manuscript text by eliminating some redundancy that is inevitable when discussing the same experiments twice. For a normal article there are no length limitations, but it should have more than 5 main figures and the results and discussion sections must be separate. In both cases, the entire materials and methods must be included in the main manuscript file.

IMPORTANT NOTE: we perform an initial quality control of all revised manuscripts before re-review. Your manuscript will FAIL this control and the handling will be DELAYED if the following APPLIES:
1) A data availability section providing access to data deposited in public databases is missing. If you have not deposited any data, please add a sentence to the data availability section that explains that.
2) Your manuscript contains statistics and error bars based on n=2. Please use scatter blots in these cases. No statistics should be calculated if n=2.

When submitting your revised manuscript, please review the submission guidelines (https://link.springer.com/journal/44319/submission-guidelines#cms-Revised-submissions) and carefully review the instructions that follow below. Failure to include requested items will delay the evaluation of your revision.

1) a .docx formatted version of the manuscript text (including legends for main figures, EV figures and tables). Please make sure that the changes are highlighted to be clearly visible.

2) individual production quality figure files as .eps, .tif, .jpg (one file per figure). See hhttps://media.springernature.com/original/springer-cms/rest/v1/content/27825798/data/v1 for more info on how to prepare your figures.

3) We replaced Supplementary Information with Expanded View (EV) Figures and Tables that are collapsible/expandable online. A maximum of 5 EV Figures can be typeset. EV Figures should be cited as 'Figure EV1, Figure EV2" etc... in the text and their respective legends should be included in the main text after the legends of regular figures.

- For the figures that you do NOT wish to display as Expanded View figures, they should be bundled together with their legends in a single PDF file called *Appendix*, which should start with a short Table of Content. Appendix figures should be referred to in the main text as: "Appendix Figure S1, Appendix Figure S2" etc. See 'Expanded View' section of the submission guidelines.

- Additional Tables/Datasets should be labeled and referred to as Table EV1, Dataset EV1, etc. Legends have to be provided in a separate tab in case of .xls files. Alternatively, the legend can be supplied as a separate text file (README) and zipped together with the Table/Dataset file.

4) a .docx formatted letter INCLUDING the reviewers' reports and your detailed point-by-point responses to their comments. As part of the EMBO Press transparent editorial process, the point-by-point response is part of the Review Process File (RPF), which will be published alongside your paper.

5) a complete author checklist, which you can download from our author guidelines. Please insert information in the checklist that is also reflected in the manuscript. The completed author checklist will also be part of the RPF.

6) Please note that all corresponding authors are required to supply an ORCID ID for their name upon submission of a revised manuscript (<https://orcid.org/>), which can be linked to your profile in the manuscript submission system.

7) Before submitting your revision, primary datasets produced in this study need to be deposited in an appropriate public database. Please remember to provide a reviewer password if the datasets are not yet public. The accession numbers and database should be listed in a formal "Data Availability" section placed after Methods. Please note that the Data Availability Section is restricted to new primary data that are part of this study. * Note - All links should resolve to a page where the data can be accessed. *

If your study has not produced novel datasets, please mention this fact in the Data Availability Section.

8) At EMBO Press we ask authors to provide source data for the main manuscript figures. You will receive a separate email with instructions for providing source data with your revised manuscript, including how to upload and organize the files.

9) Our journal also encourages inclusion of *data citations in the reference list* to directly cite datasets that were re-used and obtained from public databases. Data citations in the article text are distinct from normal bibliographical citations and should directly link to the database records from which the data can be accessed. In the main text, data citations are formatted as follows: "Data ref: Smith et al, 2001" or "Data ref: NCBI Sequence Read Archive PRJNA342805, 2017". In the Reference list, data citations must be labeled with "[DATASET]". A data reference must provide the database name, accession number/identifiers and a resolvable link to the landing page from which the data can be accessed at the end of the reference.

10) Regarding data quantification, the following points must be specified in each figure legend:

- the name of the statistical test used to generate error bars and P values,

- the number (n) of independent experiments (please specify technical or biological replicates) underlying each data point,

- the nature of the bars and error bars (s.d., s.e.m.),

- If the data are obtained from n Program fragment delivered error ``Can't locate object method "less" via package "than" (perhaps you forgot to load "than"?) at //ejpvfs23/sites23b/embor_www/letters/embor_decision_revise_and_review.txt line 54.' 2, use scatter blots showing the individual data points.

Discussion of statistical methodology can be reported in the materials and methods section, but figure legends should contain a basic description of n, P and the test applied.

- Please also include scale bars in all microscopy images.

11) The journal requires a statement specifying whether or not authors have competing interests (defined as all potential or actual interests that could be perceived to influence the presentation or interpretation of an article). In case of competing interests, this must be specified in your disclosure statement.

12) All Materials and Methods need to be described in the main text using our 'Structured Methods' format, which is required for all research articles. According to this format, the Methods section includes a Reagents and Tools Table (listing key reagents, experimental models, software and relevant equipment and including their sources and relevant identifiers) followed by a Methods and Protocols section describing the methods using a step-by-step protocol format. The aim is to facilitate adoption of the methodologies across labs. More information on how to adhere to this format as well as a downloadable template (.docx) for the Reagents and Tools Table can be found in our author guidelines.

We would also welcome the submission of cover suggestions, or motifs to be used by our Graphics Illustrator in designing a cover.

As part of the EMBO publication's Transparent Editorial Process, EMBO reports publishes online a Review Process File (RPF) to accompany accepted manuscripts. This File will be published in conjunction with your paper and will include the referee reports, your point-by-point response and all pertinent correspondence relating to the manuscript.

You are able to opt out of this by letting the editorial office know (emboreports@embo.org). If you do opt out, the Review Process File link will point to the following statement: "No Review Process File is available with this article, as the authors have chosen not to make the review process public in this case."

I look forward to seeing a revised form of your manuscript when it is ready.

Yours sincerely,

Martina Rembold, PhD
Senior Editor
EMBO reports

==================

Referee #1:

Review 3rd December 2025

Title: Human ZBP1 Is a Potent Inducer of Cell Death Through Mechanisms Divergent from Mouse ZBP1

Manuscript # EMBOR-2025-63206V1

General Remarks

This study would like to show that human and mouse ZBP1 behave differently with mouse ZBP1 being less "primed" to induce cell death than human ZBP1, and human generally induces an apoptotic while mouse ZBP1 induces a necroptotic death.

Throughout the manuscript technical replicates are presented with the figure legend stating that the data are representative of 3 repeats. But, if the data are representative of 3 repeats, then the most scientifically rigorous way to report the data is to combine the 3 repeats, with the appropriate error bars etc. Currently what is shown are technical repeats and the reader has to take on faith that they are representative...

Error bars are defined but as these are technical repeats this doesn't have much value. Scale bars in micrographs were included, but only in one micrograph per panel. At a minimum each different condition should have a scale bar (e.g. Fig. 3G has 4 different "conditions" but only one scale bar. Western blots are correctly labelled with mw markers and have appropriate loading controls. Phospho-blots are controlled with the unphosphorylated protein.

Overall the authors make a reasonable case for the fact that human and mouse behave differentially but I think the idea that human and mouse are therefore expressed differently (Fig. 5) needs a lot more work. A comparison of one, recently generated, mouse cell line that is of mesenchymal origin to 5 human cell lines, of long standing and epithelial origin, doesn't allow one to claim that mouse and human expression are different. Comparing primary cells, e.g., from peripheral blood would go some way to improving the evidence for this claim, but could be further improved by looking at other lineages/types. Strangely, if one wishes to compare expression, different stimulation times were used, 24h for mouse and 48 for human.

Furthermore, this is just RNA levels, unfortunately it is not possible to compare antibody signal between mouse and human, even if the same antibody is used because the antibody may have a different affinity to one species or the other ... unless the antibody signal is quantified using defined amounts of human and mouse recombinant protein.

Fig. 5C where is the control that IFNß has upregulated anything? Why are different stimulation times used when comparing between mouse (Fig. 5B) and human (Fig. 5C)? Why weren't these cells stimulated with IFNγ?

Referee #2:

Lu et al. present a well-written and compelling manuscript that highlights key differences in ZBP1 signaling between mice and humans. Specifically, while mouse ZBP1 relies on RHIM1, human ZBP1 requires all three RHIM domains to trigger cell death. These distinctions are critical and should be carefully considered when translating findings from murine preclinical models to human systems.

The manuscript is timely and comprehensive; however, I recommend addressing the following concerns:

• Statistical Analysis: Throughout the manuscript, statistical evaluation of cell viability data is missing, making interpretation difficult. Please include appropriate statistical analyses.
• Figure 1B: The prominent band in the hZBP1 + Dox (10h) lane of the cleaved caspase-8 blot is unclear and needs clarification.
• Figures 1C, 1D, and S1K: Caspase-8 activation by ZBP1-RIPK1 requires FADD. Why, then, is caspase-8 cleaved in FADD KO cells, and why does emricasan treatment enhance cell death in these cells?
• Lines 138-140: The claim that RIPK1, RIPK3, or MLKL inhibition prevents hZBP1-induced death in FADD KO cells is not supported by the data. Please provide statistical analysis and revise the statement accordingly.
• Immunoprecipitation (Figure 1): Including blots for FADD, caspase-8, and RIPK3 in both input and IP fractions would

strengthen the interpretation of ZBP1 complex composition.
• Figure 1G and similar figures: Indicate the treatments used in the figure. Additionally, how do the authors explain ZBP1 presence in RIPK1 and RIPK3 KO cells without Dox induction?
• Line 161: The phrase "promoting necroptosis at later stages" is ambiguous. Is this speculative? Please clarify.
• Figure 2A and similar figures: The color scheme is confusing; increasing contrast may improve readability.
• Figure 4A: Since mZBP refers to mouse ZBP1 throughout the manuscript, the label "hZBP1 mZa" could be misinterpreted as human ZBP1 and mouse Za rather than human mutant Za. Consider using "mut" for mutant to avoid confusion.
• Figure 6A, 6C, and Lines 365-366: Why is the effect less pronounced in RIPK1 KO cells compared to siRNA treatment? Statistical analysis should clarify this difference, and the statement should be revised accordingly.

Referee #3:

The authors report that hZBP1 can directly recruit RIPK1 independently of RIPK3 to induce both FADD-caspase-8-dependent apoptosis and RIPK3-MLKL-dependent necroptosis, in contrast with mouse RIPK1. This was shown in a Dox-inducible overexpression system and also in the context of nuclear Z-NA accumulation induced by drug treatment or ADAR1 deficiency. While significant RIPK1 phosphorylation was observed in hZBP1-overexpressing cells, RIPK1 kinase activity was not required for hZBP1-induced apoptosis. However, in the presence of the caspase inhibitor emricisan, hZBP1-induced necroptosis was dependent on RIPK1 kinase activity, as expected. These findings reveal an interesting mechanistic divergence between human and murine ZBP1 signaling and remind us that mouse models have limitations when exploring human disease mechanisms, at least in some contexts. In general, the data are of high quality and support the authors conclusions. There are a couple of issues that require clarification.

1. It would be useful to directly compare the degree of cell death induced by human versus mouse ZBP-1 in the same cell types (i.e. HT-29, HeLa, HepG2) at the outset. The authors refer to previous studies on expression of mZBP-1 in MEFs but do not directly compare the cytotoxic effects of human versus murine ZBP-1 in the same cells to support their statements about the relative cytotoxicity of human versus murine forms of these proteins. They should also show the relative expression of human versus mouse ZBP-1 (by FLAG blot) in these experiments to ensure that differences in cytotoxicity is not simply due to the differential expression of human versus mouse ZBP-1.

2. In Fig. 1C and 1D. How does hZBP1 promote caspase-8 activation in FADD KO cells? Presumably, when hZPB1 promotes apoptosis in a RIPK1-dependent manner, hZPB1 triggers the formation of a RIPK1/FADD/CASP-8 complex to promote caspase-8 activation and apoptosis, which switches to assembly of a RIPK1/RIPK3 complex to promote MLKL phosphorylation and MLKL-dependent necroptosis when caspases are inhibited (or FADD or CASP-8 is absent). However, in Fig. 1C, addition of Emricisan (caspase-8 inhibitor) greatly enhanced cell death induced by ZBP1 overexpression in FADD KO cells. So, how does RIPK1 promote caspase-8 activation (Fig. 1D) in the absence of FADD? Are these cells not truly FADD KO?

3. Following on from the above, the complexes that form in HT-29 cells upon hZBP-1 overexpression, in the presence and absence of emricasan, should be clarified through immunoprecipitation analyses. Similar to Fig. 1E, FLAG-hZBP-1 can be immunoprecipitated (plus minus emricasan) followed by probing for RIPK1, FADD, Caspase-8 and RIPK3.

4. It would help to underscore the differences between human and mouse ZBP-1 if the authors directly compared the complexes formed by human versus mouse ZBP-1 (as described in comment 3 above) expressed in the same cell line (e.g. HT-29) for the same duration, in the presence and absence of emricasan. Once again, human versus mouse FLAG-ZBP-1 can be immunoprecipitated in the presence and absence of emricasan, followed by probing for RIPK1, FADD, Caspase-8 and RIPK3.

5. Figure 1. High power phase contrast images of the morphology of cell death observed with human and mouse ZBP1 in the presence and absence of emricasan should be provided to illustrate the differences in the cell death phenotypes.

Referee #1:

Review 3rd December 2025

Title: Human ZBP1 Is a Potent Inducer of Cell Death Through Mechanisms Divergent from Mouse ZBP1

Manuscript # EMBOR-2025-63206V1
General Remarks
This study would like to show that human and mouse ZBP1 behave differently with mouse ZBP1 being less "primed" to induce cell death than human ZBP1, and human generally induces an apoptotic while mouse ZBP1 induces a necroptotic death.

Throughout the manuscript technical replicates are presented with the figure legend stating that the data are representative of 3 repeats. But, if the data are representative of 3 repeats, then the most scientifically rigorous way to report the data is to combine the 3 repeats, with the appropriate error bars etc. Currently what is shown are technical repeats and the reader has to take on faith that they are representative.

Reply: We thank the referee for this important methodological point. We fully agree that presenting combined data from independent biological replicates is the more scientifically rigorous approach, and we apologize that the original manuscript did not reflect this standard.

In the revised manuscript, we have updated all relevant figures accordingly. Rather than showing data from a single representative experiment, we now present pooled results from at least three independent biological replicates. Error bars throughout represent the mean ± SEM. Statistical significance was determined using an unpaired parametric t-test with Welch's correction (for two-group comparisons) or one-way ANOVA followed by Tukey's post hoc test (for multiple comparisons). The significance levels are indicated as follows: ns, not significant; * $P < 0.05$; ** $P < 0.01$; *** $P < 0.001$; **** $P < 0.0001$.

Error bars are defined but as these are technical repeats this doesn't have much value. Scale bars in micrographs were included, but only in one micrograph per panel. At a minimum each different condition should have a scale bar (e.g. Fig. 3G has 4 different "conditions" but only one scale bar. Western blots are correctly labelled with mw markers and have appropriate loading controls. Phospho-blots are controlled with the unphosphorylated protein.

Reply:
Regarding error bars: This point is now addressed—as described above.
Regarding Scale Bars: We have followed this suggestion and added scale bars to each individual micrograph across all relevant figures in the revised manuscript, ensuring each experimental condition is clearly annotated. This includes DIC images, immunofluorescence confocal images, and time-lapse FRAP sequences for live-cell imaging (1C, 1H, 2F, 2K-N, 3G, 4D, EV1N, EV1O, EV3A, and EV3H-K).

We note and appreciate the referee's confirmation that Western blots and phospho-blots meet the required standards. These have been retained as in the original manuscript.

Overall the authors make a reasonable case for the fact that human and mouse behave differentially but I think the idea that human and mouse are therefore expressed differently (Fig. 5) needs a lot more work. A comparison of one, recently generated, mouse cell line that is of mesenchymal origin to 5 human cell lines, of long standing and epithelial origin, doesn't allow one to claim that mouse and human expression are different. Comparing primary cells, e.g., from peripheral blood would go some way to improving the evidence for this claim, but could be further improved by looking at other lineages/types. Strangely, if one wishes to compare expression, different stimulation times were used, 24h for mouse and 48 for human.

Reply: We agree that a rigorous cross-species comparison requires matched cell types and identical experimental conditions. To address these concerns, we have significantly expanded our cell panel and standardized our stimulation protocols as follows:

To eliminate potential bias arising from disparate cell origins, we incorporated a panel of corresponding human and murine cells. This included **matched tumor cell lines (human HT-29 vs. murine MC38, both of colonic epithelial origin)** and **primary lung fibroblasts (pLFs) from both species**. Data from these matched pairs, alongside additional human (A549, HeLa, HepG2, HaCaT) and murine (iMEFs, BMDMs) cell lines, consistently showed that hZBP1 expression is markedly lower than its murine counterparts at both mRNA and protein levels (Fig. 5A–F, EV5). Notably, despite the varying degrees of IFN pathway activation, as evidenced by the induction of classical ISGs at both the mRNA (*ISG15*, *MX1*, and *IFIT1*) and protein (ISG15 and ADAR1-p150) levels (Fig. EV5A, C), hZBP1 remained minimally responsive or exhibited markedly lower abundance across all tested human cell types compared to the robust expression of mZBP1 in murine cells (Fig. 5A–F). These comprehensive data reinforce our conclusion that hZBP1 expression is intrinsically more restricted than that of mZBP1.

We apologize for the initial discrepancy in treatment durations. To ensure consistency across species, we conducted a time-course analysis and aligned the IFNβ/IFNγ stimulation protocols. The revised manuscript now provides **direct comparisons of hZBP1 and mZBP1 induction at matched timepoints** (18 h and 36 h post-stimulation) (Fig. 5A–F, EV5).

We have incorporated the discussion into the revised manuscript (**Lines 382-412**).

Furthermore, this is just RNA levels, unfortunately it is not possible to compare antibody signal between mouse and human, even if the same antibody is used because the antibody may have a different affinity to one species or the other ... unless the antibody signal is quantified using defined amounts of human and mouse recombinant protein.

Reply: We agree with the referee that varying antibody affinities can complicate cross-species signal comparisons. Since RHIM-containing proteins—including full-length ZBP1—are difficult to purify due to their propensity to aggregate, we implemented a FLAG-tag-based normalization

strategy to calibrate the anti-ZBP1 antibody (Adipogen):

Antibody Affinity Calibration (Fig. EV5B; Fig. 5B, E): FLAG-tagged hZBP1 and mZBP1 were expressed in ZBP1 KO HeLa cells. Since the FLAG tag provides a uniform epitope across both constructs, we utilized anti-FLAG immunoblots to ensure stoichiometric comparability between hZBP1 and mZBP1 protein levels (Fig. EV5B). Probing these calibrated samples with the anti-ZBP1 antibody (Adipogen) confirmed the referee's concern: the ZBP1 antibody (Adipogen) exhibits a higher affinity for murine ZBP1 than for the human ortholog (Fig. EV5B; Fig. 5B, E).

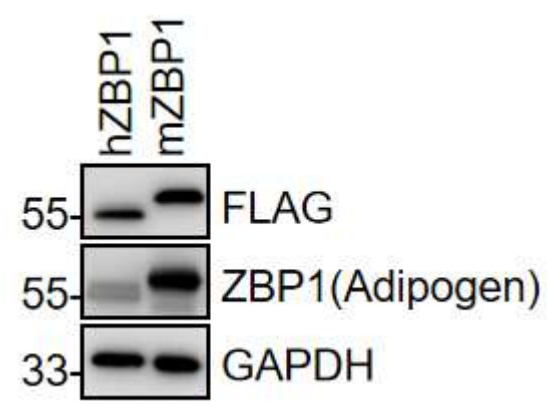

Figure EV5B. Immunoblot analysis of total lysates in ZBP1 KO HeLa cells transfected with FLAG-hZBP1 and FLAG-mZBP1, and then blot for FLAG and ZBP1 (Adipogen).

Cross-Species Normalization (Fig. 5C, F): These calibrated exogenous signals served as reference standards for endogenous comparisons. We calculated the densitometric ratio of endogenous ZBP1 relative to the species-specific exogenous reference in the same membrane. Even after normalizing for the lower affinity toward the human protein, hZBP1 levels remained undetectable or significantly lower in all tested human cells compared to the robust induction of mZBP1 in all the tested murine cells (Fig. 5B, C, E, F).

This calibration confirms that the observed disparity in protein levels reflects intrinsic biological expression rather than an artifact of differential antibody affinity.

Fig. 5C where is the control that IFNß has upregulated anything? Why are different stimulation times used when comparing between mouse (Fig. 5B) and human (Fig. 5C)? Why weren't these cells stimulated with IFNγ?

Reply: We appreciate the referee's suggestion to strengthen the conclusion of our cross-species comparisons. In the revised manuscript, we expanded our comparative analysis of hZBP1 and mZBP1 induction by **including both IFNβ and IFNγ treatments at matched timepoints (18 h and 36 h post-stimulation)** (Fig. 5A-F, EV5). To verify the efficacy of IFNβ and IFNγ treatment, we have **included the induction profiles of several representative ISGs at mRNA (e.g., *ISG15*, *MX1*, and *IFIT1*) and protein levels (e.g., ADAR1-P150 and ISG15) (Fig. EV5A, C).**

Together, these data assess the responsiveness of the tested cells to IFNβ/IFNγ stimulation while reinforcing our original conclusion and providing a more detailed picture of ZBP1 expression regulation across species.

Referee #2:

Lu et al. present a well-written and compelling manuscript that highlights key differences in ZBP1 signaling between mice and humans. Specifically, while mouse ZBP1 relies on RHIM1, human ZBP1 requires all three RHIM domains to trigger cell death. These distinctions are critical and should be carefully considered when translating findings from murine preclinical models to human systems.

The manuscript is timely and comprehensive; however, I recommend addressing the following concerns:

Reply: We thank the referee for the positive assessment of our work. We have carefully addressed all remaining comments below.

• Statistical Analysis: Throughout the manuscript, statistical evaluation of cell viability data is missing, making interpretation difficult. Please include appropriate statistical analyses.

Reply: We thank the referee for pointing out this issue. Following the referee's suggestion, in the revised manuscript, we have pooled the results from at least three independent biological replicates, with the error bars now representing the mean ± SEM. Statistical significance was determined using an unpaired parametric t-test with Welch's correction (for two-group comparisons) or one-way ANOVA followed by Tukey's post hoc test (for multiple comparisons). The significance levels are indicated as follows: ns, not significant; * $P < 0.05$; ** $P < 0.01$; *** $P < 0.001$; **** $P < 0.0001$. In the revised manuscript, we have updated all relevant figures accordingly.

• Figure 1B: The prominent band in the hZBP1 + Dox (10h) lane of the cleaved caspase-8 blot is unclear and needs clarification.

Reply: We thank the referee for pointing out this issue. The immunoblot for cleaved caspase-8 in Figure 1B has been replaced in the revised manuscript with a clearer image from a repeated experiment. The cleavage product in the hZBP1 + Dox (10 h) lane is now unambiguous, and its absence in the presence of the pan-caspase inhibitor Emricasan (Em) confirms the specificity of the signal.

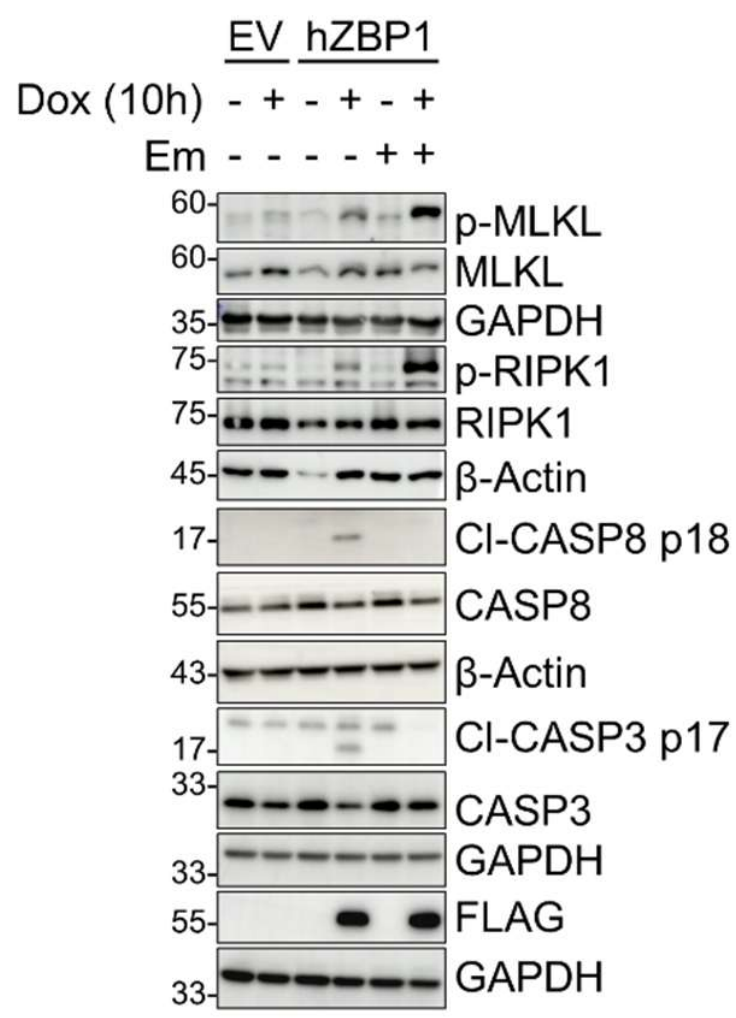

Figure 1B. Immunoblot analysis of total lysates in HT-29 expressing Dox-inducible FLAG-EV and FLAG-hZBP1 stimulated with Dox (1 μg/mL) or a combination of Dox (1 μg/mL) and emricasan (5 μM) for 10 h.

• Figures 1C, 1D, and S1K: Caspase-8 activation by ZBP1-RIPK1 requires FADD. Why, then, is caspase-8 cleaved in FADD KO cells, and why does emricasan treatment enhance cell death in these cells?

Reply: We thank the referee for these insightful questions.

1. Regarding Caspase-8 cleavage in FADD KO cells (Fig. 1E): The original immunoblot was of suboptimal quality, and we apologize for this. In the revised manuscript, we have repeated the experiments and replaced Figure 1E with a higher-resolution blot that is more representative in our experimental setting. The results clearly demonstrate that the cleavage of caspase-8 is abrogated in FADD KO cells following ZBP1 induction. This finding confirms that FADD is a critical scaffold for ZBP1-RIPK1-mediated Caspase-8 activation, consistent with the established role of the FADD-Caspase-8 complex in extrinsic apoptosis.

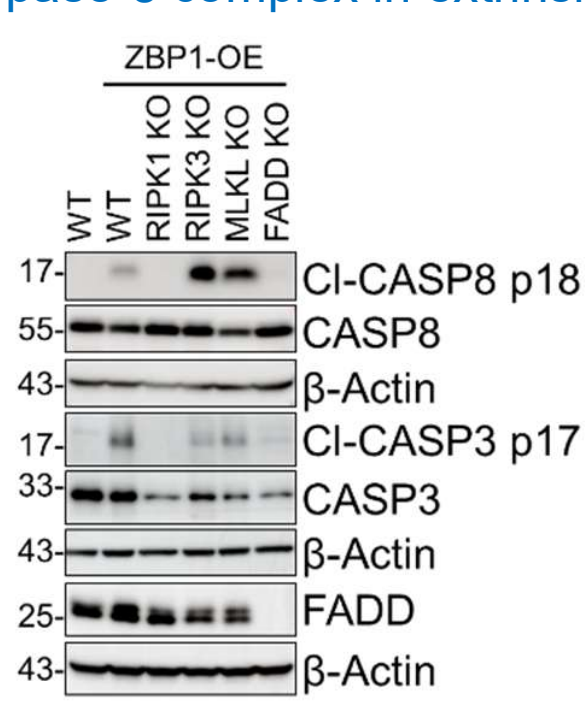

Figure 1E. Immunoblot analysis of total lysates in WT, RIPK1 KO, RIPK3 KO, MLKL KO and FADD KO HT-29 expressing Dox-inducible FLAG-hZBP1 stimulated with Dox (1 μg/mL) for 10 h.

2. Regarding why Em enhances cell death in FADD KO cells:

We have performed new experiments to confirm the result. After we pooled new data from at

least three independent biological replicates and performed rigorous statistical analyses, we found that Em mildly sensitizes FADD-KO cells to ZBP1-induced cell death (Fig. 1D and EV1L). Although the exact mechanism by which overexpressed ZBP1 activates caspase-8 without FADD remains unclear, we speculate that basal caspase-8 proteolytic activity persists — potentially at levels below the detection limit of immunoblotting. This residual activity may be responsible for restraining a small portion of necroptosis upon hZBP1 overexpression independent of FADD, thereby preventing further cell death in hZBP1-overexpressing FADD KO cells. This speculation is supported by previous genetic evidence demonstrating that mice with intestinal epithelial cell-specific deletion of FADD develop ileitis in a caspase-8-GSDMD-dependent manner when MLKL is knocked out (PMID: 32362323).

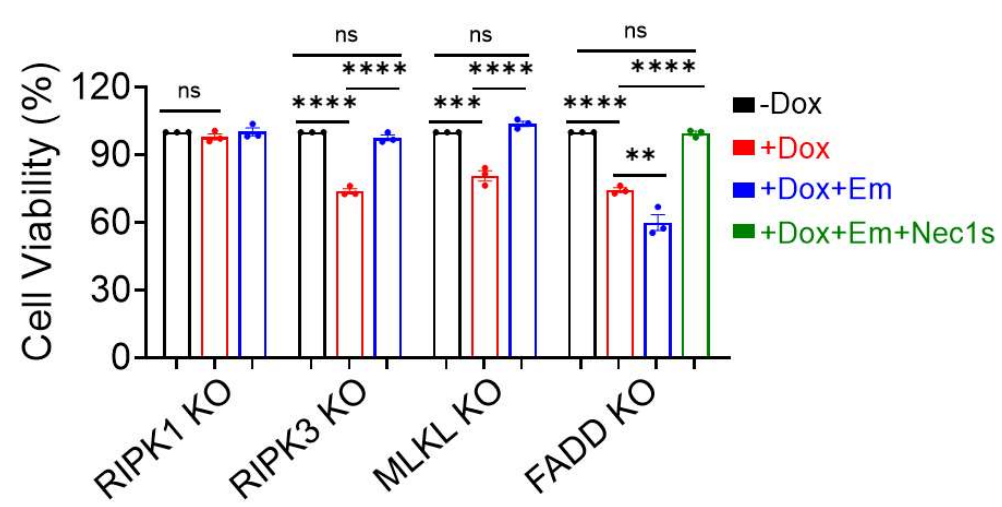

Figure 1D. Cell viability measured by neutral red uptake in RIPK1 KO, RIPK3 KO, MLKL KO, and FADD KO HT-29 expressing Dox-inducible FLAG-hZBP1 stimulated with or without Dox (1 μg/mL) or combinations of Dox and emricasan (5 μM) or Nec-1s (10 μM) for 24 h.

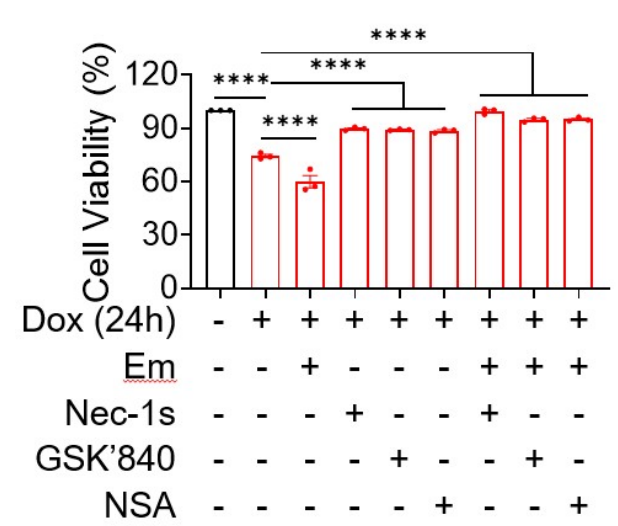

Figure EV1L. Cell viability measured by neutral red uptake in FADD KO HT-29 expressing Dox-inducible FLAG-hZBP1 stimulated with or without Dox (1 μg/mL), or combinations of Dox and emricasan (5 μM), Nec-1s (10 μM), GSK'840 (3 μM), and NSA (5 μM) for 24 h.

We speculate that this residual caspase-8 activity may exert a pro-survival role by mediating the proteolytic cleavage and inactivation of RIPK1 and RIPK3, thereby limiting the assembly of the ZBP1-recruited necrosome. Consequently, pharmacological inhibition of this residual activity by Em further facilitates the transition toward necroptosis in FADD KO cells. We have incorporated this discussion into the revised manuscript (**Lines 158-169** in the Results section and **Lines 509-515** in the Discussion section).

• Lines 138-140: The claim that RIPK1, RIPK3, or MLKL inhibition prevents hZBP1-induced death in FADD KO cells is not supported by the data. Please provide statistical analysis and revise the statement accordingly.

Reply: We thank the referee for this constructive feedback. Following the suggestion, we have pooled the results from three independent biological replicates and performed a rigorous statistical analysis (One-way ANOVA with Tukey's post-hoc test). The updated results are now presented in the revised Fig. EV1L.

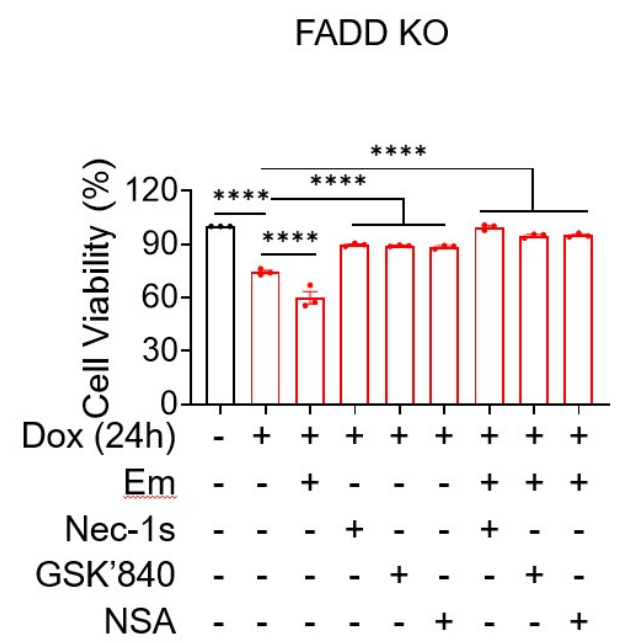

Figure EV1L. Cell viability measured by neutral red uptake in FADD KO HT-29 expressing Dox-inducible FLAG-hZBP1 stimulated with or without Dox (1 μg/mL), or combinations of Dox and emricasan (5 μM), Nec-1s (10 μM), GSK'840 (3 μM), and NSA (5 μM) for 24 h.

As the referee correctly pointed out, while pharmacological inhibition of RIPK1, RIPK3, or MLKL significantly attenuated hZBP1-induced cell death in FADD KO cells, it provided less comprehensive protection than the combination with Em. This discrepancy suggests that in the FADD-deficient context, even when necroptosis is blocked, the basal caspase-8 activity facilitates a minor portion of cell death independently of FADD. This notion is consistent with our observation that Em mildly sensitizes FADD KO cells to die. We have revised the corresponding statement in the manuscript to more accurately reflect the data: "Similar to results obtained with caspase inhibition (Fig. 1A; and EV1A), hZBP1-induced cell death in FADD KO cells was inhibited by RIPK1 kinase inhibitor Nec-1s or necroptosis inhibitors GSK'840 or NSA (Fig. 1D, E; and EV1L). A minor fraction of residual cell death in FADD KO cells was further attenuated by the addition of Em (Fig. EV1L), suggesting that the limited caspase-8 activity facilitates cell death independently of FADD. Interestingly, we observed that Em treatment mildly exacerbated cell death in FADD KO HT-29 cells (Fig. 1D; and EV1L), suggesting that, even in the absence of the FADD scaffold, basal caspase-8 proteolytic activity persists—potentially at levels below the detection limit of immunoblotting (Fig. 1E). This residual activity may exert a pro-survival role by mediating the proteolytic cleavage and inactivation of RIPK1 and RIPK3." (**Lines 158-168**)

• Immunoprecipitation (Figure 1): Including blots for FADD, caspase-8, and RIPK3 in both input and IP fractions would strengthen the interpretation of ZBP1 complex composition.

Reply: We thank the referee for this constructive suggestion. To provide a comprehensive characterization of the ZBP1-centered complex, we have performed new Co-IP assays in HT-29 cells overexpressing FLAG-hZBP1, both in the presence and absence of Emricasan (Em) (Fig. 1F).

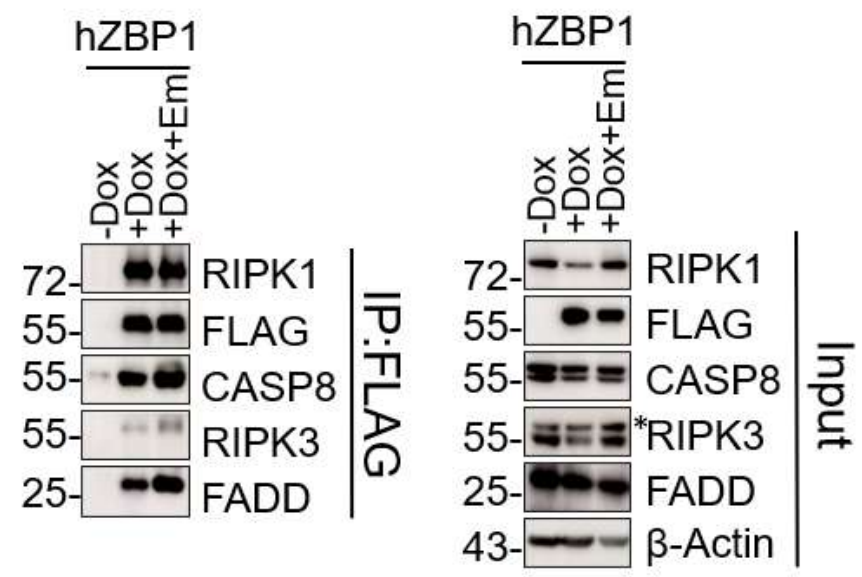

Figure 1F. Immunoblot analysis of FLAG immunoprecipitates and total lysates (Input) from HT-29 expressing Dox-inducible FLAG-tagged hZBP1 stimulated with or without Dox (1 μg/mL) or combinations of Dox (1 μg/mL) and emricasan (5 μM) for 10 h.

As suggested, we included immunoblots for RIPK1, RIPK3, FADD, and caspase-8 in both input and IP fractions. Our results demonstrate that these key cell death mediators are recruited to hZBP1 upon its induction. Notably, the association of these components with hZBP1 persists upon Emricasan pretreatment. These findings underscore the role of hZBP1 as a molecular scaffold that nucleates a multifaceted signaling complex—comprising RIPK1, RIPK3, FADD, and Caspase-8—to drive potent cell death. We have now included the following statement in the revised manuscript: "The results demonstrated that hZBP1 robustly recruits a multifaceted signaling complex comprising RIPK1, RIPK3, FADD, and caspase-8 with or without Em pretreatment (Fig. 1F)." (**Lines 174-176**)

• Figure 1G and similar figures: Indicate the treatments used in the figure. Additionally, how do the authors explain ZBP1 presence in RIPK1 and RIPK3 KO cells without Dox induction?

Reply: As suggested, we have incorporated detailed annotations to indicate the presence (+) or absence (–) of doxycycline (Dox) treatment in the revised Figures 1H, 2K–N, and EV3H-K. These labels now clearly differentiate the experimental conditions across panels.

We apologize for the lack of untreated controls in the previous version, which may have led to the misinterpretation of baseline ZBP1 levels. The two images of each genotype in the original Fig. 1G were intended to demonstrate that hZBP1 forms distinct condensates or aggregates within both the nucleus and cytoplasm upon Dox induction. The revised Fig. 1G now includes non-induced controls (–Dox), confirming the lack of leakiness in our inducible system. As expected, hZBP1 remains undetectable in the absence of Doxycycline.

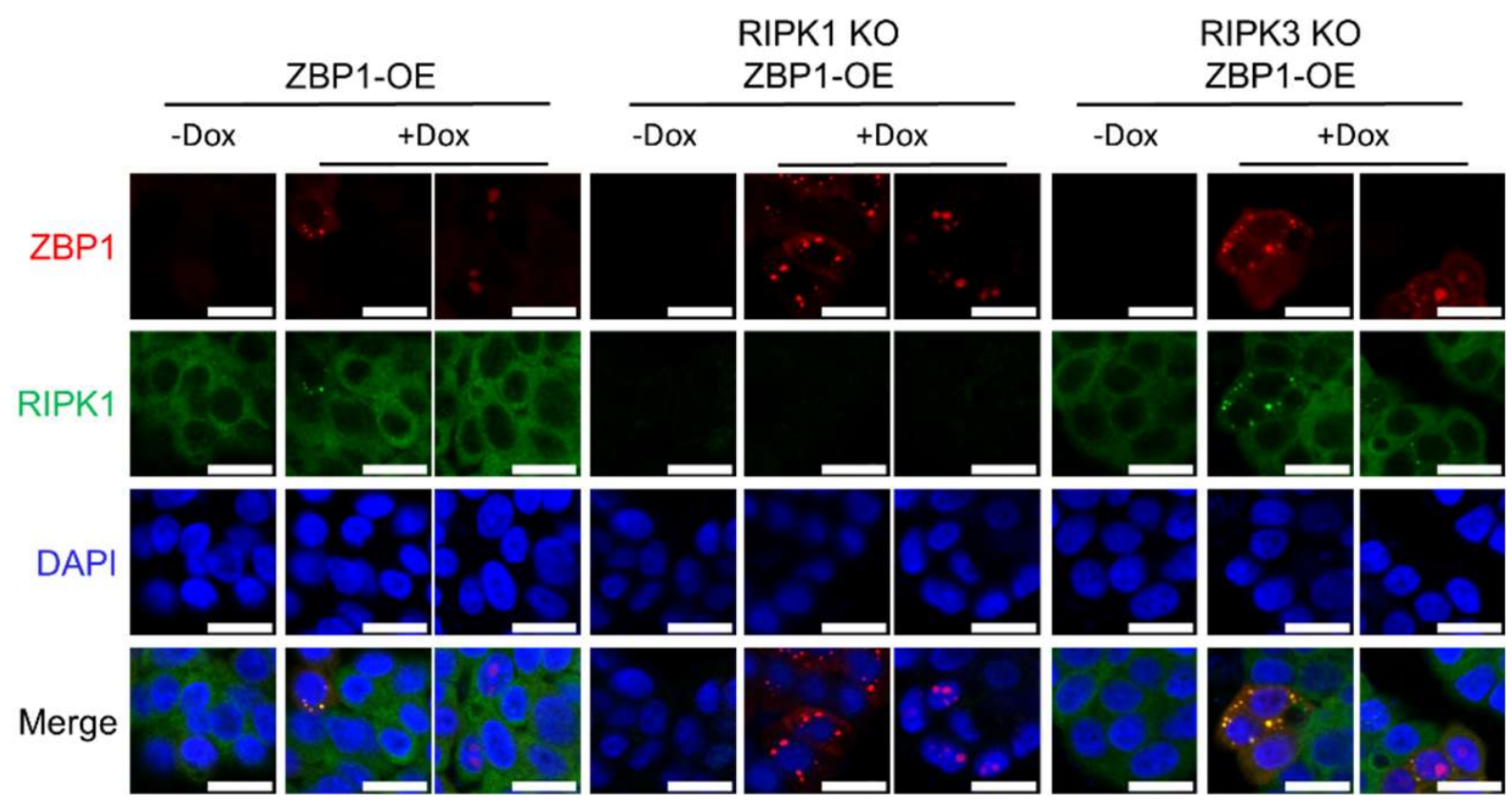

Figure 1H. Representative confocal images of WT, RIPK1 KO, and RIPK3 KO HT-29 expressing Dox-inducible FLAG-hZBP1 stimulated with or without Dox (1 μg/mL) for 10 h. Scale bars, 20 μm.

Furthermore, while our spatial analysis confirms that hZBP1 is present in both nucleus and cytoplasm upon induction, it specifically colocalizes with RIPK1 exclusively in the cytosol, which is essential for the subsequent activation of the cell death signaling complex.

• Line 161: The phrase "promoting necroptosis at later stages" is ambiguous. Is this speculative? Please clarify.

Reply: As shown in the revised Fig. 1B (right), the phosphorylation of MLKL was completely abolished upon Nec-1s treatment in hZBP1-overexpressing HT-29 cells. This demonstrates that **RIPK1 kinase activity is a prerequisite for the activation of downstream necroptotic effectors in this context**.

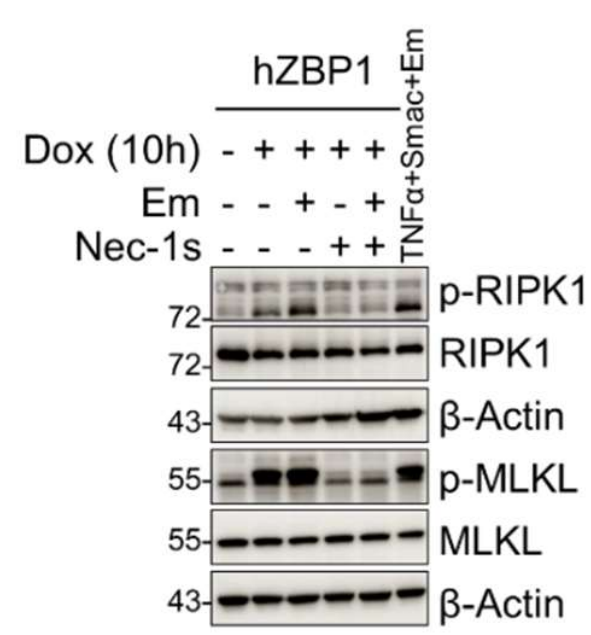

Figure 1B. Immunoblot analysis of total lysates in HT-29 expressing Dox-inducible FLAG-EV, FLAG-hZBP1 stimulated with Dox (1 μg/mL), a combination of Dox (1 μg/mL) and emricasan (5 μM) or Nec-1s (10 μM) for 10 h.

While RIPK1 kinase activity is essential for necroptosis, our data in Fig. 1A and EV1A showed that Nec-1s alone does not reverse hZBP1-induced death. This indicates that **RIPK1 also functions as a scaffold to mediate apoptosis, which can occur independently of its kinase activity**. The inability of Nec-1s to prevent cell death in Fig. 1A and EV1A likely stems from a compensatory shift from necroptosis to apoptosis upon pharmacological blockade of the RIPK1 kinase activity-dependent necroptotic pathway.

To accurately reflect the concurrent activation of the ZBP1-RIPK1-RIPK3-MLKL cascade and avoid speculative phrasing, we have revised the statement as follows: “Thus, this RIPK1 phosphorylation may facilitate the recruitment of RIPK3 to the signaling complex, thereby promoting ensuing necroptosis.” (**Lines 196-198**)

• Figure 2A and similar figures: The color scheme is confusing; increasing contrast may improve readability.

Reply: Following the suggestion, we have refined the color palette and increased the contrast across all relevant diagrams (e.g., Fig. 2A, 3A, 3M, 4A, and 5D). Specifically, we have applied distinct, high-contrast colors to differentiate the conserved domains of ZBP1 from different species.

• Figure 4A: Since mZBP1 refers to mouse ZBP1 throughout the manuscript, the label "hZBP1 mZa" could be misinterpreted as human ZBP1 and mouse Za rather than human mutant Za. Consider using "mut" for mutant to avoid confusion.

Reply: We thank the referee for the helpful suggestion regarding the nomenclature. We have revised the labeling throughout the manuscript and figures. In the revised manuscript, the prefix "m" is now reserved exclusively for "mouse" (e.g., mZBP1), while "mut" is used consistently to denote "mutant". Specifically, we have replaced the term "mZα" (where it referred to the mutant domain) with "mutZα".

• Figure 6A, 6C, and Lines 365-366: Why is the effect less pronounced in RIPK1 KO cells compared to siRNA treatment? Statistical analysis should clarify this difference, and the statement should be revised accordingly.

Reply: We thank the referee for the careful evaluation and for pointing out the differences in the RIPK1-dependent effects. We have addressed these concerns as follows:

First, we have pooled the results from at least three independent biological replicates and performed a rigorous statistical analysis (One-way ANOVA with Tukey’s post-hoc test). The updated *P* values are now indicated in the revised Figures 6A and 6C. The significance levels are indicated as follows: ns, not significant; * $P < 0.05$; ** $P < 0.01$; *** $P < 0.001$; **** $P < 0.0001$.

However, the observed differences between Figures 6A and 6C primarily stem from **the use of distinct cell models: HT-29 (Fig. 6A) and HeLa (Fig. 6C)**. Our data suggest that HT-29 cells require a more potent stimulus (IFNβ priming combined with CBL/Plad-B) to trigger ZBP1-mediated death, whereas HeLa cells exhibit a higher baseline sensitivity to the ZBP1-RIPK1 axis in response to IFNβ priming alone without additional stimuli. Despite variations in the extent of rescue, RIPK1 deficiency consistently abrogates this mode of ZBP1-mediated cell death across different cellular contexts. We have added the following comments in the revised manuscript: “Notably, unlike the requirements observed in HT-29 cells, IFNβ treatment alone

was sufficient to trigger significant ZBP1- and RIPK1-dependent cell death in HeLa cells." (**Lines 446-448**)

As the referee noted, **RIPK1 deficiency in HeLa cells (Fig. 6B) was achieved using a bulk pool of sgRNA-targeted cells rather than a single-cell clone**. Although the use of four independent sgRNAs did not result in a complete ablation of protein across the entire population, we selected cell pools with high editing efficiency (sg*RIPK1*-2 and sg*RIPK1*-3) for our assays. We confirmed that the substantial reduction in RIPK1 protein levels was sufficient to yield a statistically robust rescue of cell death—comparable to the effect of ZBP1 KO—following CBL and Plad-B treatment. These results demonstrate that even partial depletion of the RIPK1 pool significantly abrogates ZBP1-mediated cell death.

In Figure 6A, **we also evaluated RIPK3 KO and MLKL KO HT-29 cells; however, deficiency of RIPK3 or MLKL did not rescue cell death as effectively as ZBP1 or RIPK1 deficiency.** We speculate that this partial protection may arise because endogenous ZBP1-driven lethality is predominantly mediated by apoptosis in this context. Alternatively, it is possible that inhibiting the necroptotic machinery triggers a compensatory transition to apoptosis, a phenomenon previously characterized in various cell death models. We have revised the following comments in the manuscript: "In contrast, deficiency of RIPK3 or MLKL showed modest or no inhibitory effect, suggesting that hZBP1 activation by CBL or Plad-B can trigger RIPK1-dependent apoptosis in these necroptosis-deficient HT-29 cells." (**Lines 440-442**)

Referee #3:

The authors report that hZBP1 can directly recruit RIPK1 independently of RIPK3 to induce both FADD-caspase-8-dependent apoptosis and RIPK3-MLKL-dependent necroptosis, in contrast with mouse RIPK1. This was shown in a Dox-inducible overexpression system and also in the context of nuclear Z-NA accumulation induced by drug treatment or ADAR1 deficiency. While significant RIPK1 phosphorylation was observed in hZBP1-overexpressing cells, RIPK1 kinase activity was not required for hZBP1-induced apoptosis. However, in the presence of the caspase inhibitor emricisan, hZBP1-induced necroptosis was dependent on RIPK1 kinase activity, as expected. These findings reveal an interesting mechanistic divergence between human and murine ZBP1 signaling and remind us that mouse models have limitations when exploring human disease mechanisms, at least in some contexts. In general, the data are of high quality and support the authors conclusions. There are a couple of issues that require clarification.

Reply: We thank the referee for their positive assessment of our work. We have carefully addressed all remaining comments below.

1. It would be useful to directly compare the degree of cell death induced by human versus mouse ZBP-1 in the same cell types (i.e. HT-29, HeLa, HepG2) at the outset. The authors refer to previous studies on expression of mZBP-1 in MEFs but do not directly compare the cytotoxic effects of human versus murine ZBP-1 in the same cells to support their statements about the

relative cytotoxicity of human versus murine forms of these proteins. They should also show the relative expression of human versus mouse ZBP-1 (by FLAG blot) in these experiments to ensure that differences in cytotoxicity is not simply due to the differential expression of human versus mouse ZBP-1.

Reply: We thank the referee for raising this point, and we apologize for any lack of clarity in our initial submission that may have led to this misunderstanding. In our original manuscript, we had already performed a direct comparison of hZBP1, mZBP1, and two chimeric variants (ZBP1(m+h) and ZBP1(h+m)) in both HT-29 and iMEF cells (Fig. 2A–E). To further support these findings and address the referee's concerns, we have now extended this analysis to HeLa and HepG2 cells in the revised study (Fig. EV3B-E). These additional results are consistent with our initial observations (Fig. EV3B-E), confirming that the heightened cytotoxicity of hZBP1 is a robust phenotype across multiple cell lineages.

We validated the relative expression of these FLAG-tagged proteins in these cells. Although hZBP1 and the ZBP1(m+h) chimera exhibited consistently lower expression levels compared to mZBP1 and ZBP1(h+m) (Fig. 2B, D; Fig. EV3B, D), they nonetheless triggered potent cell death in all tested cell lines. In stark contrast, mZBP1 and the ZBP1(h+m) chimera failed to induce significant cytotoxicity despite their higher expression levels (Fig. 2C, E; Fig. EV3C, E).

These findings provide compelling evidence that hZBP1 possesses an intrinsically superior capacity to activate cell death pathways compared to mZBP1. Furthermore, our domain-swapping experiments demonstrate that this heightened cytotoxicity is specifically endowed by the C-terminal RHIM-containing region of hZBP1.

2. In Fig. 1C and 1D. How does hZBP1 promote caspase-8 activation in FADD KO cells? Presumably, when hZBP1 promotes apoptosis in a RIPK1-dependent manner, hZBP1 triggers the formation of a RIPK1/FADD/CASP-8 complex to promote caspase-8 activation and apoptosis, which switches to assembly of a RIPK1/RIPK3 complex to promote MLKL phosphorylation and MLKL-dependent necroptosis when caspases are inhibited (or FADD or CASP-8 is absent). However, in Fig. 1C, addition of Emricisan (caspase-8 inhibitor) greatly enhanced cell death induced by ZBP1 overexpression in FADD KO cells. So, how does RIPK1 promote caspase-8 activation (Fig. 1D) in the absence of FADD? Are these cells not truly FADD KO?

Reply: We thank the referee for these insightful questions.

1. Regarding Caspase-8 cleavage in FADD KO cells (Fig. 1E): The original immunoblot was of suboptimal quality, and we apologize for this. In the revised manuscript, we have repeated the experiments and replaced Figure 1E with a higher-resolution blot that is more representative in our experimental setting. **Loss of FADD expression was confirmed in the FADD-KO cells, validating the successful depletion of the target gene**. The results clearly demonstrate that the cleavage of caspase-8 is abrogated in FADD KO cells following ZBP1 induction. This finding confirms that FADD is a critical scaffold for ZBP1-RIPK1-mediated Caspase-8 activation,

consistent with the established role of the FADD-Caspase-8 complex in extrinsic apoptosis.

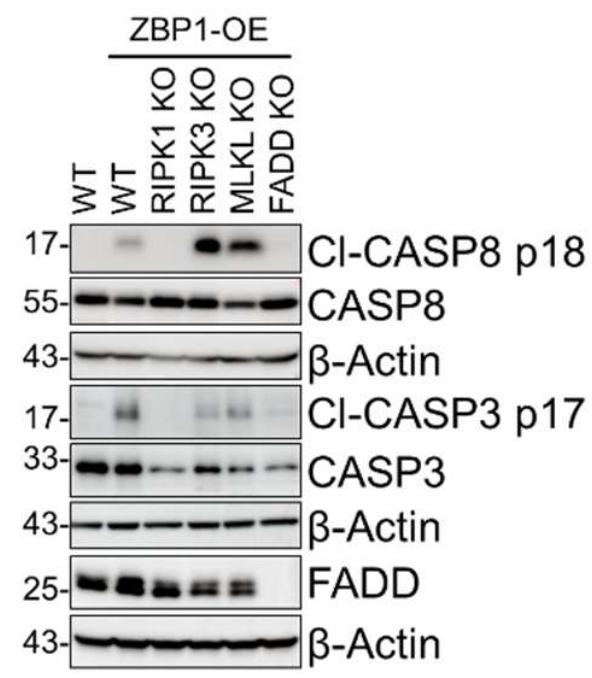

Figure 1E. Immunoblot analysis of total lysates in WT, RIPK1 KO, RIPK3 KO, MLKL KO and FADD KO HT-29 expressing Dox-inducible FLAG-hZBP1 stimulated with Dox (1 μg/mL) for 10 h.

2. Regarding why Em enhances cell death in FADD KO cells:

We have performed new experiments to confirm the result. After we pooled new data from at least three independent biological replicates and performed rigorous statistical analyses, we found that Em mildly sensitizes FADD-KO cells to ZBP1-induced cell death (Fig. 1D and EV1L). Although the exact mechanism by which overexpressed ZBP1 activates caspase-8 without FADD remains unclear, we speculate that basal caspase-8 proteolytic activity persists—potentially at levels below the detection limit of immunoblotting. This residual activity may be responsible for restraining a small portion of necroptosis upon hZBP1 overexpression independent of FADD, thereby preventing further cell death in hZBP1-overexpressing FADD KO cells. This speculation is supported by previous genetic evidence demonstrating that mice with intestinal epithelial cell-specific deletion of FADD develop ileitis in a caspase-8-GSDMD-dependent manner when MLKL is knocked out (PMID: 32362323).

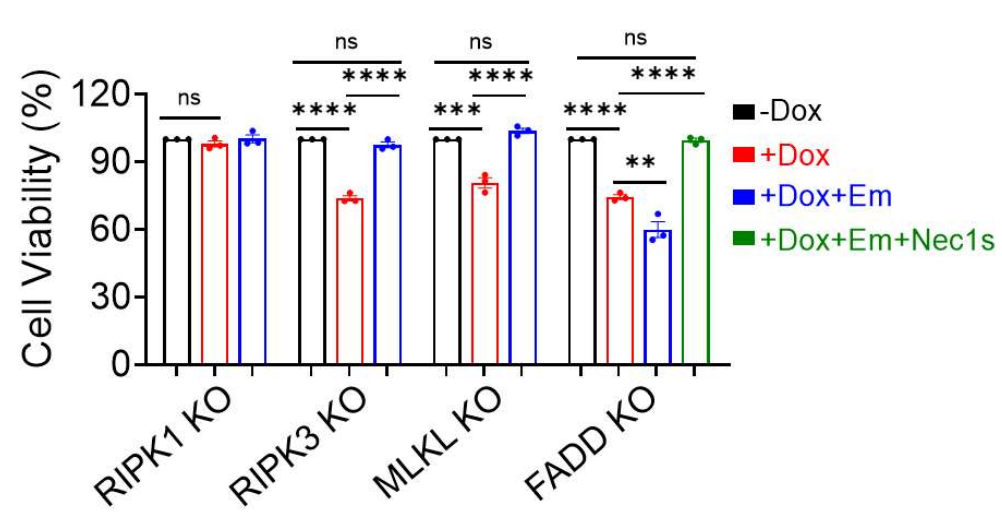

Figure 1D. Cell viability measured by neutral red uptake in RIPK1 KO, RIPK3 KO, MLKL KO, and FADD KO HT-29 expressing Dox-inducible FLAG-hZBP1 stimulated with or without Dox (1 μg/mL) or combinations of Dox and emricasan (5 μM) or Nec-1s (10 μM) for 24 h.

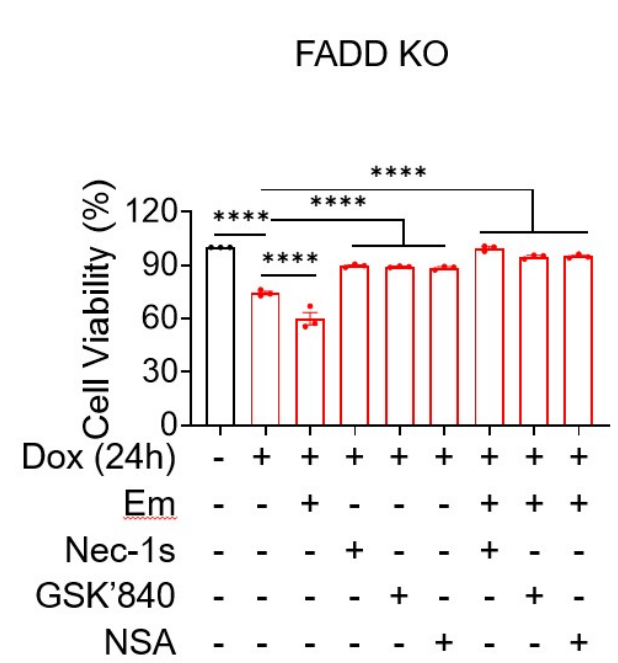

Figure EV1L. Cell viability measured by neutral red uptake in FADD KO HT-29 expressing Dox-inducible FLAG-hZBP1 stimulated with or without Dox (1 μg/mL), or combinations of Dox and emricasan (5 μM), Nec-1s (10 μM), GSK'840 (3 μM), and NSA (5 μM) for 24 h.

We speculate that this residual caspase-8 activity may exert a pro-survival role by mediating the proteolytic cleavage and inactivation of RIPK1 and RIPK3, thereby limiting the assembly of the ZBP1-recruited necrosome. Consequently, pharmacological inhibition of this residual activity by Em further facilitates the transition toward necroptosis in FADD KO cells. We have incorporated this discussion into the revised manuscript (**Lines 158-169** in the Results section and **Lines 509-515** in the Discussion section).

3. Following on from the above, the complexes that form in HT-29 cells upon hZBP-1 overexpression, in the presence and absence of emricasan, should be clarified through immunoprecipitation analyses. Similar to Fig. 1E, FLAG-hZBP-1 can be immunoprecipitated (plus minus emricasan) followed by probing for RIPK1, FADD, Caspase-8 and RIPK3.

Reply: We thank the referee for this constructive suggestion. To provide a comprehensive characterization of the ZBP1-centered complex, we have performed new Co-IP assays in HT-29 cells overexpressing FLAG-hZBP1, both in the presence and absence of Emricasan (Em) (Fig. 1F).

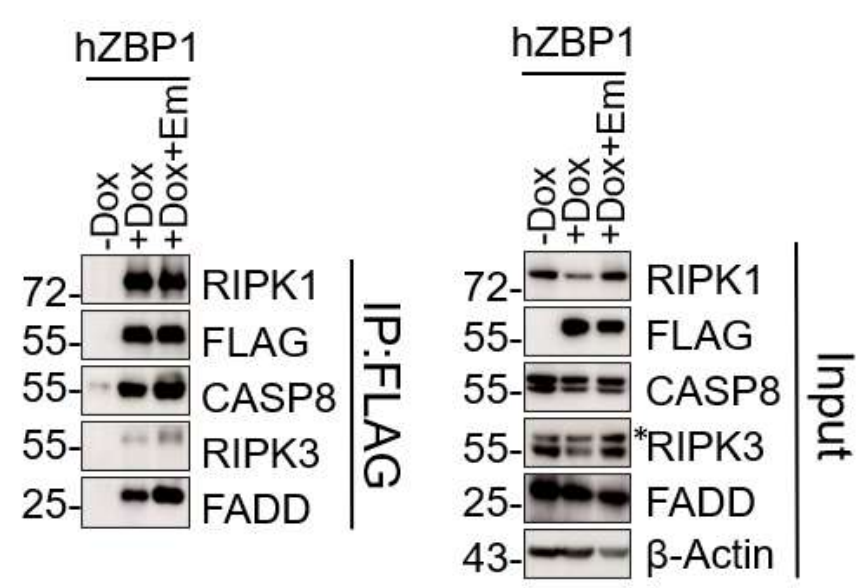

Figure 1F. Immunoblot analysis of FLAG immunoprecipitates and total lysates (Input) from HT-29 expressing Dox-inducible FLAG-tagged hZBP1 stimulated with or without Dox (1 μg/mL) or combinations of Dox (1 μg/mL) and emricasan (5 μM) for 10 h.

As suggested, we included immunoblots for RIPK1, RIPK3, FADD, and Caspase-8 in both input and IP fractions. Our results demonstrate that these key cell death mediators are recruited to hZBP1 upon its induction. Notably, the association of these components with hZBP1 persists upon Emricasan pretreatment. These findings underscore the role of hZBP1 as a molecular scaffold that nucleates a multifaceted signaling complex—comprising RIPK1, RIPK3, FADD, and Caspase-8—to drive potent cell death. We have now included the following statement in the revised manuscript: "The results demonstrated that hZBP1 robustly recruits a multifaceted signaling complex comprising RIPK1, RIPK3, FADD, and caspase-8 with or without Em pretreatment." (**Lines 174-176**)

4. It would help to underscore the differences between human and mouse ZBP-1 if the authors directly compared the complexes formed by human versus mouse ZBP-1 (as described in

comment 3 above) expressed in the same cell line (e.g. HT-29) for the same duration, in the presence and absence of emricasan. Once again, human versus mouse FLAG-ZBP-1 can be immunoprecipitated in the presence and absence of emricasan, followed by probing for RIPK1, FADD, Caspase-8 and RIPK3.

Reply: We thank the referee for this specific and constructive suggestion. To directly underscore the functional divergence between human and murine ZBP1, we performed additional Co-IP comparison within the same cellular context.

In the revised Figure 2G, we simultaneously analyzed the complex composition in HT-29 cells expressing either FLAG-hZBP1 or FLAG-mZBP1 for the same duration (10 h), both in the presence and absence of Emricasan (Em). Our results clearly demonstrate that hZBP1 robustly recruits a multifaceted complex comprising RIPK1, RIPK3, FADD, and Caspase-8, which persists upon Emricasan pretreatment.

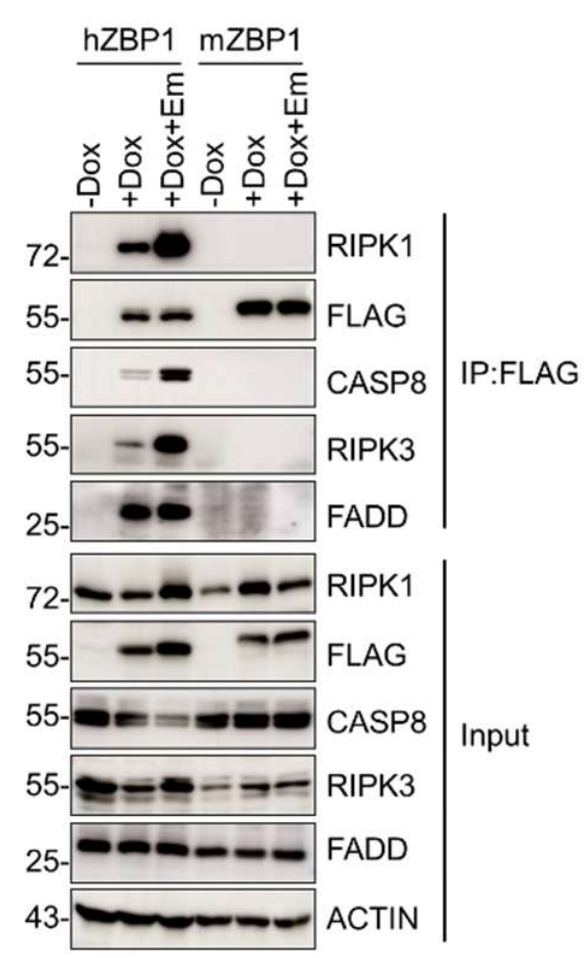

Figure 2G. Immunoblot analysis of FLAG immunoprecipitates and total lysates (Input) from HT-29 expressing Dox-inducible FLAG-tagged hZBP1 or mZBP1 stimulated with or without Dox (1 μg/mL) or combinations of Dox (1 μg/mL) and emricasan (5 μM) for 10 h.

Strikingly, and as expected from our cytotoxicity data, none of these key signaling components were recruited to mZBP1, regardless of Emricasan treatment. This sharp contrast in complex assembly provides biochemical evidence for the intrinsically heightened capacity of hZBP1 to nucleate the cell death machinery, a feature notably absent in its murine counterpart in this human cell line. We have added related discussion in the revised manuscript (**Lines 231-237**).

5. Figure 1. High power phase contrast images of the morphology of cell death observed with human and mouse ZBP1 in the presence and absence of emricasan should be provided to illustrate the differences in the cell death phenotypes.

Reply: We thank the referee for this constructive suggestion. To clearly illustrate the distinct cell death phenotypes, we have provided high-resolution Differential Interference Contrast (DIC) micrographs of both HT-29 and immortalized MEF (iMEFs) cells expressing hZBP1 or mZBP1

in the presence or absence of Emricasan (Fig. 1C, 2F, and EV3A).

In HT-29 cells, hZBP1 induction triggered a complex spectrum of morphological features. Specifically, we observed apoptotic signatures, characterized by extensive membrane blistering and the formation of apoptotic bodies, which were largely abolished by Emricasan (Em) treatment (Fig. 1C). Simultaneously, necroptotic morphology, including marked cell swelling and plasma membrane rupture, was evident and significantly exacerbated upon caspase inhibition by Em (Fig. 1C). Furthermore, a subset of cells displayed pyroptotic features, such as cell enlargement and the development of translucent, vesicular blebs, consistent with our observation of GSDMD cleavage, which was sensitive to Em treatment (Fig. EV1B). We have included the discussion in the revised manuscript (**Lines 130-139**).

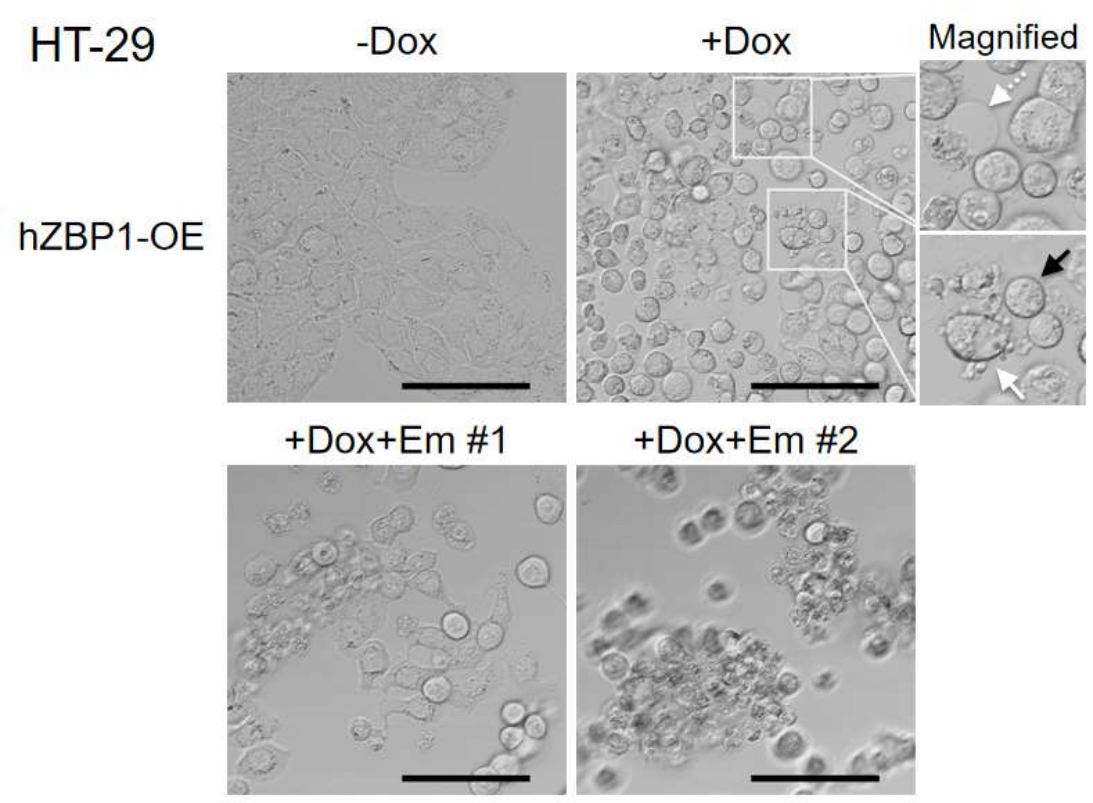

Figure 1C. Representative cell images of HT-29 expressing Dox-inducible FLAG-hZBP1 stimulated with or without Dox (1 μg/mL) or combinations of Dox (1 μg/mL) and emricasan (5 μM) for 10 h. Representative cells in white square boxes were magnified; the white solid arrow indicates the apoptotic cell, the black solid arrow indicates the necroptotic cell, and the white dotted arrow indicates the pyroptotic-like cell. Representative views of adherent cells and suspension cells in Dox+Em group were displayed and were annotated as "#1" and "#2" respectively. Scale bars, 100 μm.

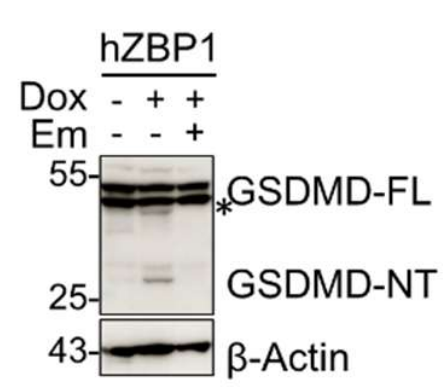

Figure EV1B. Immunoblot analysis of total lysates in HT-29 expressing Dox-inducible FLAG-hZBP1 stimulated with or without Dox (1 μg/mL) or combinations of Dox (1 μg/mL) and emricasan (5 μM) for 10 h. * non-specific band.

In stark contrast, mZBP1 overexpression failed to induce any observable cell death or morphological changes in either HT-29 or iMEF cells under basal conditions (Fig. EV3A). Notably, hZBP1 consistently triggered robust, spontaneous cell death even in cells of murine origin (Fig. 2F). In iMEFs, hZBP1-induced death manifested as a mixture of apoptotic and necroptotic phenotypes, which shifted toward an exclusive and overt necroptotic morphology (cell swelling and plasma membrane rupture) upon Em treatment. This phenotype closely

resembles the necroptosis observed during mZBP1-induced death in iMEFs only when specifically stimulated and treated with Em (Fig. EV3A). We have included the discussion in the revised manuscript (**Lines 218-227**).

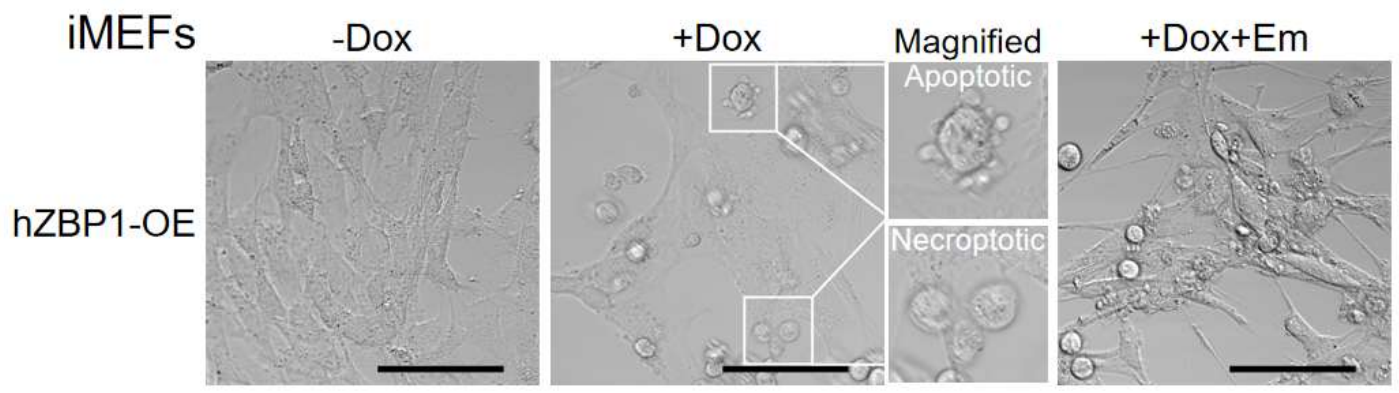

Figure 2F. Representative cell images of immortalized MEFs expressing Dox-inducible FLAG-hZBP1 stimulated with or without Dox (1 μg/mL) or combinations of Dox (1 μg/mL) and emricasan (5 μM) for 10 h. A representative apoptotic cell and necroptotic cell in white square boxes were magnified. Scale bars, 100 μm.

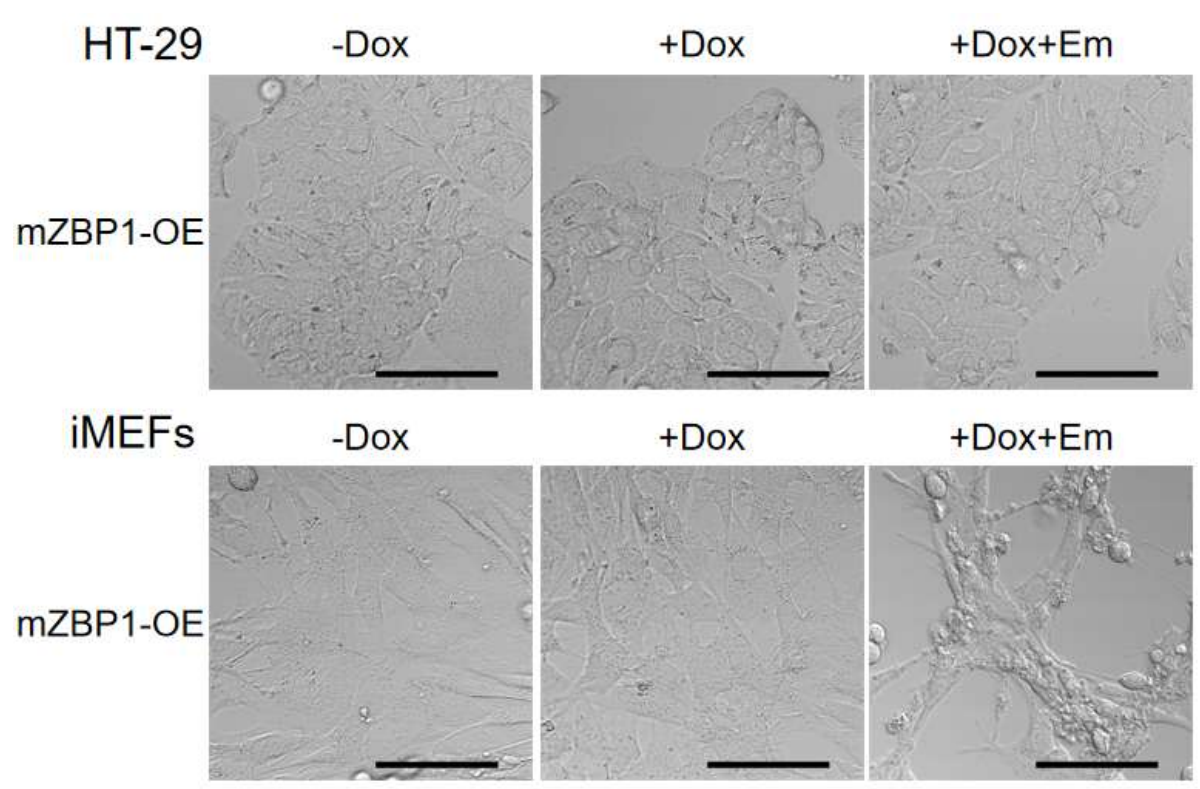

Figure EV3A. Representative cell images of HT-29 cells expressing Dox-inducible FLAG-mZBP1 (top) and immortalized MEFs expressing Dox-inducible FLAG-mZBP1 (bottom) stimulated with or without Dox (1 μg/mL) or combinations of Dox (1 μg/mL) and emricasan (5 μM) for 10 h. Scale bars, 100 μm.

Collectively, these morphological data provide compelling evidence that hZBP1, unlike its murine counterpart, intrinsically orchestrates spontaneous regulated apoptosis and necroptosis across species barriers. Upon pharmacological inhibition of caspases by Emricasan, the cell death program shifts away from apoptosis, causing necroptosis to persist and significantly exacerbate.

1st Revision - Editorial Decision 22nd May 2026

Dear Huipeng,

Thank you for the submission of your revised manuscript to EMBO reports. As you know, we have meanwhile received the full set of referee reports (copied again below). All referees are very positive about your study and recommend publication.

I am now following up with the official decision letter. Before I can accept the manuscript, I need you to address some editorial points:

- We perform a routine check on all source data reporting quantification data. In the course of doing so, I noticed potential duplications in two files, Figure EV5A and Figure EV3C. I attach a color coded file. In Figure EV5A column D and G as well as column E and H have the same numbers, respectively (samples MX1 and IFIT1). Similar findings apply to Columns R&S, U&V. Please carefully check these numbers and please provide a point-by-point response.
For Figure EV3C, there are also a few numbers that appear duplicated (e.g., the values for EV and mZBP1.

- A spot check of the source data showed that the images provided for Figure 1H, RIPK1 KO,ZBP1-OE +Dox#1 consist of only 1 pixel, at least if opened in Fiji. Only Channel 1 is a proper image.

- Regarding the Author Contributions, we use CRediT to specify the contributions of each author in the journal submission system. CRediT replaces the author contribution section, which therefore needs to be removed from the manuscript text. You can use the free text box in our system if you wish to provide more detailed descriptions. See also guide to authors https://www.embopress.org/page/journal/14693178/authorguide#authorshipguidelines.

- References: et al needs to be used after 10 author names.

- Please provide a complete author checklist, which you can download from our author guidelines. Please insert information in the checklist that is also reflected in the manuscript. The completed author checklist will also be part of the Peer Review File.

- In this context I note that you performed experiments using mice. Please fill the relevant sections in the Author checklist and provide information on housing and husbandry conditions in the checklist and the methods section. Please also state details of authority granting ethics approval (IRB or equivalent committee(s) and provide the reference number for approval. A statement of compliance with ethical regulations needs also to be included.

- The information on funding in the manuscript text and the online submission system must be congruent. The following funder(s) appear to be missing from the system - the startup fund from the Life Sciences Institute of Zhejiang University and the Fundamental Research Funds for the Central Universities (226-2025-00126).

- The Funding section heading should be remvoed from the manuscript, as this info should go under Acknowledgments.

- You have uploaded a .pptx file called "Supplementary-WB.pptx". I assume that these are source data used to generate figures? Since you have provided source data for all figures separately, this file appears redundant and can be deleted.

- The Methods section will include a Reagents and Tools Table (listing key reagents, experimental models, software and relevant equipment and including their sources and relevant identifiers). Please download and fill our template (.docx) for the Reagents and Tools Table, which can be found in our author guidelines. The Reagents and Tools Table file must be uploaded as a separate file and should not be part of the methods section of the manuscript. The table will be inserted by the typesetters.

- The manuscript sections should be in the following order: Title page - Abstract & Keywords - Introduction - Results - Discussion - Methods - Data Availability - Acknowledgments - Disclosure Statement & Competing Interests - References - Figure Legends - (Main Tables with legends if applicable) - Expanded View Figure Legends.

- Please address the following points in the figure legends and please track or mark the changes:
* Please note that the exact p values are not provided in the legends of figures 1A, D; 2C, E, H; 3B, F, O, Q; 4B, 5A, C, H, I; 6A, C, D, G, I; EV1 A, D, F, G, L; EV2 C, D; EV3 C, E, F, G; EV4 G, H; EV5 A
* Please note that the error bars are not defined in the legends of figures 1A, D; 2C, E, H; 3B, F, O, Q; 4B, 5A, C, F, H, I; 6A, C, D, G, I; EV1A, D, F, G,J, L, L, N, O; EV2 C, D; EV3 C, E, F, G; EV4G, H; EV5 A

- Thank you for alerting us to the qPCR data being based on n=2 in Figure EV5. Please update the figure and source data to include the results from the third replicate, as suggested.

- As a standard procedure we edit title and abstract. Please find my suggestion with minor modifications below my signature.

- Finally, EMBO Reports papers are accompanied online by

A) a short (1-2 sentences) summary of the findings and their significance,
B) 2-3 bullet points highlighting key results and
C) a schematic summary figure that provides a sketch of the major findings (not a data image).
Please provide the summary figure as a separate file in PNG or JPG format at a size of 550x300 pixels (width x height). Please note that the size is rather small and that text needs to be readable at the final size. Please send us this information along with the revised manuscript.

We look forward to seeing a final version of your manuscript as soon as possible.

With kind regards,

Martina

Martina Rembold, PhD
Senior Editor
EMBO reports

================

Referee #1:

The authors addressed my concerns well.

Referee #2:

The authors have appropriately addressed my initial concerns/recommendations.

Referee #3:

The authors have comprehensively dealt with all of my previous comments.

================

Human ZBP1 Is a Potent Inducer of Cell Death Through Mechanisms Divergent from Mouse ZBP1

Z-DNA-binding protein 1 (ZBP1) senses Z-form nucleic acids to trigger cell death and inflammation via RHIM domain-mediated interactions with RIPK1 and RIPK3. Here we show that compared to mouse ZBP1 (mZBP1), human ZBP1 (hZBP1) possesses a heightened sensitivity for inducing cell death in cells across different species and potent tumor-killing effects in vivo. In contrast to mZBP1, which signals primarily through RIPK3, hZBP1-induced cell death depends on RIPK1 in a RIPK3-independent manner. Specifically, while the scaffold function of RIPK1 is required for hZBP1-mediated apoptosis, its kinase activity is indispensable for the execution of necroptosis. Unlike mZBP1, which primarily uses only its RHIM1 domain, hZBP1 requires all three RHIM domains (RHIM1, RHIM2, and RHIM3) to trigger cell death. We further identify the C-terminal RHIM2 and RHIM3 regions as the key determinant that confers hZBP1 high sensitivity and confirm that endogenous hZBP1 promotes RIPK1-dependent cell death under pathological conditions. Together, our findings reveal an intrinsic mechanistic divergence between human and murine ZBP1 signaling and highlight the limitations of translating preclinical findings in animal models to human therapeutic strategies targeting this pathway.

All minor editorial requests have been addressed by the authors.

2nd Revision - Editorial Decision 12th Jun 2026

Dr. Huipeng Jiao
Zhejiang Key Laboratory of Molecular Cancer Biology, Zhejiang University
Life Sciences Institute
China

Dear Huipeng,

Thank you for implementing the final changes and clarifications. I am very pleased to accept your manuscript for publication in the next available issue of EMBO reports. Thank you for your contribution to our journal.

Your manuscript will be processed for publication by EMBO Press. It will be copy edited and you will receive page proofs prior to publication. Please note that you will be contacted by Springer Nature Author Services to complete licensing and payment information.

You may qualify for financial assistance for your publication charges - either via a Springer Nature fully open access agreement or an EMBO initiative. Check your eligibility: https://link.springer.com/journal/44319/how-to-publish-with-us

Should you be planning a Press Release on your article, please notify the production team via this form in order to coordinate publication and release dates: https://forms.cloud.microsoft/e/eH4A8JVP6g

If you have any questions, please do not hesitate to contact the Editorial Office. Thank you for your contribution to EMBO Reports.

Kind regards,

Martina

Martina Rembold, PhD
Senior Editor
EMBO reports

>>> Please note that it is EMBO Reports policy for the transcript of the editorial process (containing referee reports and your response letter) to be published as an online supplement to each paper. If you do NOT want this, you will need to inform the Editorial Office via email immediately. More information is available here: https://link.springer.com/partners/embo-press/editorial-policies#Peer%20review

EMBO Press Author Checklist

Corresponding Author Name: Huipeng Jiao, Juan Lin, Jiafa Tou	
Journal Submitted to: EMBO Reports	
Manuscript Number: EMBOR-2025-63206	

USEFUL LINKS FOR COMPLETING THIS FORM
The EMBO Journal - Author Guidelines
EMBO Reports - Author Guidelines
Molecular Systems Biology - Author Guidelines
EMBO Molecular Medicine - Author Guidelines

Reporting Checklist for Life Science Articles (updated January
This checklist is adapted from Materials Design Analysis Reporting (MDAR) Checklist for Authors. MDAR establishes a minimum set of requirements in transparent reporting in the life sciences (see Statement of Task: 10.31222/osf.io/9sm4x). Please follow the journal's guidelines in preparing your
Please note that a copy of this checklist will be published alongside your article.

Abridged guidelines for figures
1. Data
The data shown in figures should satisfy the following conditions:
- the data were obtained and processed according to the field's best practice and are presented to reflect the results of the experiments in an accurate and unbiased manner.
- ideally, figure panels should include only measurements that are directly comparable to each other and obtained with the same assay.
- plots include clearly labeled error bars for independent experiments and sample sizes. Unless justified, error bars should not be shown for technical
- if n<5, the individual data points from each experiment should be plotted. Any statistical test employed should be justified.
- Source Data should be included to report the data underlying figures according to the guidelines set out in the authorship guidelines on Data

2. Captions
Each figure caption should contain the following information, for each panel where they are relevant:
- a specification of the experimental system investigated (eg cell line, species name).
- the assay(s) and method(s) used to carry out the reported observations and measurements.
- an explicit mention of the biological and chemical entity(ies) that are being measured.
- an explicit mention of the biological and chemical entity(ies) that are altered/varied/perturbed in a controlled manner.
- the exact sample size (n) for each experimental group/condition, given as a number, not a range;
- a description of the sample collection allowing the reader to understand whether the samples represent technical or biological replicates (including how many animals, litters, cultures, etc.).
- a statement of how many times the experiment shown was independently replicated in the laboratory.
- definitions of statistical methods and measures:
  - common tests, such as t-test (please specify whether paired vs. unpaired), simple χ2 tests, Wilcoxon and Mann-Whitney tests, can be unambiguously identified by name only, but more complex techniques should be described in the methods section;
  - are tests one-sided or two-sided?
  - are there adjustments for multiple comparisons?
  - exact statistical test results, e.g., P values = x but not P values < x;
  - definition of 'center values' as median or average;
  - definition of error bars as s.d. or s.e.m.

Please complete ALL of the questions below.
Select "Not Applicable" only when the requested information is not relevant for your study.

Materials

Newly Created Materials	Information included in the manuscript?	In which section is the information available? (Reagents and Tools Table, Materials and Methods, Figures, Data Availability Section)
New materials and reagents need to be available; do any restrictions apply?	Yes	Reagents and Tools Table

Antibodies	Information included in the manuscript?	In which section is the information available? (Reagents and Tools Table, Materials and Methods, Figures, Data Availability Section)
For **antibodies** provide the following information:<br>- Commercial antibodies: RRID (if possible) or supplier name, catalogue number and or/clone number<br>- Non-commercial: RRID or citation	Yes	Reagents and Tools Table

DNA and RNA sequences	Information included in the manuscript?	In which section is the information available? (Reagents and Tools Table, Materials and Methods, Figures, Data Availability Section)
**Short novel DNA or RNA including primers, probes**: provide the sequences.	Yes	Reagents and Tools Table

Cell materials	Information included in the manuscript?	In which section is the information available? (Reagents and Tools Table, Materials and Methods, Figures, Data Availability Section)
**Cell lines:** Provide species information, strain. Provide accession number in repository **OR** supplier name, catalog number, clone number, and/**OR** RRID.	Yes	Reagents and Tools Table
**Primary cultures:** Provide species, strain, sex of origin, genetic modification status.	Yes	Methods
Report if the cell lines were recently **authenticated** (e.g., by STR profiling) and tested for mycoplasma contamination.	Not Applicable	

Experimental animals	Information included in the manuscript?	In which section is the information available? (Reagents and Tools Table, Materials and Methods, Figures, Data Availability Section)
**Laboratory animals or Model organisms:** Provide species, strain, sex, age, genetic modification status. Provide accession number in repository **OR** supplier name, catalog number, clone number, **OR** RRID.	Yes	Reagents and Tools Table
**Animal observed in or captured from the field:** Provide species, sex, and age where possible.	Not Applicable	
Please detail **housing and husbandry conditions**.	Yes	Methods

Plants and microbes	Information included in the manuscript?	In which section is the information available? (Reagents and Tools Table, Materials and Methods, Figures, Data Availability Section)
**Plants:** provide species and strain, ecotype and cultivar where relevant, unique accession number if available, and source (including location for collected wild specimens).	Not Applicable	
**Microbes:** provide species and strain, unique accession number if available, and source.	Not Applicable	

Human research participants	Information included in the manuscript?	In which section is the information available? (Reagents and Tools Table, Materials and Methods, Figures, Data Availability Section)
If collected and within the bounds of privacy constraints report on age, sex and gender or ethnicity for all study participants.	Not Applicable	

Core facilities	Information included in the manuscript?	In which section is the information available? (Reagents and Tools Table, Materials and Methods, Figures, Data Availability Section)
If your work benefited from core facilities, was their service mentioned in the acknowledgments section?	Yes	Acknowledgments

Design

Study protocol	Information included in the manuscript?	In which section is the information available? (Reagents and Tools Table, Materials and Methods, Figures, Data Availability Section)
If study protocol has been **pre-registered, provide DOI in the manuscript**. For clinical trials, provide the trial registration number **OR** cite DOI.	Not Applicable	
Report the **clinical trial registration number** (at ClinicalTrials.gov or equivalent), where applicable.	Not Applicable	

Laboratory protocol	Information included in the manuscript?	In which section is the information available? (Reagents and Tools Table, Materials and Methods, Figures, Data Availability Section)
Provide DOI OR other citation details if **external detailed step-by-step protocols** are available.	Not Applicable	

Experimental study design and statistics	Information included in the manuscript?	In which section is the information available? (Reagents and Tools Table, Materials and Methods, Figures, Data Availability Section)
Include a statement about **sample size** estimate even if no statistical methods were used.	Not Applicable	
Were any steps taken to minimize the effects of subjective bias when allocating animals/samples to treatment (e.g. **randomization procedure**)? If yes, have they been described?	Not Applicable	
Include a statement about **blinding** even if no blinding was done.	Not Applicable	
Describe **inclusion/exclusion criteria** if samples or animals were excluded from the analysis. Were the criteria pre-established?<br><br>If sample or data points were omitted from analysis, report if this was due to attrition or intentional exclusion and provide justification.	Not Applicable	
For every figure, are **statistical tests** justified as appropriate? Do the data meet the assumptions of the tests (e.g., normal distribution)? Describe any methods used to assess it. Is there an estimate of variation within each group of data? Is the variance similar between the groups that are being statistically compared?	Yes	Figure Legends

Sample definition and in-laboratory replication	Information included in the manuscript?	In which section is the information available? (Reagents and Tools Table, Materials and Methods, Figures, Data Availability Section)
In the figure legends: state number of times the experiment was **replicated** in laboratory.	Yes	Figure Legends
In the figure legends: define whether data describe **technical or biological replicates**.	Yes	Figure Legends

Ethics

Ethics	Information included in the manuscript?	In which section is the information available? (Reagents and Tools Table, Materials and Methods, Figures, Data Availability Section)
Studies involving **human participants**: State details of **authority granting ethics approval** (IRB or equivalent committee(s), provide reference number for approval.	Not Applicable	
Studies involving **human participants**: Include a statement confirming that **informed consent** was obtained from all subjects and that the experiments conformed to the principles set out in the WMA Declaration of Helsinki and the Department of Health and Human Services Belmont Report.	Not Applicable	
Studies involving **human participants:** For publication of **patient photos**, include a statement confirming that consent to publish was obtained.	Not Applicable	
Studies involving experimental **animals**: State details of **authority granting ethics approva**l (IRB or equivalent committee(s), provide reference number for approval. Include a statement of compliance with ethical regulations.	Yes	Methods
Studies involving **specimen and field samples:** State if relevant **permits** obtained, provide details of authority approving study; if none were required, explain why.	Not Applicable	

Dual Use Research of Concern (DURC)	Information included in the manuscript?	In which section is the information available? (Reagents and Tools Table, Materials and Methods, Figures, Data Availability Section)
Could your study fall under dual use research restrictions? Please check biosecurity documents and list of **select agents and toxins** (CDC): https://www.selectagents.gov/sat/list.htm	Not Applicable	
If you used a select agent, is the security level of the lab appropriate and reported in the manuscript?	Not Applicable	
If a study is subject to dual use research of concern regulations, is the name of the **authority granting approval and reference number** for the regulatory approval provided in the manuscript?	Not Applicable	

Reporting

The MDAR framework recommends adoption of discipline-specific guidelines, established and endorsed through community initiatives. Journals have their own policy about requiring specific guidelines and recommendations to complement MDAR.

Adherence to community standards	Information included in the manuscript?	In which section is the information available? (Reagents and Tools Table, Materials and Methods, Figures, Data Availability Section)
State if relevant guidelines or checklists (e.g., **ICMJE, MIBBI, ARRIVE, PRISMA**) have been followed or provided.	Not Applicable	
For **tumor marker prognostic studies,** we recommend that you follow the **REMARK** reporting guidelines (see link list at top right). See author guidelines, under 'Reporting Guidelines'. Please confirm you have followed these guidelines.	Not Applicable	
For **phase II and III randomized controlled trials**, please refer to the **CONSORT** flow diagram (see link list at top right) and submit the CONSORT checklist (see link list at top right) with your submission. See author guidelines, under 'Reporting Guidelines'. Please confirm you have submitted this list.	Not Applicable	

Data Availability

Data availability	Information included in the manuscript?	In which section is the information available? (Reagents and Tools Table, Materials and Methods, Figures, Data Availability Section)
Have **primary datasets** been deposited according to the journal's guidelines (see 'Data Deposition' section) and the respective accession numbers provided in the Data Availability Section?	Yes	Data Availability
Were **human clinical and genomic datasets** deposited in a public access-controlled repository in accordance to ethical obligations to the patients and to the applicable consent agreement?	Not Applicable	
Are **computational models** that are central and integral to a study available without restrictions in a machine-readable form? Were the relevant accession numbers or links provided?	Yes	Data Availability
If publicly available data were reused, provide the respective **data citations in the reference list**.	Not Applicable	
